# Chemical Synthesis of 6-Azido-6-Deoxy Derivatives of Phosphatidylinositol Containing Different Fatty Acid Chains

**DOI:** 10.3390/molecules29214981

**Published:** 2024-10-22

**Authors:** Mosidur Rahaman Molla, Palak Gupta, Rajendra Rohokale, Zhongwu Guo

**Affiliations:** 1 Department of Chemistry, University of Florida, Gainesville, FL 32611, USArajendra.rohokal@chem.ufl.edu (R.R.); 2 UF Health Cancer Center, University of Florida, Gainesville, FL 32611, USA

**Keywords:** azide, glycolipid, glycosylphosphatidylinositol, inositol, phosphatidylinositol

## Abstract

This paper describes the synthesis of two 6-azido-6-deoxy derivatives of phosphatidylinositol (PI), which contained different fatty acid chains. These syntheses, starting from methyl α-d-glucopyranoside, employed multiple regioselective transformations with Ferrier rearrangement as one of the key steps. The PI derivatives contained different fatty acid chains in the lipids and an azido group in the inositol residue to facilitate their further functionalization under bioorthogonal conditions. Therefore, they should be useful probes for the investigation of PI and related biology, such as PI phosphorylation, PI interaction with other molecules in cells, and the functions of lipid structures in these processes.

## 1. Introduction

**Figure 1:**
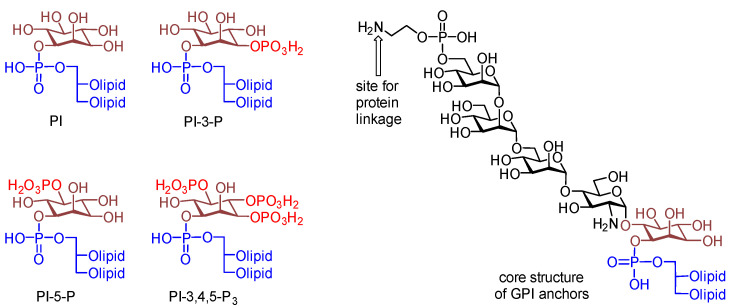
Chemical structures of PI, PI-3-P, PI-5-P, PI-3,4,5-P_3_, and the conserved core structure of GPI anchors.

Lipids are essential constituents of the cell membrane and play a critical role in many biological processes [1,2]. For example, phosphatidylinositol (PI) and its various phosphorylated forms, such as PI-3-phosphate (PI-3-P), PI-5-P and PI-3,4,5-P_3_ (Figure 1), are a class of lipids involved in not only structural modulations of the cell membrane but also the regulation of molecular transport and cellular signaling [3,4]. The unique and adaptable properties of PI have stimulated considerable enthusiasm for its structural investigation and modification to result in innovative lipid analogues with customized functionalities. These studies have broadened the range of lipid research and related biological applications [5]. Moreover, PI is also an important part in the core structure of glycosylphosphatidylinositol (GPI) anchors and GPI-anchored proteins (GPI-APs) [6,7].

To study the functional roles and mechanisms of PIs in various biological processes, appropriate methods and molecular tools to enable specific labeling and examination of these biomolecules on cells are imperative. To this end, our group previously developed approaches to metabolically engineer cell-surface GPIs and GPI-APs to carry an azido tag using azide-modified *myo*-inositol and PI derivatives [8,9,10]. This enabled the functionalization of GPIs and GPI-APs with various molecular labels for their biological studies and applications. In the reported inositol and PI derivatives, the azide is linked to the 4-*C*-position of inositol, as it is the furthest site from the 1,2,6-*C*-positions involved in GPI biosynthesis—a design aimed to cause minimal interference to their metabolic incorporation by cells [8,11]. However, these inositol and PI derivatives are not suitable for the investigation of PIPs, as their 4-*C*-position that is critical for PIP biosynthesis [12] is modified. The current work aims to design new PI derivatives as probes for the study of PIPs and to develop efficient methods for the synthesis of these probes.

## 2. Results and Discussion

**Figure 2:**
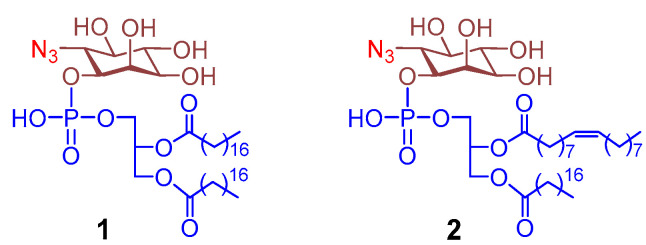
Structures of the designed azido-PI analogues **1** and **2.**

As mentioned above, the 3,4,5-*C*-positions of *myo*-inositol in PI are critical for its biosynthetic transformation into various PIPs (Figure 1). Therefore, PI derivatives useful for metabolic PIP engineering should have these positions undisrupted. Under this condition, the only sites left in the *myo*-inositol residue of PI for modifications are the 2- and 6-*C*-positions. However, the 2-*C*-position of *myo*-inositol is important for its distinction from other inositols, because only the hydroxyl group at this position is axial [13]. Accordingly, we designed PI derivatives **1** and **2** (Figure 2) having the inositol 6-OH group in PI replaced with an azido group as molecular tools for the metabolic engineering of PIPs. The azide in **1** and **2** will enable further modifications of these PI derivatives and their phosphorylated products for many biological applications. In the meantime, since the 6-*C*-position in **1** and **2** is blocked, but this position is required for GPI biosynthesis, these probes would not participate in GPI and GPI-AP metabolism [11], thereby showing selectivity for metabolic PIP engineering. Additionally, these probes are designed to contain different fatty acid chains (saturated vs. unsaturated) in the lipid, which can be used to investigate the impacts of lipid structures on the biosynthesis and biological functions of PIs and PIPs. In addition to the above properties, these new probes also have several other advantages over previously reported PI probes. For example, the azide group is relatively small, not much larger than a hydroxyl group, but definitely much smaller than any available fluorescent, radical, or affinity tags, which can minimize the steric hindrance during their interaction with other biomolecules, such as the enzymes involved in PI metabolism. In the meantime, the azide group is rather similar to the hydroxyl group in terms of polarity. Therefore, azides are widely utilized to replace hydroxyl groups in carbohydrates to mimic natural products for glycoengineering of cells and enzymatic synthesis of oligosaccharides.

**Scheme 1:**
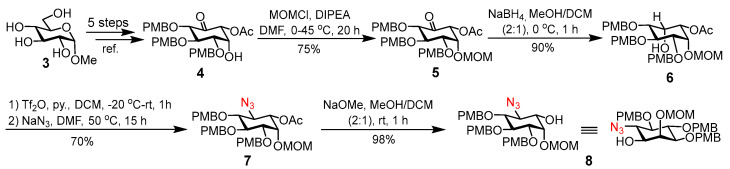
Synthesis of 6-azido-6-deoxy-inositol derivative **8.**

We commenced the synthesis of **1** and **2** with preparing the common key azido intermediate **8** (Scheme 1). First, methyl α-d-glucopyranoside **3** was smoothly converted into ketone **4** by a previously described procedure [14,15,16]. Here, the *para*-methoxybenzyl (PMB) group was utilized for global protection of hydroxyl groups, because PMB ethers can be easily deprotected under conditions that do not affect the azide, *O*-acyl groups, and unsaturated lipids in structures of the final products. The free hydroxyl group in **4** was then protected with a methoxymethyl (MOM) group to afford fully protected ketone **5**. MOM was chosen since it can be introduced under conditions that would not interfere with other protecting groups in heavily substituted **4**. Subsequent reduction of the keto group in **5** using NaBH_4_ was highly stereoselective, resulting in alcohol **6** as the single diastereomer in an excellent yield (90%) [15]. Azide was then introduced by a two-step protocol, including triflation of the hydroxyl group using triflic anhydride (Tf_2_O) and azide substitution of the triflate by S_N_2 mechanism, to afford **7**. Finally, the *O*-acetyl group in **7** was chemoselectively removed with NaOMe to provide the key intermediate 6-azido-6-deoxy-inositol **8**, which was utilized to synthesize the target molecules [14].

**Scheme 2:**
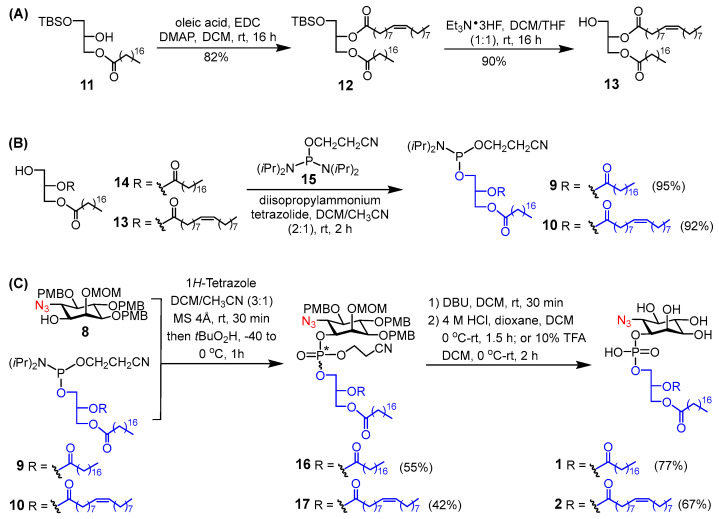
Synthesis of (**A**) lipid **13**, (**B**) phosphoramidites **9** and **10**, and (**C**) the target PI derivatives **1** and **2**.

Once the 6-azido-inositol derivative **8** was available, we turned our attention to the installation of phospholipids at its 1-*O*-position. We planned to accomplish this transformation by the well-established two-step one-pot protocol [17] using phosphoramidites **9** and **10** (Scheme 2) as the phosphorylating reagents. Both phosphoramidites were synthesized by a reported protocol [18] outlined in Scheme 2A,B. Condensation of alcohol **11** with oleic acid promoted by 1-ethyl-3-(3-dimethylaminopropyl)carbodiimide (EDC) and 4-(dimethylamino)pyridine (DMAP) gave **12**, which was followed by selective removal of the tert-butyldimethylsilyl (TBS) group with triethylamine trihydrofluoride (Et_3_N·3HF) to afford lipid **13**. Finally, reactions of **13** and commercial **14** with 2-cyanoethyl *N,N,N′,N′-*tetraisopropylphosphorodiamidite **15** in the presence of diisopropylammonium tetrazolide provided **9** and **10**, respectively.

Phospholipidation of **8** was accomplished by a reaction with phosphoramidites **9** and **10** in the presence of 1*H*-tetrazole and then in situ oxidation of the resultant phosphites using *t*-BuO_2_H, to afford **16** and **17**, respectively, as 1:1 inseparable diastereomeric mixtures due to the presence of a phosphorous stereogenic center in their structure. Interestingly, the ^31^P NMR spectrum of **17** showed two sets of four signals, like that observed with similar products in the literature [19]. We believe that this is due to the existence of rotamers resulting from the high steric hinderance caused by the protecting groups and rigid fatty acyl chains in **17**, which was verified by the disappearance of the multiplet property of the ^31^P NMR signal after global deprotection to show a single peak for **2**. We believe that this may cause their NMR spectra to be rather complex. It should be also pointed out that as phosphoramidites **9** and **10** were highly reactive and can decompose quickly, they were used in excess in the reaction [11]. Finally, global deprotection of **16** and **17** was carried out in two steps, including removal of the cyanoethyl group protecting the phosphate moiety with 1,8-diazabicyclo [5.4.0]undec-7-ene (DBU) and deprotection of the MOM and PMB ethers with HCl in dioxane or with 10% trifluoroacetic acid (TFA) [17,20], provided the desired PI analogues **1** and **2**, whose purities were >96%, as determined by high-performance liquid chromatography (HPLC) (Figure S50, SI). The final products and all new synthetic intermediates were fully characterized with NMR and HR-MS data.

## 3. Materials and Methods

**General methods:** Commercial chemicals and materials were used without further purification unless specified otherwise. Flash chromatography used silica gel 60 (230–400 mesh). Thin-layer chromatography (TLC) was performed on silica gel 60G F_254_ 25 glass plates detected with a UV lamp, with charring using 10% (*v*/*v*) H_2_SO_4_ in ethanol, and staining using *p*-anisaldehyde. ^1^H, ^13^C, COSY, and HSQC NMR spectra were obtained with Bruker (Billerica, MA, USA) 400 and 600 MHz NMR spectrometers. Coupling constants (*J*) were reported in hertz (Hz). Chemical shifts (δ) were reported in ppm referenced to CDCl_3_ (^1^H NMR: δ 7.26 ppm; ^13^C NMR: δ 77.00 ppm) or CD_3_OD (^1^H NMR: δ 3.31 ppm; ^13^C NMR: δ 49.0 ppm). The chemical shifts of ^31^P NMR signals were calculated using a lock signal in reference to 85% phosphoric acid in water. Abbreviations that describe peak splitting patterns are as follows: s, singlet; d, doublet; t, triplet; dd, double doublet; m, multiplet; br, broad. High-resolution electrospray ionization mass spectra (HR ESI MS) were obtained with an Agilent (Santa Clara, CA, USA) 6230 time-of-flight (TOF) machine. Aluminum heating blocks were used to heat the reaction mixtures.

**(2R,3R,4S,5R,6S)-2-Acetoxy-4,5,6-tris[(4-methoxybenzyl)oxy]-3-methoxymethoxy-cyclohexan-1-one (5).** To a solution of **4** [16] (100 mg, 0.172 mmol) in anhydrous DMF (0.6 mL), DIPEA (207 µL, 1.19 mmol) was added at 0 °C, which was followed by the addition of MOMCl (65 µL, 0.861 mmol). The reaction mixture was heated to 45 °C and stirred at this temperature for 20 h. The reaction mixture was diluted with water and extracted with DCM (10 mL × 3). The organic layers were combined, dried with Na_2_SO_4_, filtered, and condensed under reduced pressure. The crude product was purified by silica gel column chromatography to afford **5** (81 mg, 75%) as a colorless syrup. R_f_ = 0.40 (40% EtOAc in hexane); ^1^H NMR (600 MHz, CDCl_3_): δ 7.32 (d, *J* = 8.6 Hz, 2H), 7.27 (d, *J* = 9.3 Hz, 2H), 7.24 (d, *J* = 8.6 Hz, 2H), 6.89–6.83 (m, 6H), 5.15 (d, *J* = 2.4 Hz, 1H), 4.85 (d, *J* = 10.9 Hz, 1H), 4.80–4.77 (m, 2H), 4.73 (d, *J* = 10.3 Hz, 1H), 4.72–4.68 (m, 2H), 4.64 (d, *J* = 11.2 Hz, 1H), 4.48 (d, *J* = 10.9 Hz, 1H), 4.34 (t, *J* = 2.3 Hz, 1H), 4.08–4.04 (m, 2H), 3.82–3.79 (m, 10H), 3.39 (s, 3H), 2.21 (s, 3H); ^13^C NMR (151 MHz, CDCl_3_): δ 197.8, 169.8, 159.4, 159.4, 159.2, 130.6, 129.9, 129.7, 129.6, 129.5, 113.9, 113.8, 113.7, 96.8, 83.5, 81.9, 79.0, 75.7, 75.2, 73.4, 72.8, 72.6, 55.7, 55.3, 55.24, 55.23, 20.6; HR-ESI-TOF MS *m/z*: Calcd for C_34_H_40_NaO_11_ [M + Na]^+^ 647.2468; Found 647.2464.

**3-O-Acetyl-1,5,6-tri-O-(4-methoxybenzyl)-2-O-methoxymethyl-epi-inositol (6).** A mixture of **5** (310 mg, 0.496 mmol) and NaBH_4_ (28 mg, 0.744 mmol) in MeOH and DCM (2:1, 6 mL) was stirred at 0 °C for 1 h, and then saturated aqueous NH_4_Cl solution was added. The reaction mixture was diluted with DCM, dried over Na_2_SO_4_, filtered, and then concentrated in vacuo. The residue was purified by silica gel column chromatography (EtOAc/hexane, 2:1) to provide **6** (280 mg, 90%) as a white solid. R_f_ = 0.30 (50% EtOAc in hexane); ^1^H NMR (600 MHz, CDCl_3_): δ 7.34–7.24 (m, 7H), 6.90–6.85 (m, 6H), 4.88–4.83 (m, 2H), 4.79 (d, *J* = 10.2 Hz, 1H), 4.74 (d, *J* = 6.9 Hz, 1H), 4.71 (d, *J* = 11.4 Hz, 1H), 4.67–4.62 (m, 3H), 4.61 (t, *J* = 2.9 Hz, 1H), 4.33 (d, *J* = 1.8 Hz, 1H), 4.21 (d, *J* = 9.4 Hz, 1H), 4.11 (t, *J* = 9.8 Hz, 1H), 3.83 (s, 3H), 3.82 (s, 3H), 3.82 (s, 3H), 3.46 (s, 3H), 3.42 (dd, *J* = 9.8, 2.7 Hz, 1H), 3.38 (dd, *J* = 9.8, 3.3 Hz, 1H), 2.18 (s, 3H); ^13^C NMR (151 MHz, CDCl_3_): δ 170.2, 159.3, 159.2, 159.15, 131.1, 130.1, 130.0, 129.7, 129.5, 129.4, 113.8, 113.79, 113.7, 97.8, 79.9, 79.5, 78.4, 76.1, 75.8, 72.9, 71.9, 70.2, 68.9, 56.0, 55.3, 55.2, 21.0; HR-ESI-TOF MS *m/z*: Calcd for C_34_H_46_NO_11_ [M + NH_4_]^+^ 644.3065; Found 644.3063.

**1-O-Acetyl-6-azido-6-deoxy-3,4,5-tri-O-(p-methoxybenzyl)-2-O-methoxymethyl-myo-inositol (7).** To a solution of **6** (330 mg, 0.526 mmol) and pyridine (85 µL, 1.05 mmol) in DCM (5 mL), Tf_2_O (104 µL, 0.631 mmol) was added at −20 °C. The solution was allowed to warm at room temperature (rt) and was stirred for 1 h. After the disappearance of the starting material as shown by TLC, the reaction was quenched with saturated aqueous NaHCO_3_. The mixture was extracted with DCM, and the organic layers were combined, washed with brine, and dried over Na_2_SO_4_. The solvent was evaporated to provide crude triflate, which was dissolved in DMF (2 mL) and then treated with NaN_3_ (135 mg, 2.08 mmol, 4 equiv) at 40 °C overnight. The reaction mixture was diluted with ethyl acetate and washed with water and brine. The organic layer was dried over Na_2_SO_4,_ filtered, and concentrated under reduced pressure. The crude product was purified by silica gel column chromatography to afford **7** as a colorless syrup (240 mg, 70%). R_f_ = 0.50 (40% EtOAc in Hexane); ^1^H NMR (600 MHz, CDCl_3_): δ 7.30–7.21 (m, 6H), 6.89–6.83 (m, 6H), 4.86–4.77 (m, 3H), 4.76–4.71 (m, 2H), 4.66 (d, *J* = 6.8 Hz, 1H), 4.62 (d, *J* = 11.0 Hz, 1H), 4.56 (dd, *J* = 10.9, 2.4 Hz, 1H), 4.54 (d, *J* = 11.1 Hz, 1H), 4.23 (t, *J* = 2.2 Hz, 1H), 3.96 (d, *J* = 10.6 Hz, 1H), 3.93 (d, *J* = 9.8 Hz, 1H), 3.81 (s, 3H), 3.80 (s, 6H), 3.41 (dd, *J* = 9.9, 2.2 Hz, 1H), 3.39 (s, 3H), 3.29 (t, *J* = 9.5 Hz, 1H), 2.16 (s, 3H); ^13^C NMR (151 MHz, CDCl_3_): δ 170.1, 159.3, 159.3, 159.2, 130.8, 130.1, 129.8, 129.8, 129.5, 129.4, 113.84, 113.8, 113.77, 97.5, 81.2, 81.1, 79.6, 75.5, 75.49, 72.5, 72.4, 71.7, 63.4, 55.7, 55.3, 20.9; HR-ESI-TOF MS *m/z*: Calcd for C_34_H_45_N_4_O_10_ [M + NH_4_]^+^ 669.3130; Found 669.3134.

**6-Azido-6-deoxy-3,4,5-tri-O-(p-methoxybenzyl)-2-O-(methoxymethyl)-myo-inositol (8).** To a solution of **7** (26 mg, 0.040 mmol) in MeOH and DCM (2:1, 1 mL), NaOMe (5 M in MeOH, 10 µL) was added at rt. After stirring the solution at rt for 2 h, Amberlite IR 120 H^+^ resin was added, followed by filtration and condensation under reduced pressure. The crude product was purified by silica gel column chromatography to afford **8** (25 mg, 98%) as a white solid. R_f_ = 0.35 (40% EtOAc in hexane); ^1^H NMR (600 MHz, CDCl_3_): δ 7.31–7.22 (m, 6H), 6.91–6.82 (m, 6H), 4.84 (d, *J* = 10.4 Hz, 1H), 4.83 (d, *J* = 6.8 Hz, 1H), 4.80 (d, *J* = 10.1 Hz, 1H), 4.74 (d, *J* = 10.2 Hz, 2H), 4.66 (d, *J* = 6.7 Hz, 1H), 4.64 (d, *J* = 11.4 Hz, 1H), 4.59 (d, *J* = 11.4 Hz, 1H), 3.92 (t, *J* = 2.4 Hz, 1H), 3.90 (t, *J* = 9.5 Hz, 1H), 3.81 (s, 6H), 3.80 (s, 3H), 3.62 (t, *J* = 10.1 Hz, 1H), 3.55 (d, *J* = 8.5 Hz, 1H), 3.45 (s, 3H), 3.37 (dd, *J* = 9.9, 2.6 Hz, 1H), 3.29–3.20 (m, 2H); ^13^C NMR (151 MHz, CDCl_3_): δ 159.4, 159.3, 159.2, 130.8, 130.2, 129.9, 129.8, 129.5, 129.47, 113.9, 113.8, 113.79, 98.6, 81.4, 81.3, 79.4, 79.2, 75.5, 75.4, 72.7, 70.3, 66.9, 56.1, 55.3; HR-ESI-TOF MS *m/z*: Calcd for C_32_H_43_N_4_O_9_ [M + NH_4_]^+^ 627.3025; Found 627.3032.

**(R)-1-[(tert-butyldimethylsilyl)oxy]-3-(stearoyloxy)propan-2-yl (E)-octadec-9-enoate (12).** To a solution of **11** [18,21] (0.20 g, 0.42 mmol) and oleic acid (0.3 mL, 0.97 mmol) in DCM (5.0 mL), EDC (0.17 g, 1.14 mmol) and DMAP (15.5 mg, 0.13 mmol) were added under a N_2_ atmosphere. The solution was stirred in the dark at rt for 16 h. The reaction mixture was diluted with water and extracted with DCM three times. The combined organic layers were washed with brine, dried over Na_2_SO_4_, and concentrated under reduced pressure. The crude product was purified by column chromatography with 4% EtOAc in hexane as the eluent to give **12** (0.25 g, 82%) as colorless syrup. R_f_ = 0.45 (8% EtOAc in Hex); ^1^H NMR (400 MHz, CDCl_3_): δ 5.39–5.30 (m, 2H), 5.10–5.05 (m, 1H), 4.34 (dd, *J* = 11.8, 3.7 Hz, 1H), 4.16 (dd, *J* = 11.9, 6.3 Hz, 1H), 3.75–3.67 (m, 2H), 2.32–2.28 (m, 4H), 2.03–1.98 (m, 4H), 1.63–1.59 (m, 4H), 1.35−1.25 (m, 48H), 0.90–0.86 (m, 15H), 0.05 (s, 6H, SiMe_2_). The ^1^H NMR data match with those reported in the literature [22].

**(S)-3-Hydroxy-1-(stearoyloxy)propan-2-yl (E)-octadec-9-enoate (13).** Et_3_N·3HF (0.68 mL, 4.17 mmol) was slowly added to a solution of **12** (0.26 g, 0.35 mmol) in CH_3_CN and THF (1:1, 6.0 mL). After the solution was stirred at rt for 16 h, the reaction was quenched by dropwise addition of saturated aqueous NaHCO_3_ at 0 °C. The aqueous layer was extracted with DCM three times. The combined organic layers were washed with brine, dried over Na_2_SO_4_, and concentrated under vacuum. The crude product was purified by column chromatography with 12% EtOAc in hexane as the eluent to afford **13** (0.20 g, 90%) as colorless oil. R_f_ = 0.38 (20% EtOAc in Hex); ^1^H NMR (400 MHz, CDCl_3_): δ 5.35−5.31 (m, 2H), 5.08 (p, *J* = 5.0 Hz, 1H), 4.32 (dd, *J* = 11.9, 4.6 Hz, 1H), 4.24 (dd, *J* = 11.9, 5.6 Hz, 1H), 3.73 (t, *J* = 5.3 Hz, 2H), 2.37–2.31 (m, 4H), 2.06–1.98 (m, 4H), 1.62–1.60 (m, 4H), 1.34−1.25 (m, 48H), 0.87 (t, *J* = 8.0 Hz, 6H, CH_3_). The ^1^H NMR data match with those reported in the literature [23].

**(2-Cyanoethoxyl)-(diisopropylamino)-[(R)-2-O-oleoyl-3-O-stearoyl-sn-glycerol)]-phosphine (10).** To a solution of **13** (0.20 g, 0.31 mmol) and commercial **16** (0.30 mL, 0.94 mmol) in dry DCM and CH_3_CN (2:1, 3.0 mL), diisopropylammonium tetrazolide (161 mg, 0.94 mmol) was added at rt under a nitrogen atmosphere. After stirring for 2 h, the reaction mixture was diluted with DCM and poured into a saturated aqueous NaHCO_3_ solution. The mixture was extracted with DCM three times, and the combined organic layers were dried with Na_2_SO_4_ and concentrated under reduced pressure. The crude product was purified by column chromatography using triethylamine-neutralized silica gel with 10% EtOAc in hexane containing ~1% triethylamine as the eluent to provide **10** (0.24 g, 92%) as a colorless syrup. R_f_ = 0.33 (20% EtOAc in Hex); ^1^H NMR (400 MHz, CDCl_3_): 5.32–5.20 (m, 2H), 5.15–5.09 (m, 1H), 4.31–4.22 (m, 1H), 4.13–4.05 (m, 1H), 3.82–3.68 (m, 3H), 3.65–3.59 (m, 1H), 3.54–3.48 (m, 2H), 2.57 (t, *J* = 6.4 Hz, 2H), 2.28–2.22 (m, 4H), 1.96–1.91 (m, 4H), 1.57–1.52 (m, 4H), 1.26−1.18 (m, 54H), 1.11 (dd, *J* = 6.9, 5.0 Hz, 12H), 0.81 (t, *J* = 6.7 Hz, 7H); ^31^P NMR (CDCl_3_, 162 MHz): δ 149.51, 149.36. The ^1^H NMR data match with those reported in the literature [23].

**(2-Cyanoethoxyl)-(diisopropylamino)-[(R)-2,3-di-O-stearoyl-sn-glycerol)]-phosphine (9).** It was synthesized from commercial **14** in a 95% yield by the same method utilized to synthesize **10**, and its ^1^H NMR data match those reported in the literature [11].

**6-Azido-1-O-{(2-cyanoethoxy)-[(R)-2,3-di-O-stearoyl-sn-glycerol]-phosphono}-6-deoxy-3,4,5-tri-O-(4-methoxybenzyl)-2-O-(methoxymethyl)-myo-inositol (16).** To a solution of **8** (15 mg, 0.024 mmol) in DCM and CH_3_CN (4:1, 1.5 mL), activated MS 4Å (50 mg) and 1*H*-tetrazole (0.45 M in acetonitrile, 0.53 mL, 0.236 mmol) were added at rt. This was followed by dropwise addition of **9** (72 mg, 0.086 mmol) dissolved in DCM (0.5 mL). After 30 min of stirring, the mixture was cooled to 0 °C, and then *t*-BuO_2_H (105 µL, 0.577 mmol) was added. After 1 h of stirring, the mixture was diluted with DCM and washed with saturated aqueous NaHCO_3_. The aqueous layer was extracted with DCM three times. The organic layers were combined, washed with brine, dried with Na_2_SO_4_, filtered, and concentrated under reduced pressure. The crude product was purified by silica gel column chromatography to afford **16** (15 mg, 55%) as a white solid. R_f_ = 0.30 (40% EtOAc in hexane); ^1^H NMR (400 MHz, CDCl_3_): δ 7.31–7.20 (m, 8H), 6.90–6.82 (m, 4H), 5.35–5.25 (m, 1H), 4.90–4.80 (m, 3H), 4.78–4.62 (m, 4H), 4.54 (dd, *J* = 10.9, 1.8 Hz, 1H), 4.41–4.12 (m, 6H), 4.11–4.00 (m, 1H), 3.93 (td, *J* = 9.9, 2.6 Hz, 2H), 3.81 (m, 9H), 3.41–3.34 (m, 3H), 3.29 (td, *J* = 9.4, 2.9 Hz, 1H), 2.87–2.73 (m, 2H), 2.46–2.21 (m, 4H), 1.67–1.53 (m, 8H), 1.45–1.10 (m, 56H), 0.91–0.83 (m, 6H); ^13^C NMR (151 MHz, CDCl_3_): δ 173.2, 172.9, 159.3, 159.3, 159.2, 130.7, 129.9, 129.9, 129.8, 129.5, 116.3, 116.25, 113.8, 113.78, 113.77, 97.6, 97.5, 81.0, 80.9, 79.33, 79.3, 75.6, 75.5, 73.5, 73.3, 72.5, 72.4, 69.2, 64.3, 61.5, 55.94, 55.9, 55.3, 34.1, 34.0, 31.9, 29.7, 29.65, 29.5, 29.49, 29.4, 29.3, 29.29, 29.13, 29.12, 24.8, 22.7, 14.1; ^31^P NMR (162 MHz, CDCl_3_): δ −2.06, −2.12; HR-ESI-TOF MS *m/z*: Calcd for C_74_H_121_N_5_O_16_P [M + NH_4_]^+^ 1366.8541; Found 1366.8567.

**6-Azido-1-O-[(2-cyanoethoxyl)-[(R)-2-O-oleoyl-3-O-stearoyl-sn-glycerol)]-phosphono-6-deoxy-3,4,5-tri-O-4-methoxybenzyl-2-O-(methoxymethyl)-myo-inositol (17).** To a solution of **8** (35 mg, 0.057 mmol) in dry DCM and CH_3_CN (3:1, 6.4 mL), MS 4Å and 1*H*-tetrazole (0.45 M in acetonitrile, 1.26 mL, 0.57 mmol) were added, followed by dropwise addition of a fresh solution of **10** (0.24 g, 0.28 mmol) in DCM under nitrogen at rt. After the mixture was stirred at rt for 30 min, it was cooled to −40 °C and treated with *t*-BuO_2_H (5.5 M in decane, 0.21 mL, 1.14 mmol). After the solution was stirred at −40 °C for 1 h, Me_2_S (0.17 mL, 2.28 mmol) was added, with stirring for 1 h. While the reaction mixture was slowly warmed to rt, it was diluted with DCM and then filtered through a Celite pad. The mixture was poured into saturated aqueous NaHCO_3_ solution and extracted with DCM three times. The organic layers were combined, dried with Na_2_SO_4_, filtered, and condensed under reduced pressure. The crude product was purified by silica gel column chromatography with 12% EtOAc in toluene as the eluent to give **17** (33 mg, 42%) as a colorless syrup. R_f_ = 0.28 (40% EtOAc in Hex); ^1^H NMR (600 MHz, CDCl_3_): δ 7.29–7.24 (m, 6H), 6.89–6.86 (m, 6H), 5.39–5.34 (m, 2H), 5.33–5.31 (m, 1H), 4.87–4.83 (m, 3H), 4.78–4.66 (m, 4H), 4.56 (dd, *J* = 10.9, 2.6 Hz, 1H), 4.40–4.25 (m, 7H), 4.24–4.17 (m, 2H), 4.11–4.07 (m, 1H), 3.83 (d, *J* = 1.7 Hz, 9H), 3.41–3.39 (m, 4H), 3.32 (dd, *J* = 11.2, 7.9 Hz, 1H), 2.83–2.80 (m, 2H), 2.39–2.32 (m, 4H), 2.04–2.01 (m, 4H), 1.65–1.61 (m, 4H), 1.36–1.25 (m, 48H), 0.90 (t, *J* = 6.9 Hz, 6H); ^13^C NMR (151 MHz, CDCl_3_): δ 173.3, 172.9, 172.8, 159.4, 159.3, 159.2, 130.7, 130.0, 129.97, 129.92, 129.8 (2C), 129.72, 129.7, 129.5 (3C), 113.9 (3C), 113.82, 113.8 (3C), 97.6, 97.57, 97.55, 81.0, 80.9, 79.4, 79.3, 76.0, 75.6, 75.5, 73.5, 73.4, 73.35, 72.5, 72.48, 72.42, 72.4, 69.3, 69.2, 66.2, 65.9, 64.4, 64.3, 62.4, 62.3, 62.2, 62.1, 61.6, 61.5, 61.49, 56.0, 55.9, 55.3 (3C), 55.27, 34.2, 34.1 (2C), 34.0 (2C), 33.98, 31.94 (2C), 31.9 (2C), 29.8 (2C), 29.74, 29.72 (multiple C), 29.70, 29.67 (3C), 29.66 (2C), 29.54 (2C), 29.5 (2C), 29.4 (2C), 29.34 (2C), 29.3 (2C), 29.24, 29.2, 29.16, 29.14 (2C), 29.1, 27.24 (2C), 27.2 (2C), 24.9, 24.8, 22.7 (2C), 19.7, 19.65, 19.6, 14.1 (2C). ^31^P NMR (CDCl_3_, 243 MHz) δ −1.93, −2.06, −2.12, −2.16. HR-ESI-TOF MS *m/z*: Calcd for C_74_H_119_N_4_O_16_P [M + NH_4_]^+^ 1364.8383; Found 1364.8367.

**6-Azido-6-deoxy-1-O-{[(R)-2,3-di-O-stearoyl-sn-glycerol]-phosphono}-myo-inositol (1).** To a solution of **16** (15 mg, 0.011 mmol) in anhydrous DCM (3 mL), DBU (2 µL, 0.013 mmol) was added. After stirring at rt for 1 h, the solvent was evaporated under reduced pressure, and the product was briefly purified by silica gel column chromatography and then dissolved in DCM (3 mL). After the solution was cooled to 0 °C, a 20% TFA solution in DCM (3 mL) was added. The solution was warmed to rt, stirred for 2 h, diluted with toluene, and condensed under reduced pressure. The residue was co-evaporated with toluene five times, and the product was washed with diethyl ether three times to afford **1** as a white solid (7.5 mg, 77%). R_f_ = 0.1 (15% methanol in DCM); ^1^H NMR (600 MHz, MeOD:CDCl_3_ = 3:2): δ 5.26 (s, 1H), 4.41 (d, *J* = 11.9 Hz, 1H), 4.21–3.98 (m, 3H), 3.86 (br, 1H), 3.77–3.54 (m, 3H), 3.40–3.34 (m, 1H), 3.23–3.13 (m, 1H), 2.43–2.17 (m, 4H), 1.67–1.54 (m, 4H), 1.42–1.06 (m, 56H), 0.87 (t, *J* = 6.9 Hz, 6H); ^13^C NMR (151 MHz, MeOD:CDCl_3_ 3:2): δ 174.0, 73.7, 73.3, 72.8, 71.1, 71.0, 70.3, 64.9, 63.9, 62.5, 54.5, 34.1, 34.0, 31.8, 29.6, 29.5, 29.4, 29.24, 29.2, 29.04, 29.0, 24.82, 24.8, 22.5, 13.8; ^31^P NMR (162 MHz, MeOD:CDCl_3_ = 3:2): δ −2.44; HR-ESI-TOF MS *m/z*: Calcd for C_45_H_90_N_4_O_12_P [M + NH_4_]^+^ 909.6287; Found 909.6282.

**6-Azido-6-deoxy-1-O-{[(R)-2-O-oleoyl-3-O-stearoyl-sn-glycerol]-phosphono}-myo-inositol (2).** DBU (7.2 µL, 0.048 mmol) was added to a solution of **17** (33 mg, 0.024 mmol) in dry DCM (5 mL) at rt. After stirring the solution for 1 h, glacial acetic acid (14 µL, 0.24 mmol) was added. The mixture was stirred for another 5 min and condensed under reduced pressure. The product was briefly purified by silica gel chromatography and then dissolved in dry DCM (6 mL), followed by dropwise addition of HCl in dioxane (4 M, 2.5 mL) at 0 °C. After the mixture was stirred for 10 min, it was warmed to rt with stirring for 1.5 h, diluted with toluene, and condensed under reduced pressure. The residue was co-evaporated with toluene six times, and the product was purified by silica gel column chromatography using 12% MeOH in DCM as the eluent to afford **2** as a white solid (14.5 mg, 67%). R_f_ = 0.18 (30% MeOH in CHCl_3_); ^1^H NMR (600 MHz, MeOD:CDCl_3_ = 3:2): δ 5.29–5.22 (m, 2H), 5.19–5.18 (m, 1H), 4.36–4.32 (m, 1H), 4.19 (br, 1H), 4.12–4.08 (m, 1H), 4.02–3.97 (m, 2H), 3.79 (br, 1H), 3.63–3.56 (m, 2H), 3.27–3.26 (m, 1H), 3.14–3.08 (m, 1H), 2.27–2.22 (m, 4H), 1.95–1.91 (m, 4H), 1.56–1.49 (m, 4H), 1.26–1.17 (m, 48H), 0.81–0.78 (m, 6H); ^13^C NMR: (151 MHz, MeOD:CDCl_3_ = 3:2): δ 173.9, 173.5, 129.8, 129.5, 73.7, 72.7, 71.1 (2C), 70.3, 65.1, 63.5, 62.4, 52.6, 34.0, 33.9, 31.8 (2C), 29.6 (2C), 29.5 (multiple C), 29.47 (3C), 29.3, 29.2, 29.15 (2C), 29.1, 29.0, 28.94, 28.9, 27.0, 26.96, 24.8, 24.7, 22.5 (3), 13.5 (2C). ^31^P NMR (243 MHz, MeOD:CDCl_3_ = 3:2): δ −4.74. HR-ESI-TOF MS *m/z*: Calcd for C_45_H_88_N_3_O_12_P [M + NH_4_]^+^ 907.6130; Found 907.6117.

## 4. Conclusions

We have designed and synthesized two azide-functionalized PI derivatives **1** and **2**. The presence of an azido group within **1** and **2** would facilitate their further functionalization to install various molecular tags/labels, while the small size of the azide group would guarantee a minimal impact on the properties of **1** and **2** as PI analogues. These properties make **1** and **2** useful tools for biological investigations and applications [24,25]. Although this paper describes only the synthesis of these probes, they are currently under biological studies in our lab. For example, **1** and **2** are utilized as biosynthetic precursors of PIPs for metabolic PIP engineering to examine PI phosphorylation and related signaling processes [12,26]. On the other hand, attaching azide to the inositol 6-*C*-position within **1** and **2** can block them from participating in GPI biosynthesis, thereby enhancing their selectivity for metabolic PIP engineering. The synthesis of **1** and **2** is highlighted by the usage of and efficient access to a common intermediate **8** having hydroxyl groups protected with the MOM and PMB groups, which are easily removable under mildly acidic conditions compatible with unsaturated lipids, azide, and various other functional groups. Therefore, this synthetic intermediate and thus-based synthetic strategy are expected to be widely applicable to differently functionalized PI derivatives.

## Data Availability

All the data underlying this research are available in the published article and its Supporting Information.

## Supplementary Materials

The following supporting information, i.e., the NMR and MS spectra of all new compounds and the HPLC results of the final synthetic targets, can be downloaded at: https://www.mdpi.com/article/10.3390/molecules29214981/s1.

## References

[1] Brown H.A., Marnett L.J. (2011). Introduction to lipid biochemistry, metabolism, and signaling. Chem. Rev..

[2] Guo R., Chen Y., Borgard H., Jijiwa M., Nasu M., He M., Deng Y. (2020). The function and mechanism of lipid molecules and their roles in the diagnosis and prognosis of breast cancer. Molecules.

[3] Dickson E.J. (2019). Recent advances in understanding phosphoinositide signaling in the nervous system. F1000Res.

[4] Batrouni A.G., Baskin J. (2021). The chemistry and biology of phosphatidylinositol 4-phosphate at the plasma membrane. Bioorg. Med. Chem..

[5] Routt S.M., Bankaitis V.A. (2004). Biological functions of phosphatidylinositol transfer proteins. Biochem. Cell Biol..

[6] Thomas J.R., Dwek R.A., Rademacher T.W. (1990). Structure; biosynthesis, and function of glycosylphosphatidylinositols. Biochemistry.

[7] Ferguson M.A.J. (1999). The structure, biosynthesis and functions of glycosylphosphatidylinositol anchors, and the contributions of trypanosome research. J. Cell Sci..

[8] Lu L., Gao J., Guo Z. (2015). Labeling cell surface GPIs and GPI-anchored proteins through cell metabolic engineering with artificial inositol derivatives. Angew. Chem. Int. Ed..

[9] Jaiswal M., Zhu S., Jiang W., Guo Z. (2020). Synthesis and evaluation of Nα,Nε-diacetyl-L-lysine-inositol conjugates for selective metabolic engineering of GPIs and GPI-anchored proteins on cancer cells. Org. Biomol. Chem..

[10] Kundu S., Jaiswal M., Craig K.C., Guo J., Guo Z. (2023). Labeling cell surface glycosylphosphatidylinositol-anchored proteins through metabolic engineering using an azide-modified phosphatidylinositol. Biochem. Biophys. Res. Commun..

[11] Craig K.C., Guo Z. (2022). Design and synthesis of 4-azido-phosphatidylinositol as a potential probe for metabolic engineering of glycosylphosphatidylinositol on cells. J. Carbohydr. Chem..

[12] Tolias K.F., Cantley L.C. (1999). Pathways for phosphoinositide synthesis. Chem. Phys. Lipids.

[13] Shashidhar M.S., Patil N.T., Tiwari V.K. (2020). Recent developments in the synthesis of biologically relevant inositol derivatives. Carbohydrates in Drug Discovery and Development.

[14] Ausmus A.P., Hogue M., Snyder J.L., Rundell S.R., Bednarz K.M., Banahene N., Swarts B.M. (2020). Ferrier carbocyclization-mediated synthesis of enantiopure azido inositol analogues. J. Org. Chem..

[15] Takahashi H., Kittaka H., Ikegami S. (2001). Novel synthesis of enantiomerically pure natural inositols and their diastereoisomers. J. Org. Chem..

[16] Estevez V.A., Prestwich G.D. (1991). Synthesis of enantiomerically pure, P-1-tethered inositol tetrakis (phosphate) affinity labels via a Ferrier rearrangement. J. Am. Chem. Soc..

[17] Swarts B.M., Guo Z. (2010). Synthesis of a glycosylphosphatidylinositol (GPI) anchor carrying unsaturated lipid chains. J. Am. Chem. Soc..

[18] Burgula S., Swarts B.M., Guo Z. (2012). Total synthesis of a glycosylphosphatidylinositol anchor of the human lymphoctye CD52 antigen. Chem. Eur. J..

[19] Anderson R.J., Osborne S.L., Meunier F.A., Painter G.F. (2010). Regioselective approach to phosphatidylinositol 3,5-bisphosphates: Syntheses of the native phospholipid and biotinylated short-chain derivative. J. Org. Chem..

[20] Hosoya T., Takashiro E., Matsumoto T., Suzuki K. (1994). Total synthesis of the gilvocarcins. J. Am. Chem. Soc..

[21] Ortuno V.E., Pulletikurti S., Veena K.S., Krishnamurthy R. (2022). Synthesis and hydrolytic stability of cyclic phosphatidic acids: Implications for synthetic- and proto-cell studies. Chem. Commun..

[22] Ardila-Fierro K.J., Pich A., Spehr M., Hernández J.G., Bolm C. (2019). Synthesis of acylglycerol derivatives by mechanochemistry. Beilstein J. Org. Chem..

[23] Shin Y.H., Bang S., Park S.M., Ma X., Cassilly C., Graham D., Xavier R., Clardy J. (2023). Revisiting Coley′s toxins: Immunogenic cardiolipins from *Streptococcus pyogenes*. J. Am. Chem. Soc..

[24] Kolb H.C., Finn M.G., Sharpless K.B. (2001). Click chemistry: Diverse chemical function from a few good reactions. Angew. Chem. Int. Ed..

[25] Sletten E.M., Bertozzi C.R. (2009). Bioorthogonal chemistry: Fishing for selectivity in a sea of functionality. Angew. Chem. Int. Ed..

[26] Downes C.P., Macphee C.H. (1990). *myo*-Inositol metabolites as cellular signals. Eur. J. Biochem..

