# Supplementary material for: Chemical Synthesis of 6-Azido-6-Deoxy Derivatives of Phosphatidylinositol Containing Different Fatty Acid Chains
Source: Molecules. 2024 Oct 22;29(21):4981. doi: 10.3390/molecules29214981 (PMC11547759; doi:10.3390/molecules29214981)
Supplement: Supplementary file 1 [file molecules-29-04981-s001.zip › molecules-3228021-supplementary.pdf]

Supporting Information

for

Chemical Synthesis of 6-Azido-6-Deoxy Derivatives of Phosphatidylinositol Containing Lipids with Different Fatty Acid Chains

Mosidur Rahaman Molla†, Palak Gupta†, Rajendra Rohokale, and Zhongwu Guo*

Department of Chemistry, University of Florida, 214 Leigh Hall, Gainesville, Florida 32611, United States

*Corresponding author E-mail: zguo@chem.ufl.edu

†These authors contributed equally to the present work

Table of contents

^{1}H, ^{13}C, ^{31}P NMR and HRMS Spectra of All New Compounds…..……..…………………………………………………………………SI-2-50

HPLC Chromatograms of Final Products **1** and **2** and HPLC Conditions ...……………………………………………………………SI-51

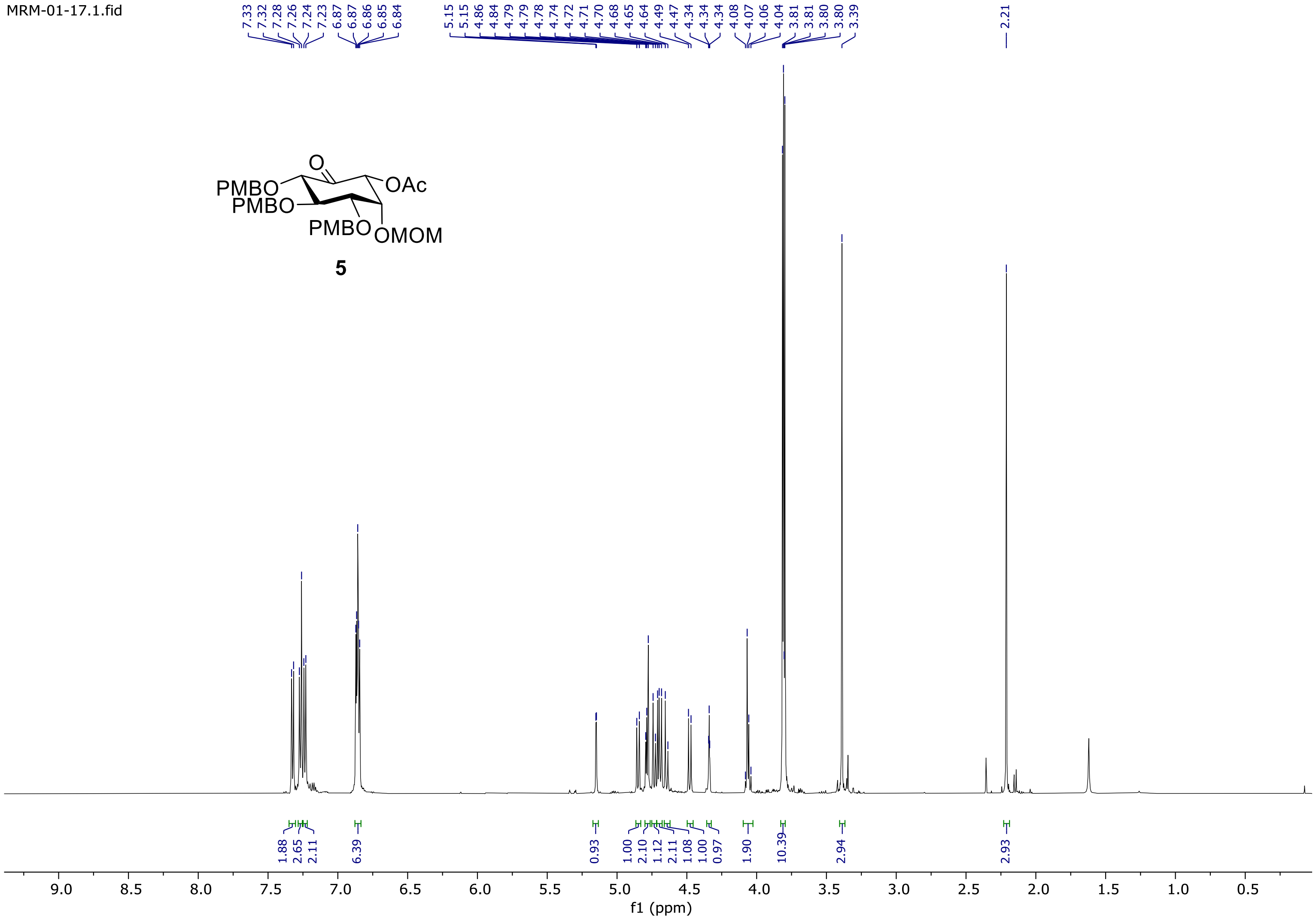

Figure S1: ^{1}H NMR spectrum of compound **5** (600 MHz, $CDCl_3$)

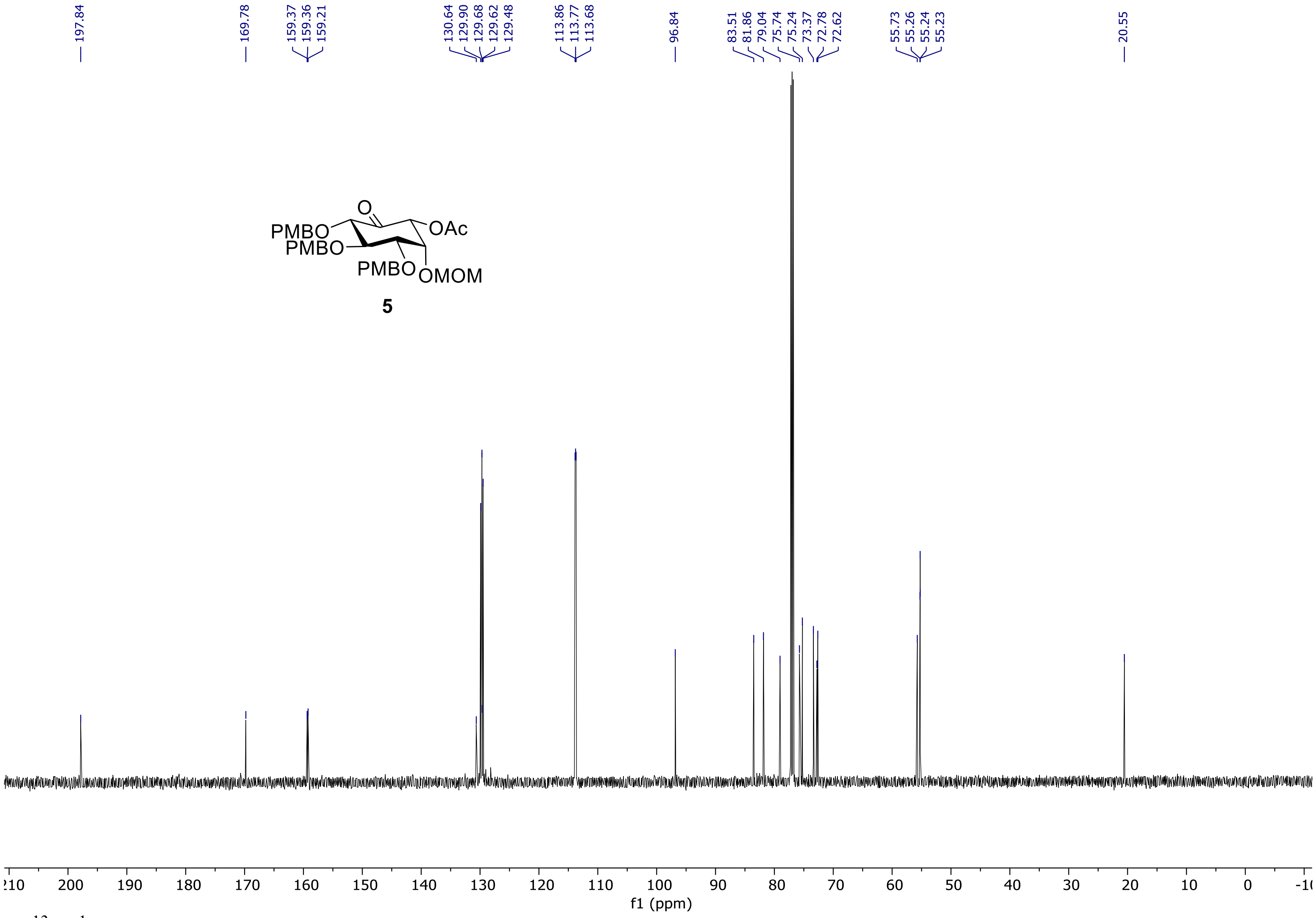

Figure S2: $^{13}C\{^{1}H\}$ NMR spectrum of compound **5** (151 MHz, $CDCl_3$)

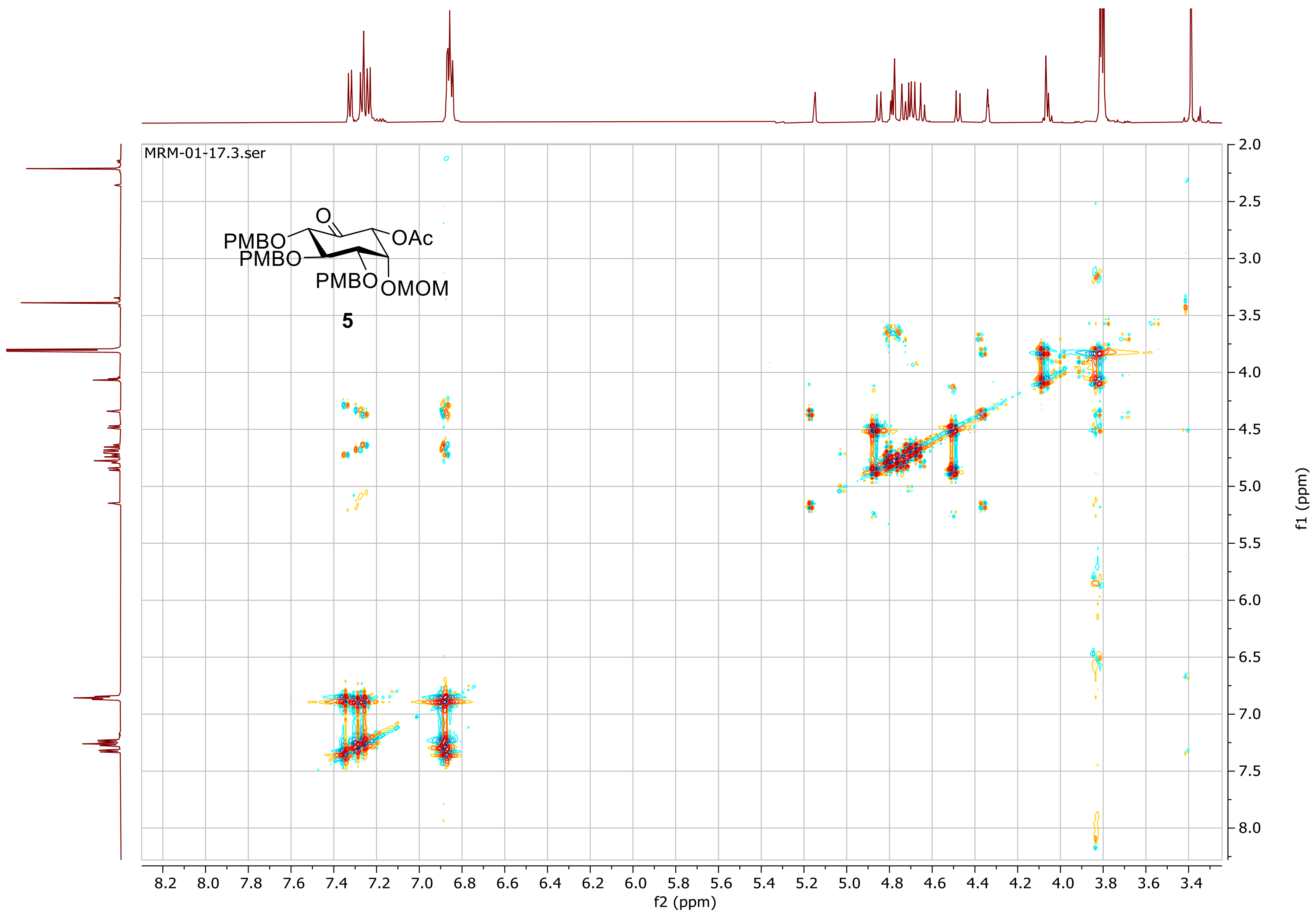

Figure S3: ^{1}H-^{1}H COSY spectrum of compound **5** (600 MHz, $CDCl_3$)

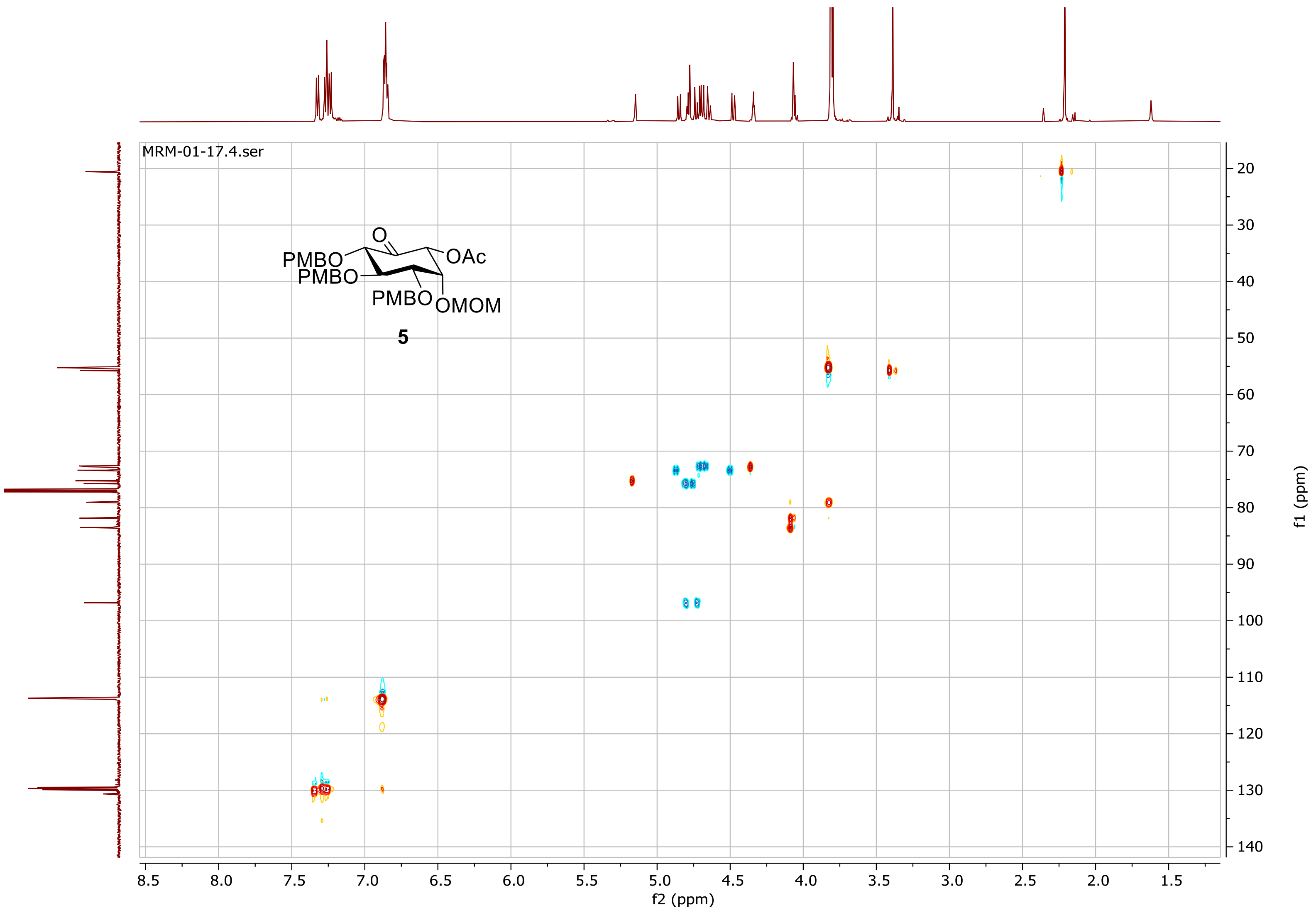

Figure S4: ^{1}H-^{13}C HSQC spectrum of compound **5** (600/151 MHz)

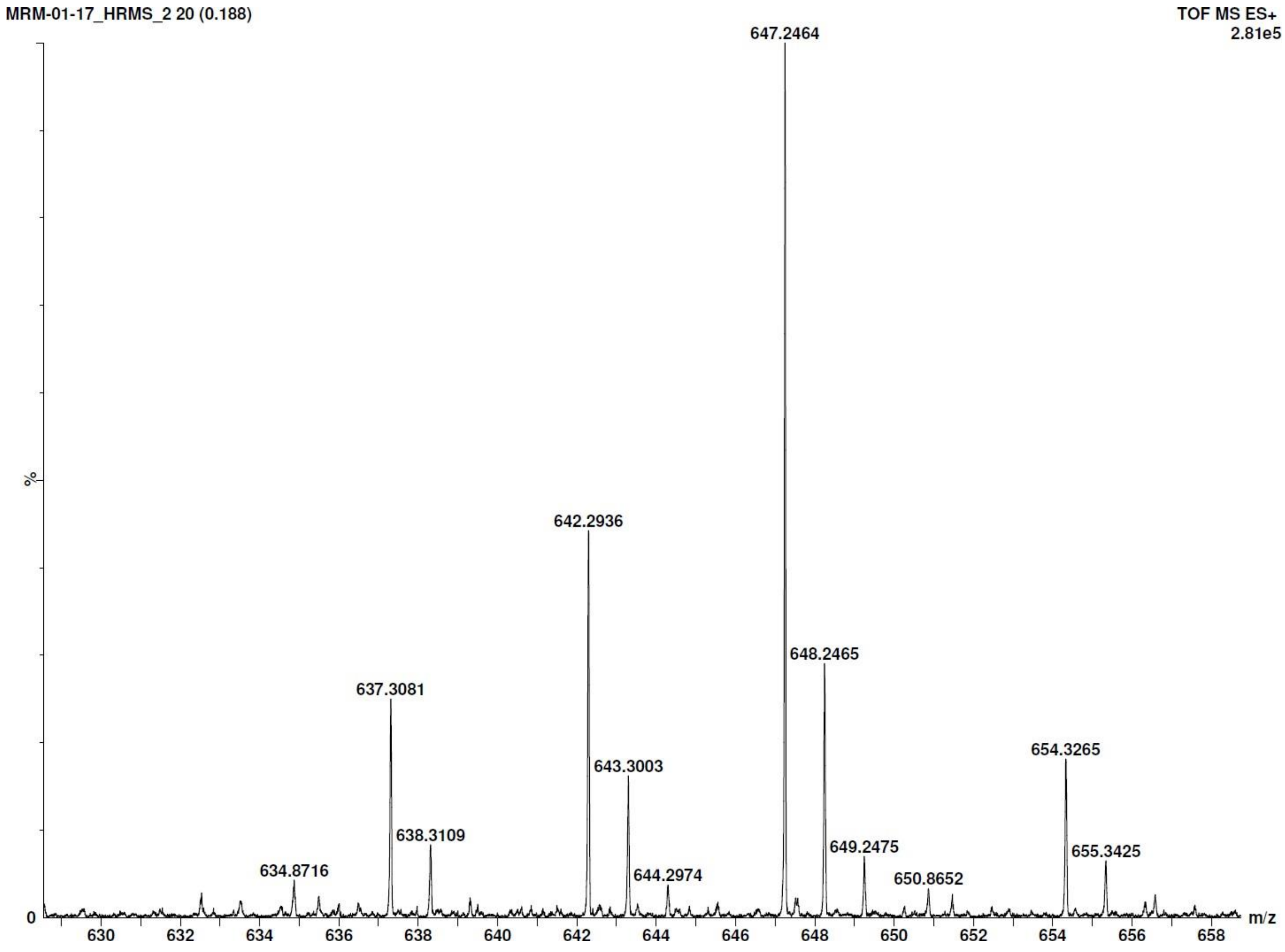

Figure S5: HRMS (ESI- TOF) spectrum of compound **5**

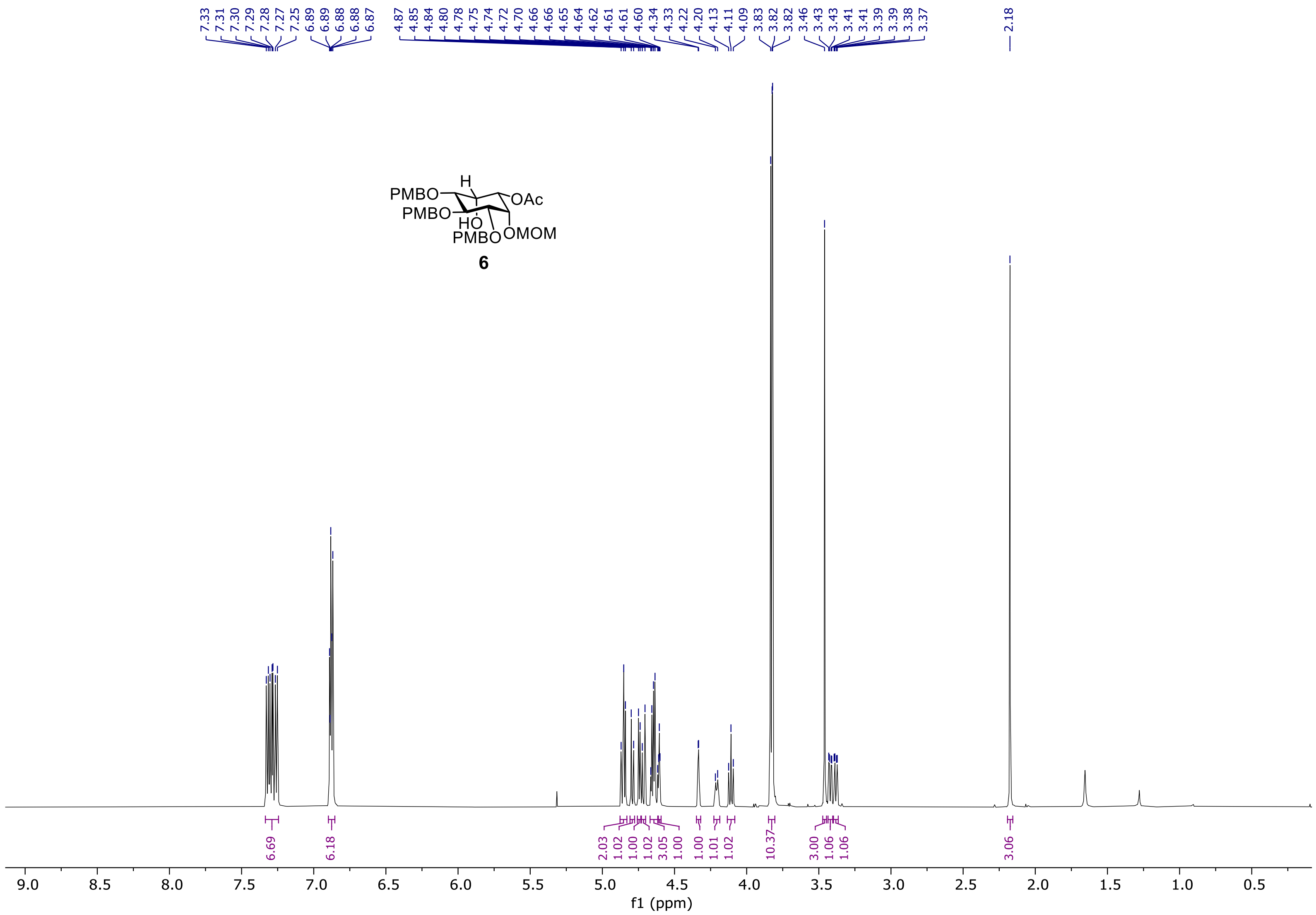

Figure S6: ^{1}H NMR spectrum of compound **6** (600 MHz, $CDCl_3$)

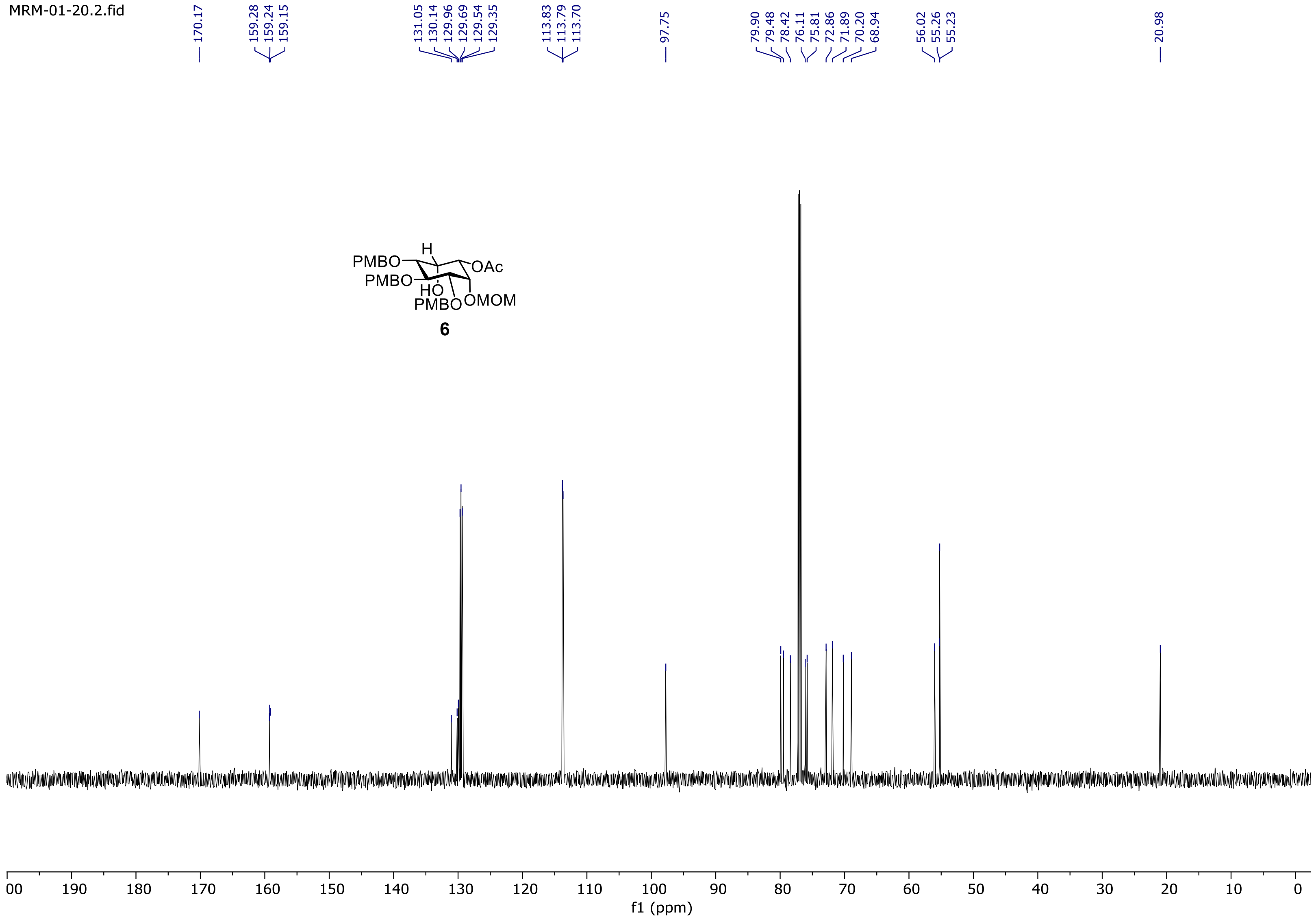

Figure S7: $^{13}C\{^{1}H\}$ NMR spectrum of compound **6** (151 MHz, $CDCl_3$)

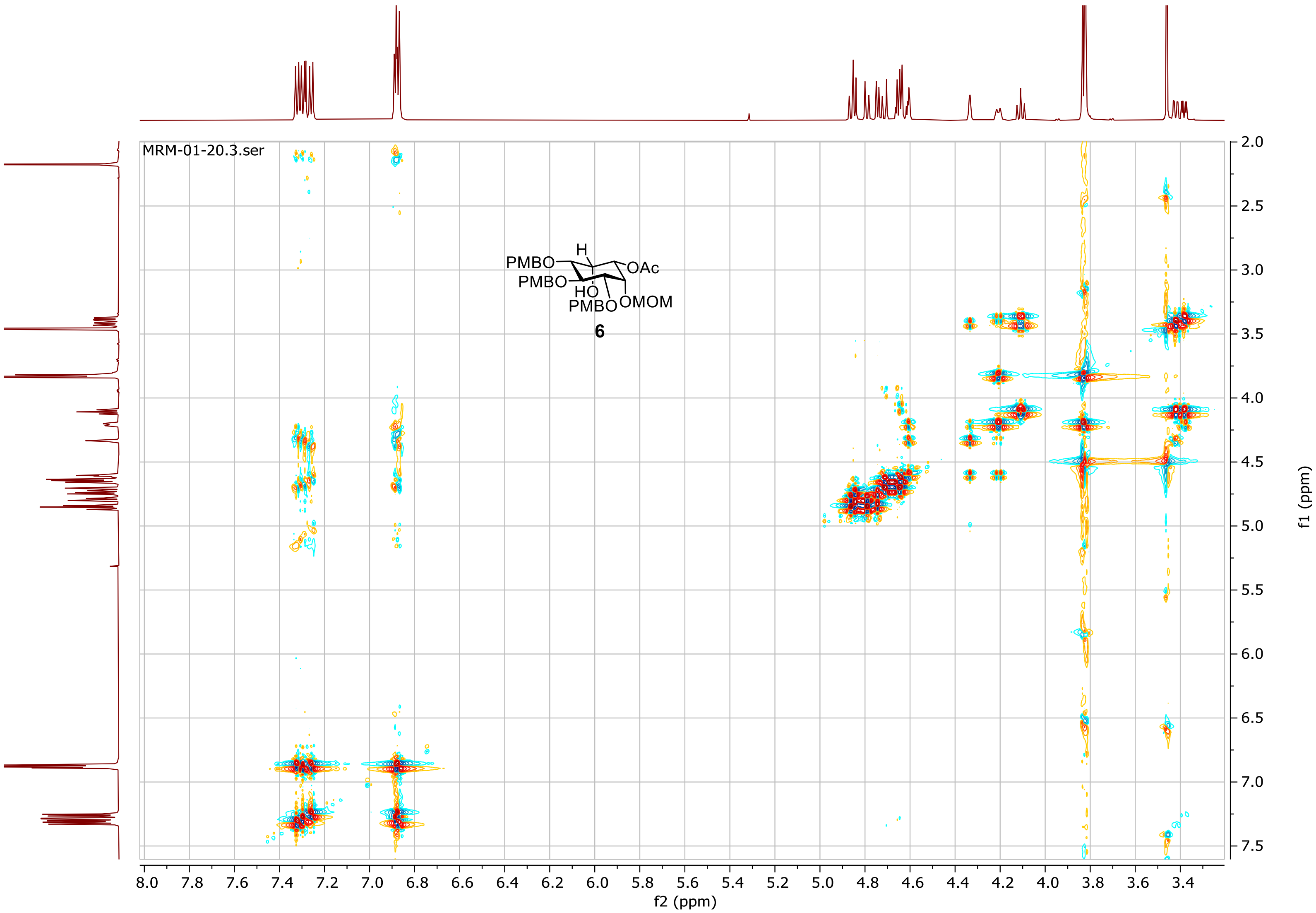

Figure S8: ^{1}H-^{1}H COSY spectrum of compound **6** (600 MHz, $CDCl_3$)

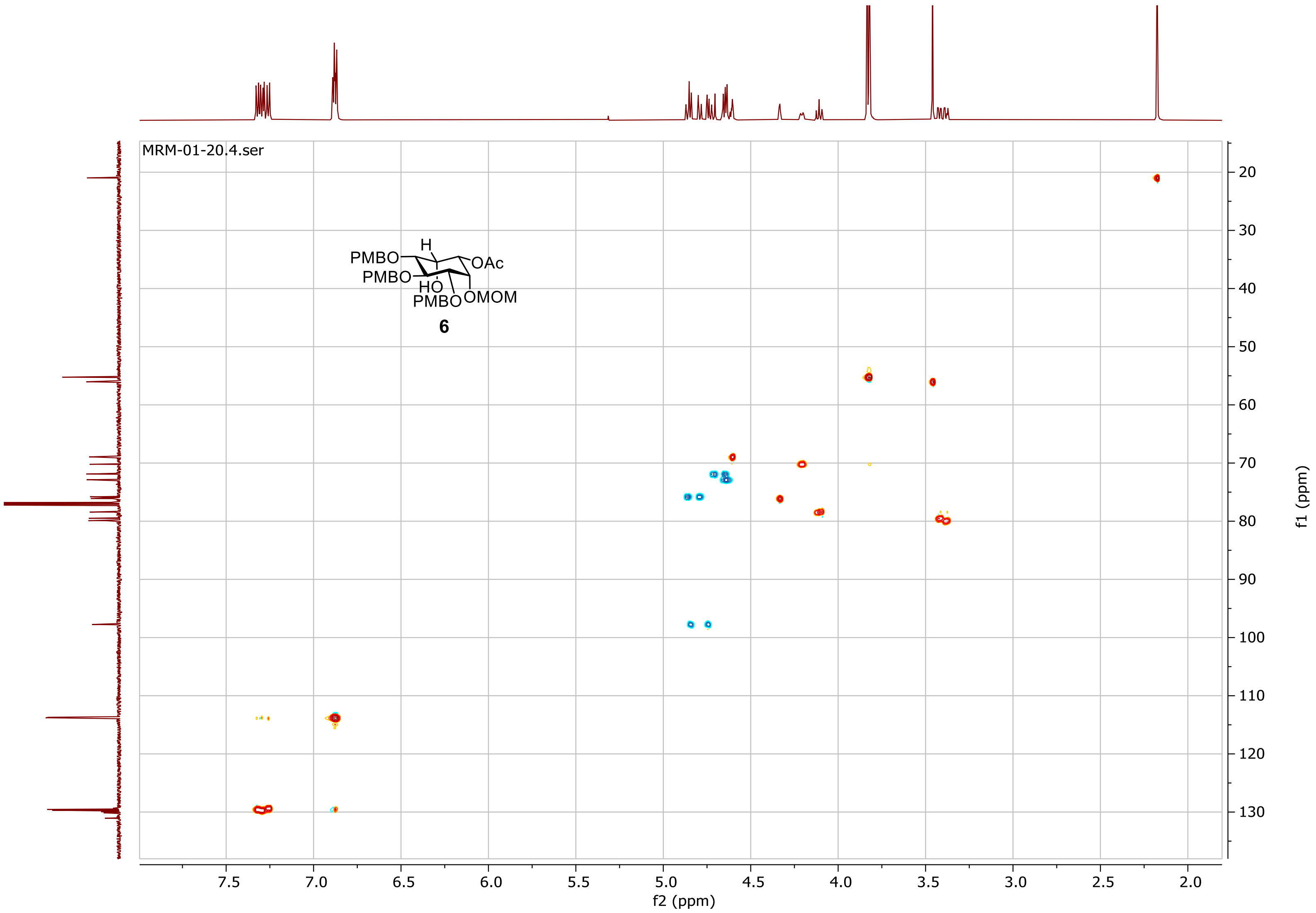

Figure S9: ^{1}H-^{13}C HSQC spectrum of compound **6** (600/151 MHz, $CDCl_3$)

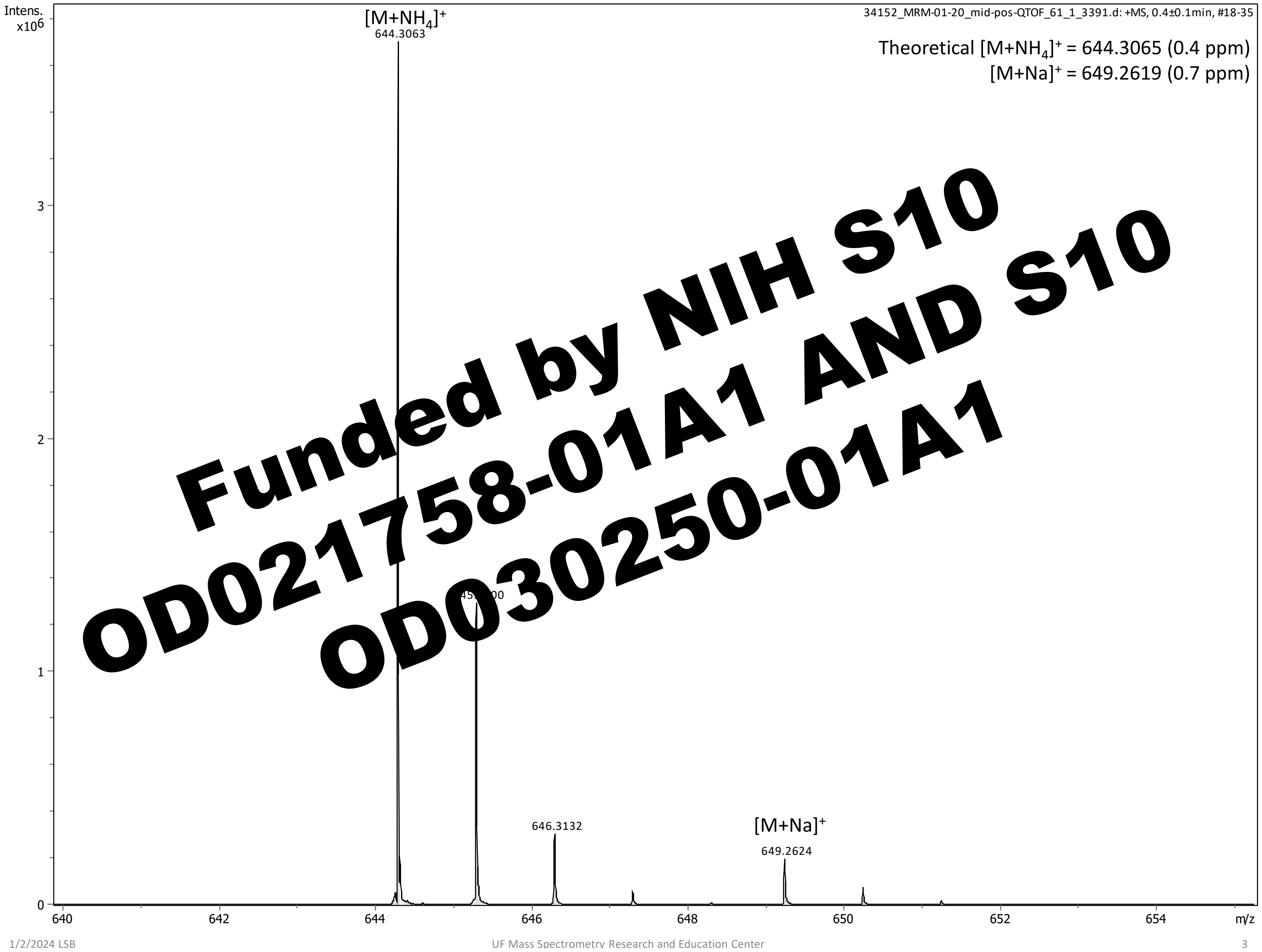

Figure S10: HRMS (ESI- TOF) spectrum of compound **6**

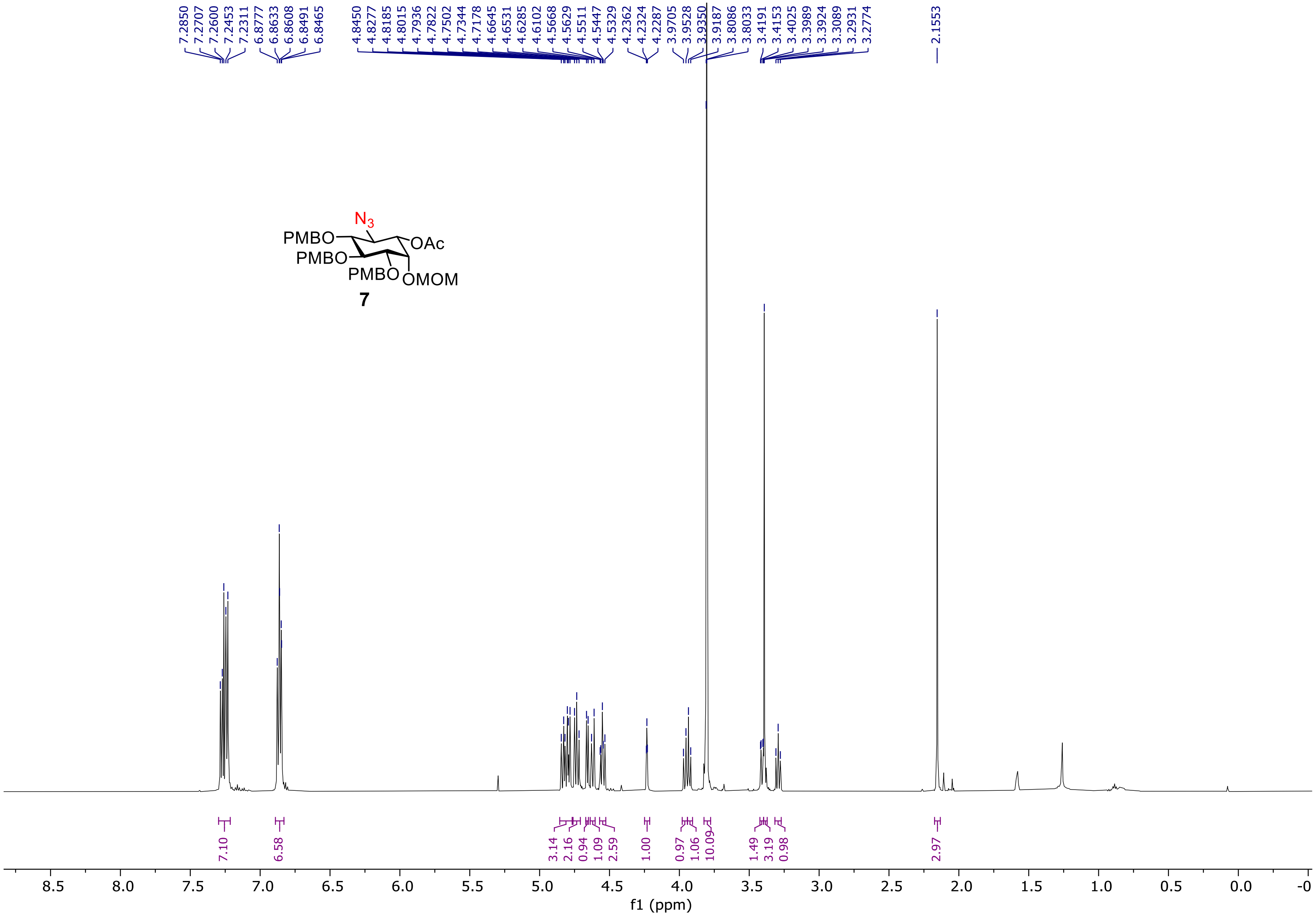

Figure S11: ^{1}H NMR spectrum of compound **7** (600 MHz, $CDCl_3$)

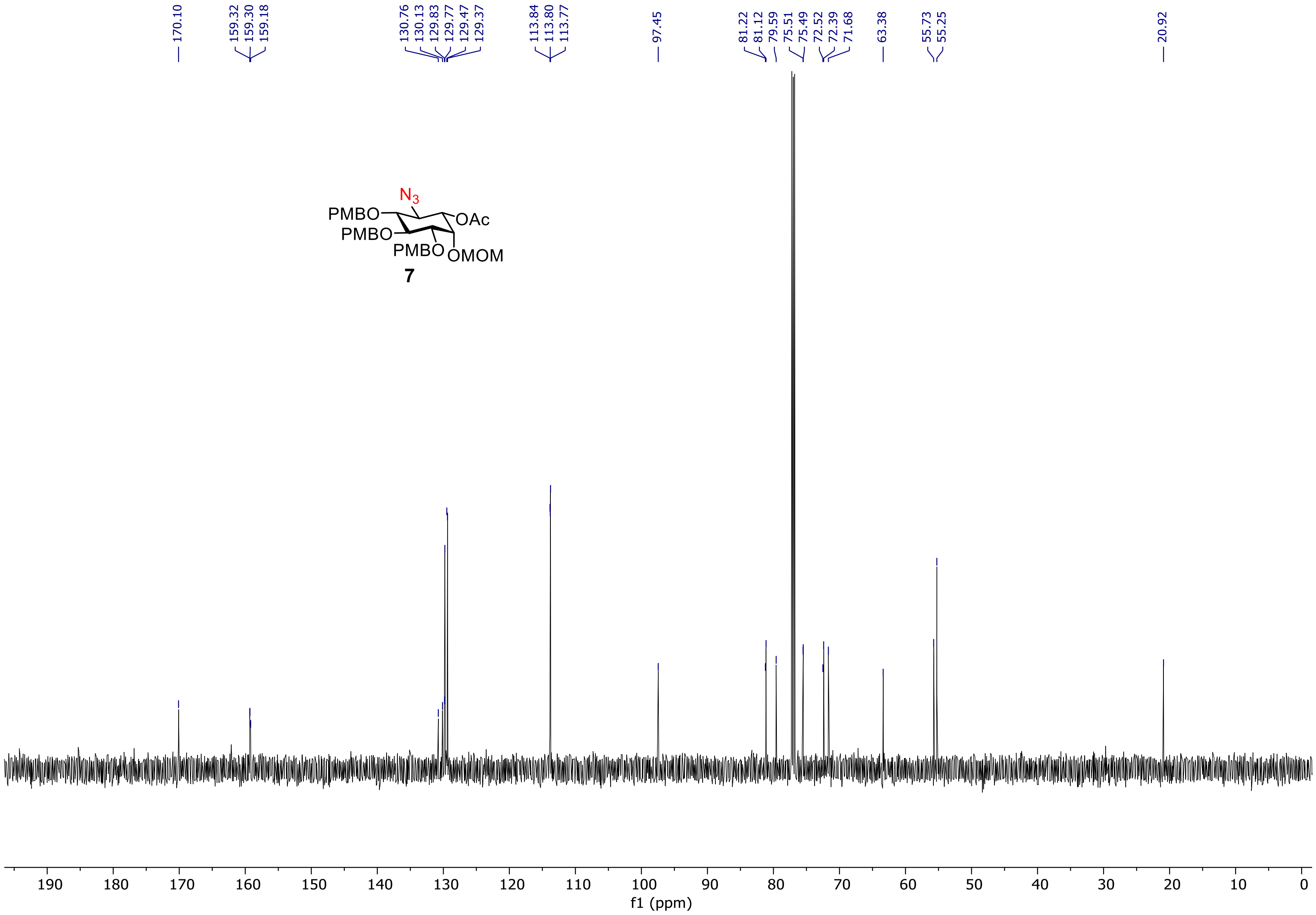

Figure S12: $^{13}C\{^{1}H\}$ NMR spectrum of compound **7** (151 MHz, $CDCl_3$)

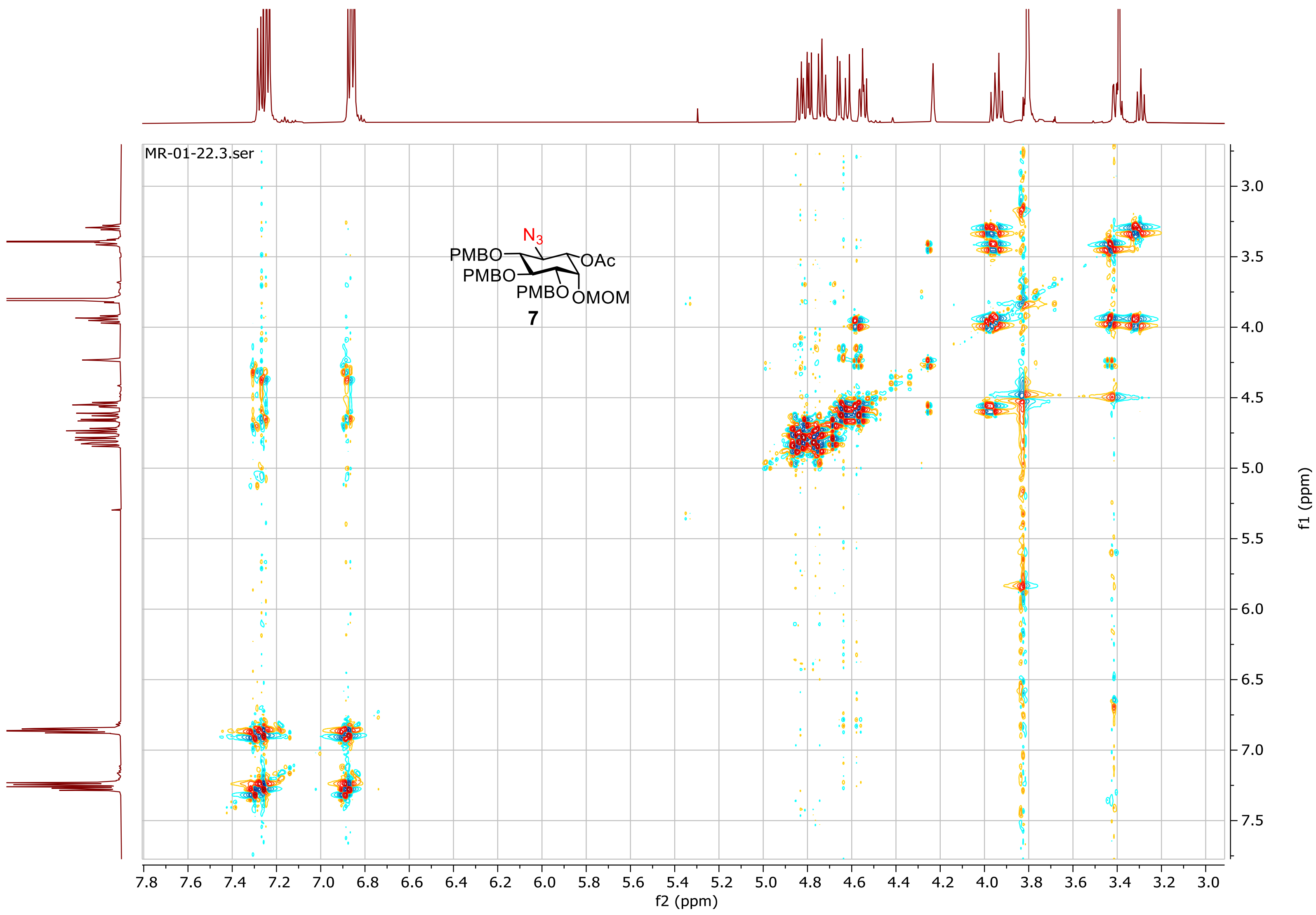

Figure S13: ^{1}H-^{1}H COSY spectrum of compound **7** (600MHz, $CDCl_3$)

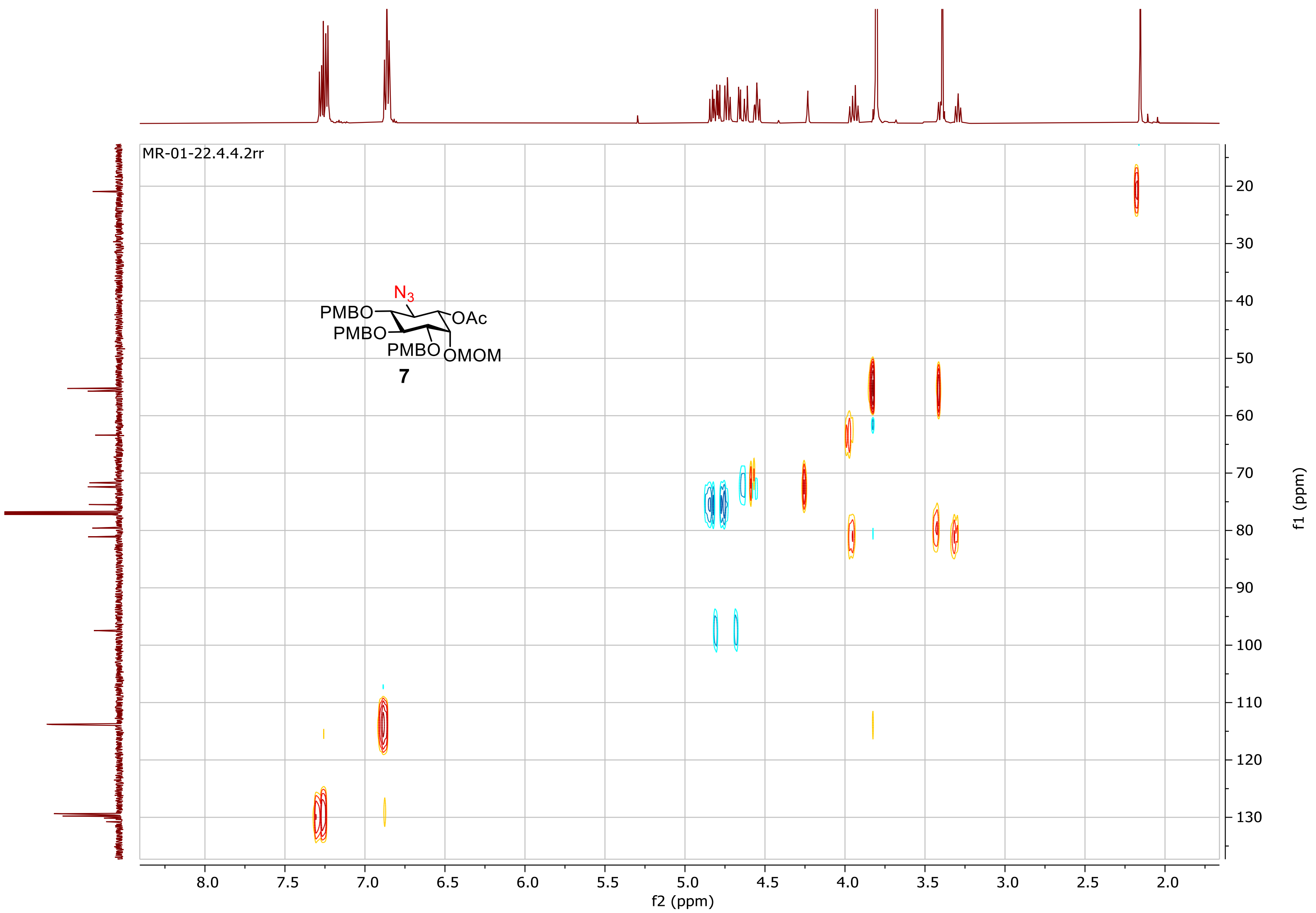

Figure S14: ^{1}H-^{13}C HSQC spectrum of compound **7** (600/151 MHz, $CDCl_3$)

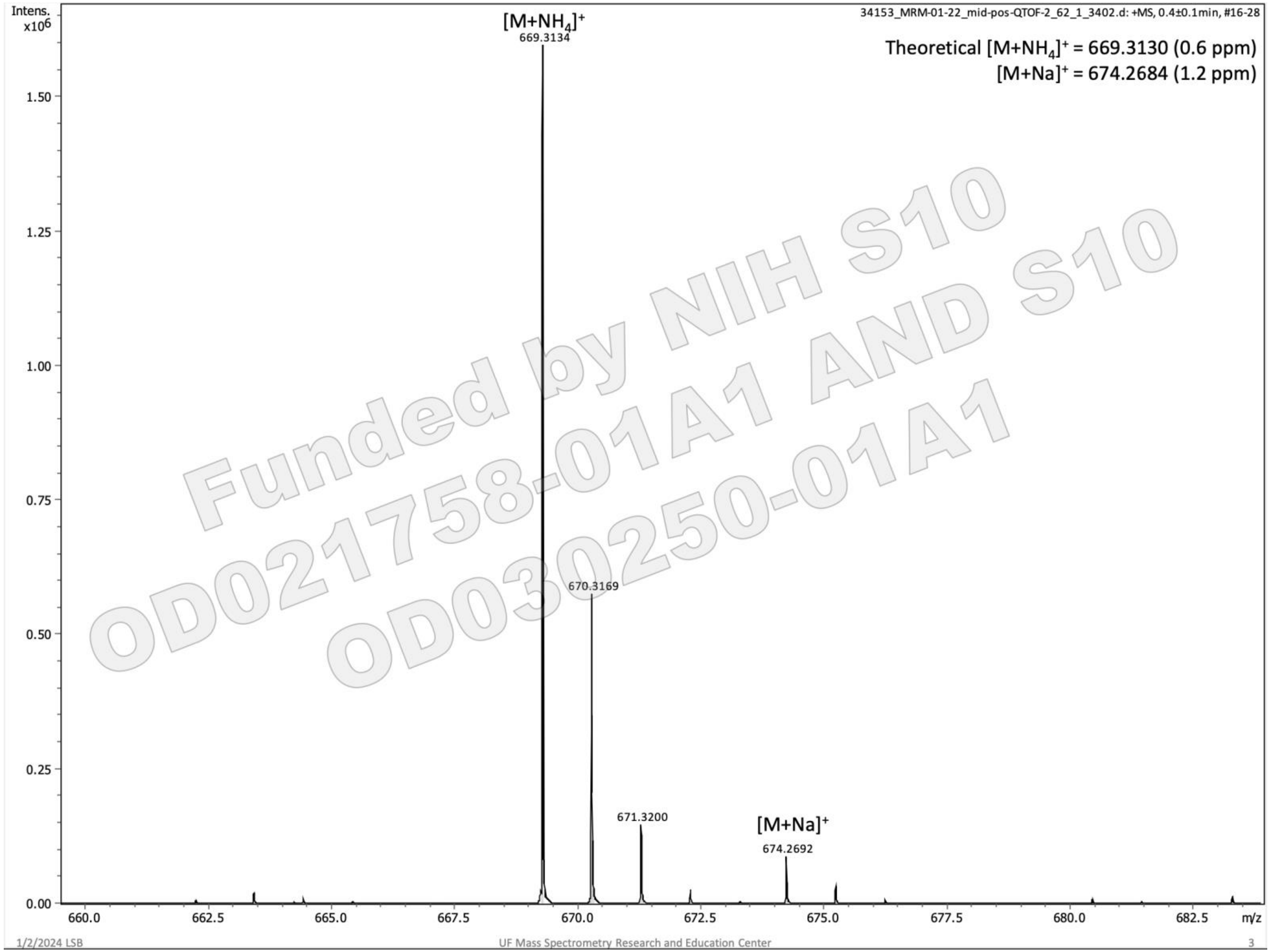

Figure S15: HRMS (ESI- TOF) spectrum of compound **7**

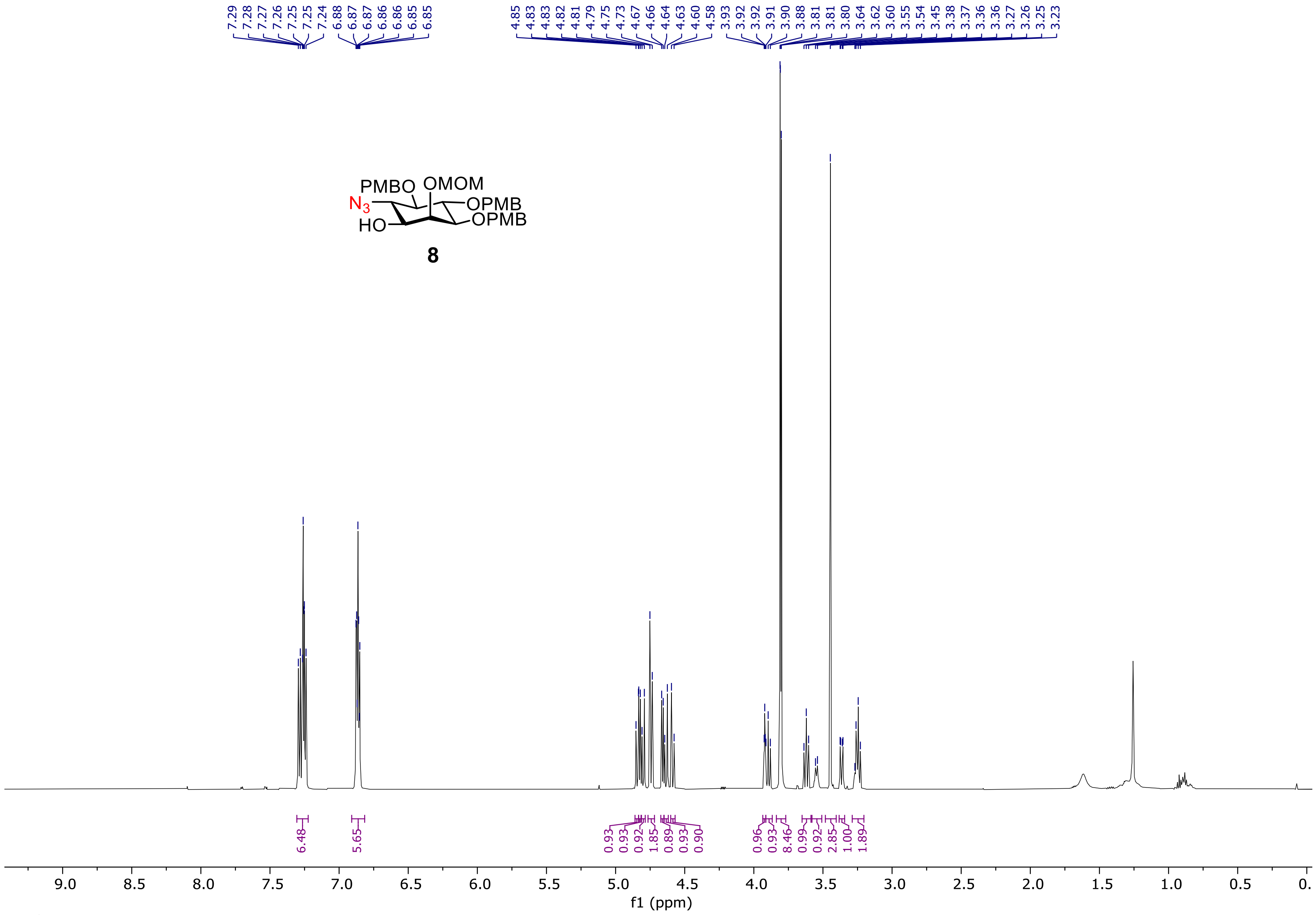

Figure S16: ^{1}H NMR spectrum of compound **8** (600 MHz, $CDCl_3$)

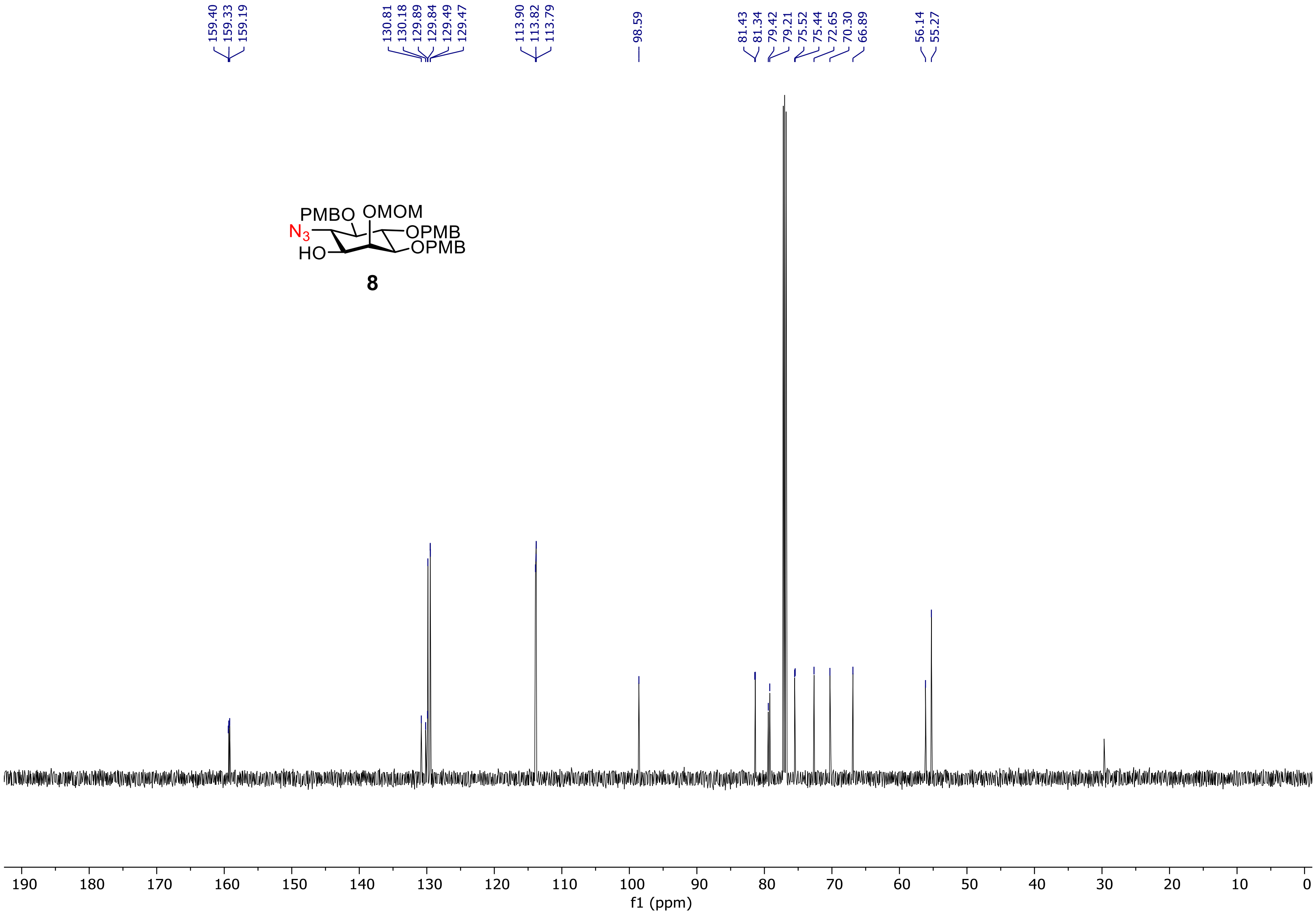

Figure S17: $^{13}C\{^1H\}$ NMR spectrum of compound **8** (151 MHz, $CDCl_3$)

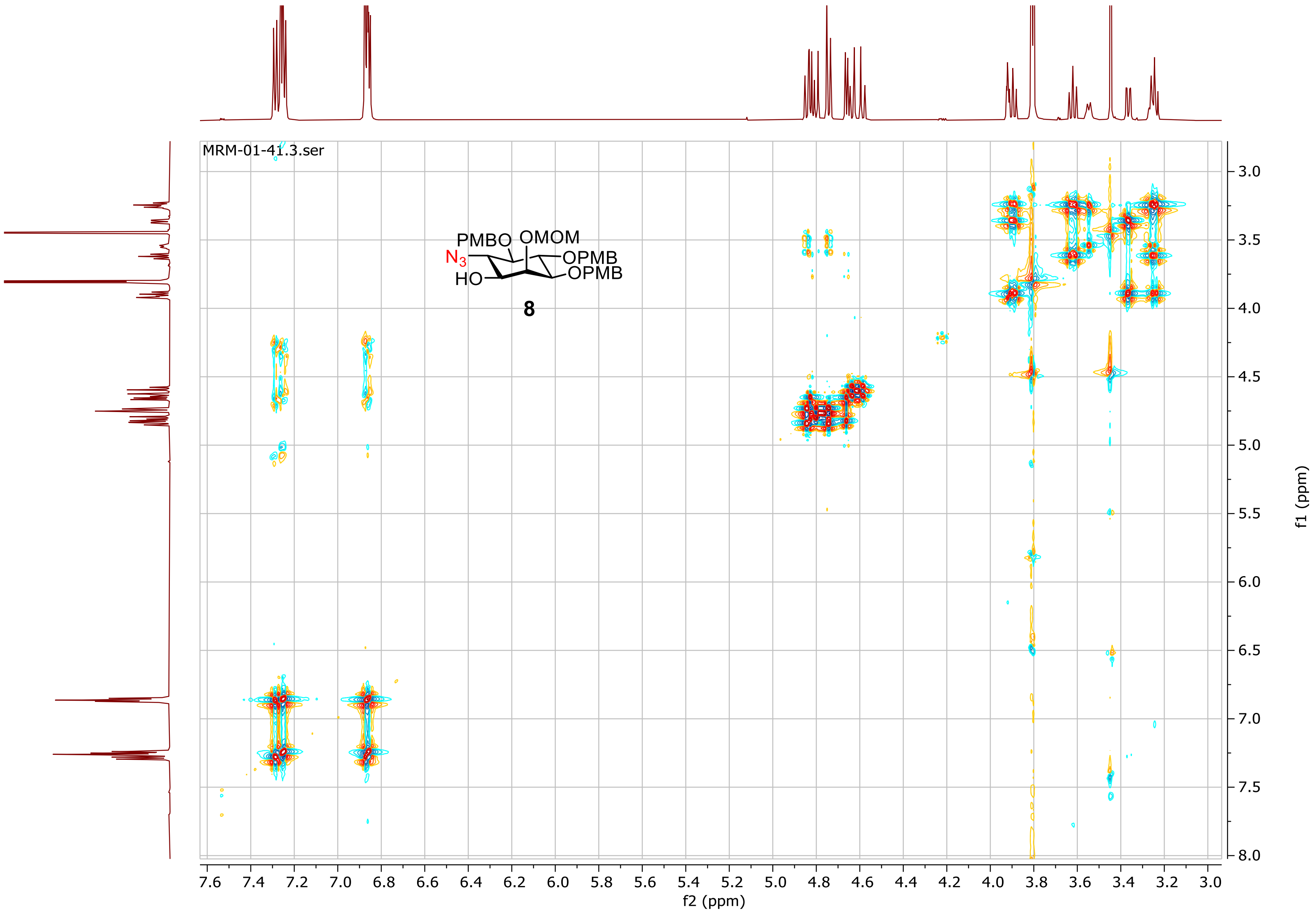

Figure S18: ^{1}H-^{1}H COSY spectrum of compound **8** (600 MHz, $CDCl_3$)

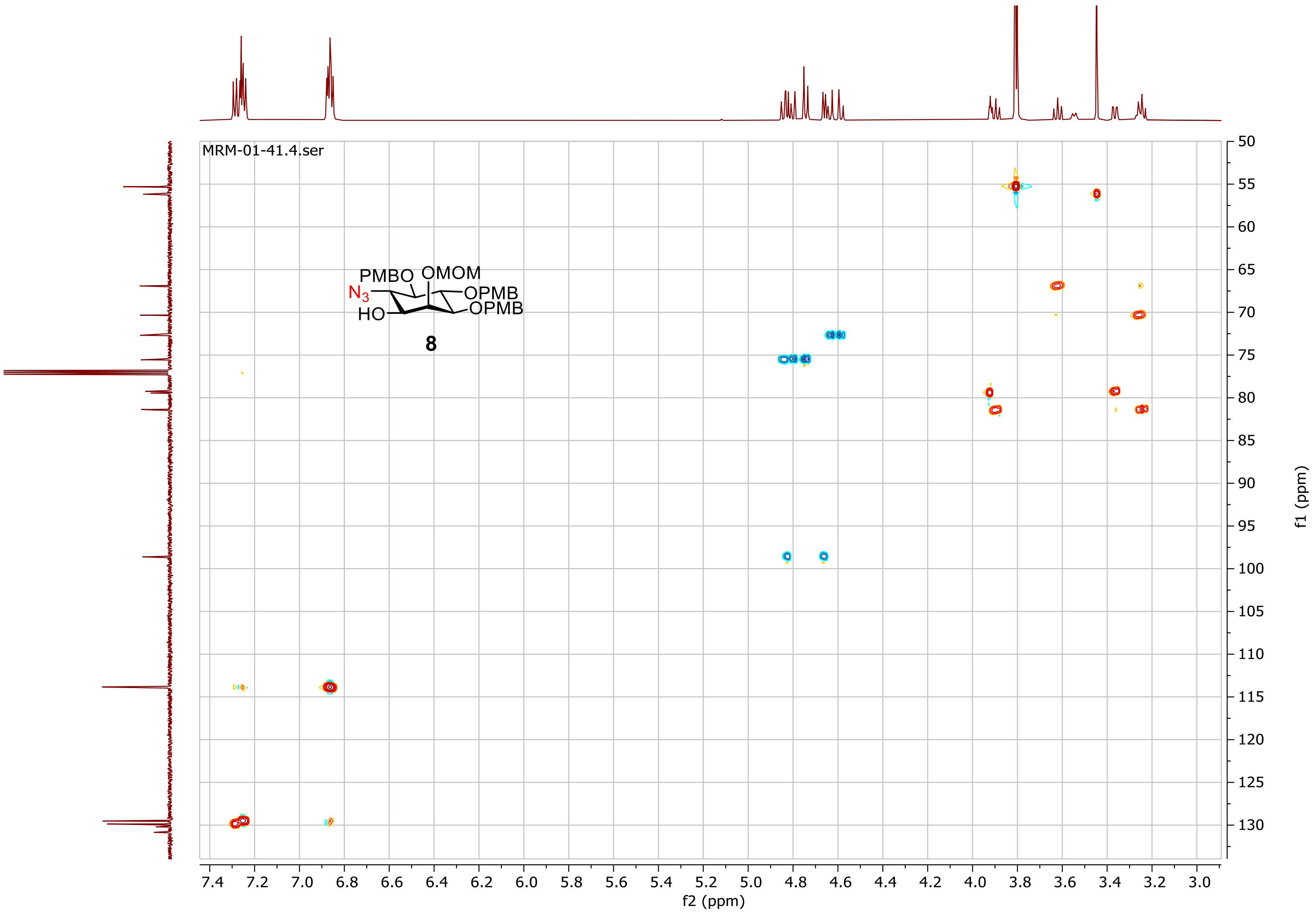

Figure S19: ^{1}H-^{13}C HSQC spectrum of compound **8** (600/151 MHz, $CDCl_3$)

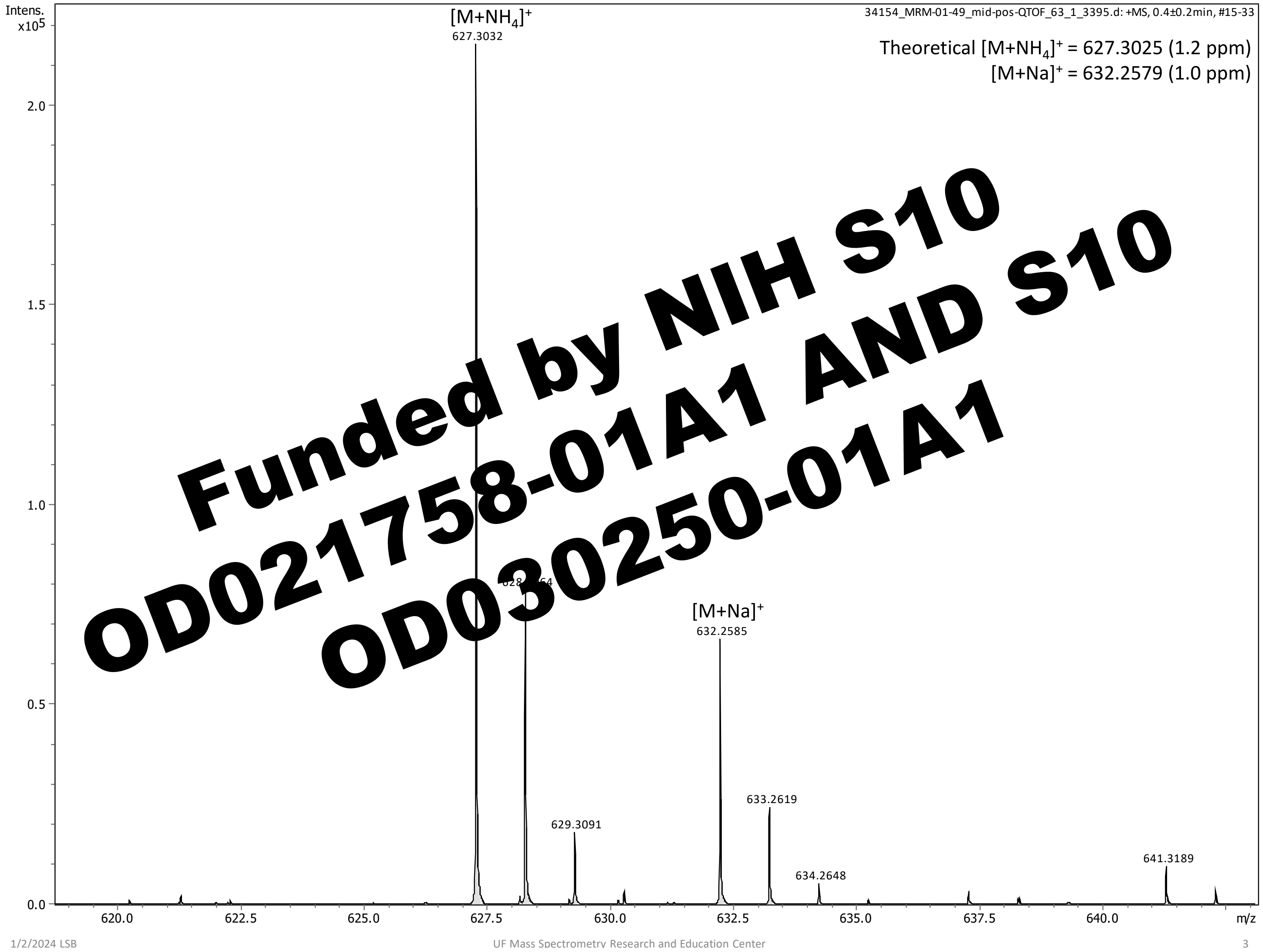

Figure S20: HRMS (ESI- TOF) spectrum of compound **8**

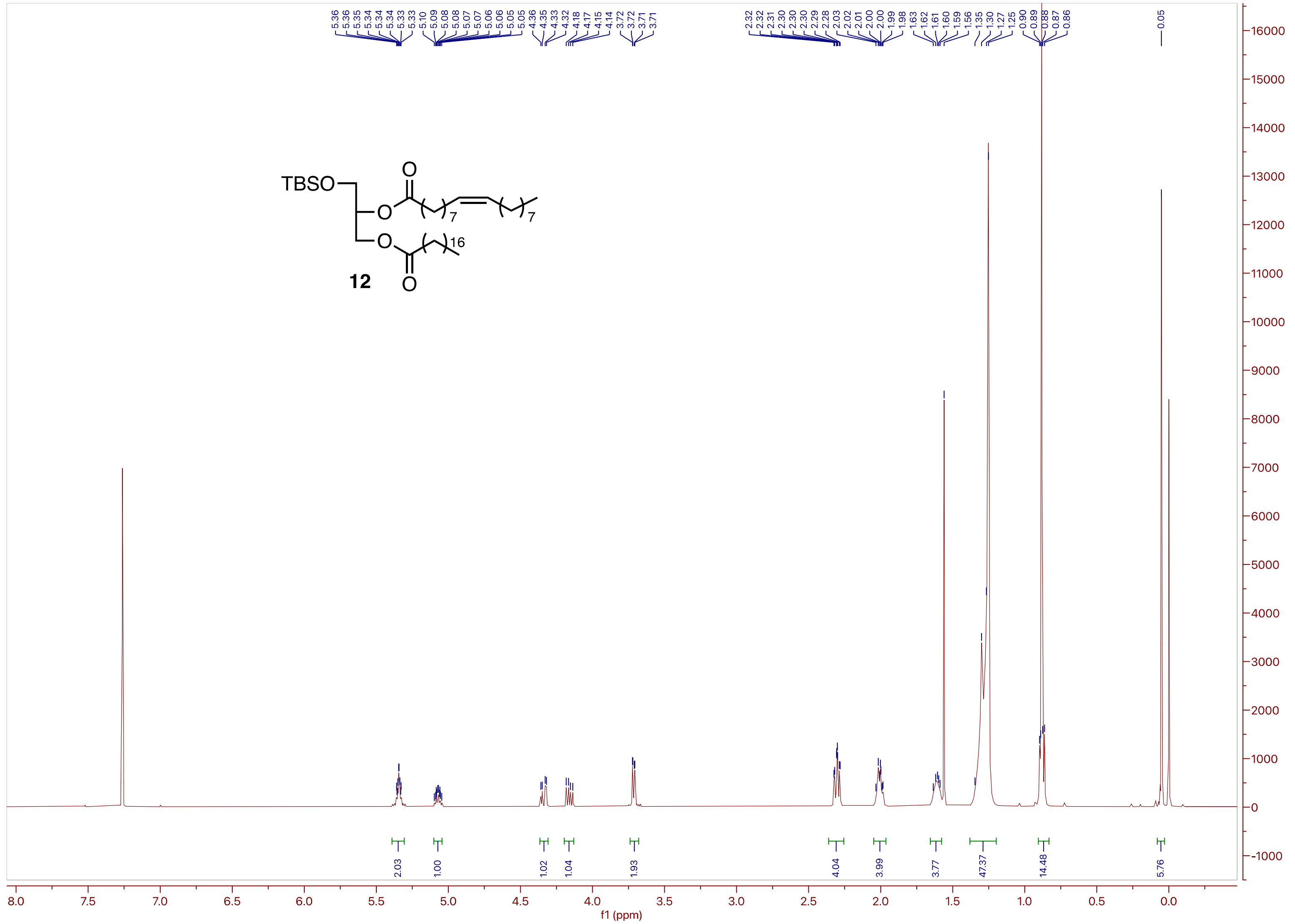

Figure S21. ^{1}H NMR spectrum of compound **12** (400 MHz, $CDCl_3$)

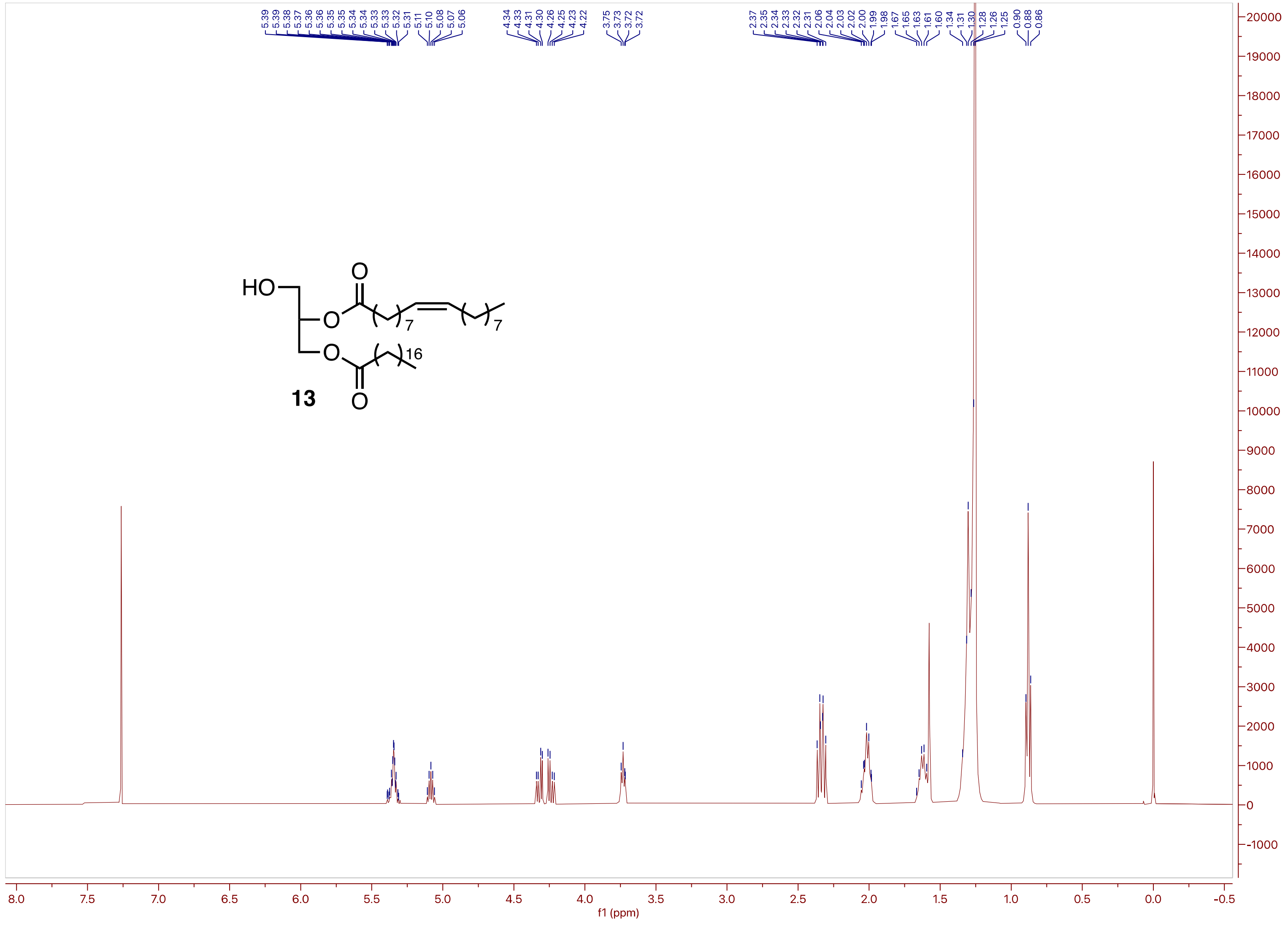

Figure S22. ^{1}H NMR spectrum of compound **13** (400 MHz, $CDCl_3$)

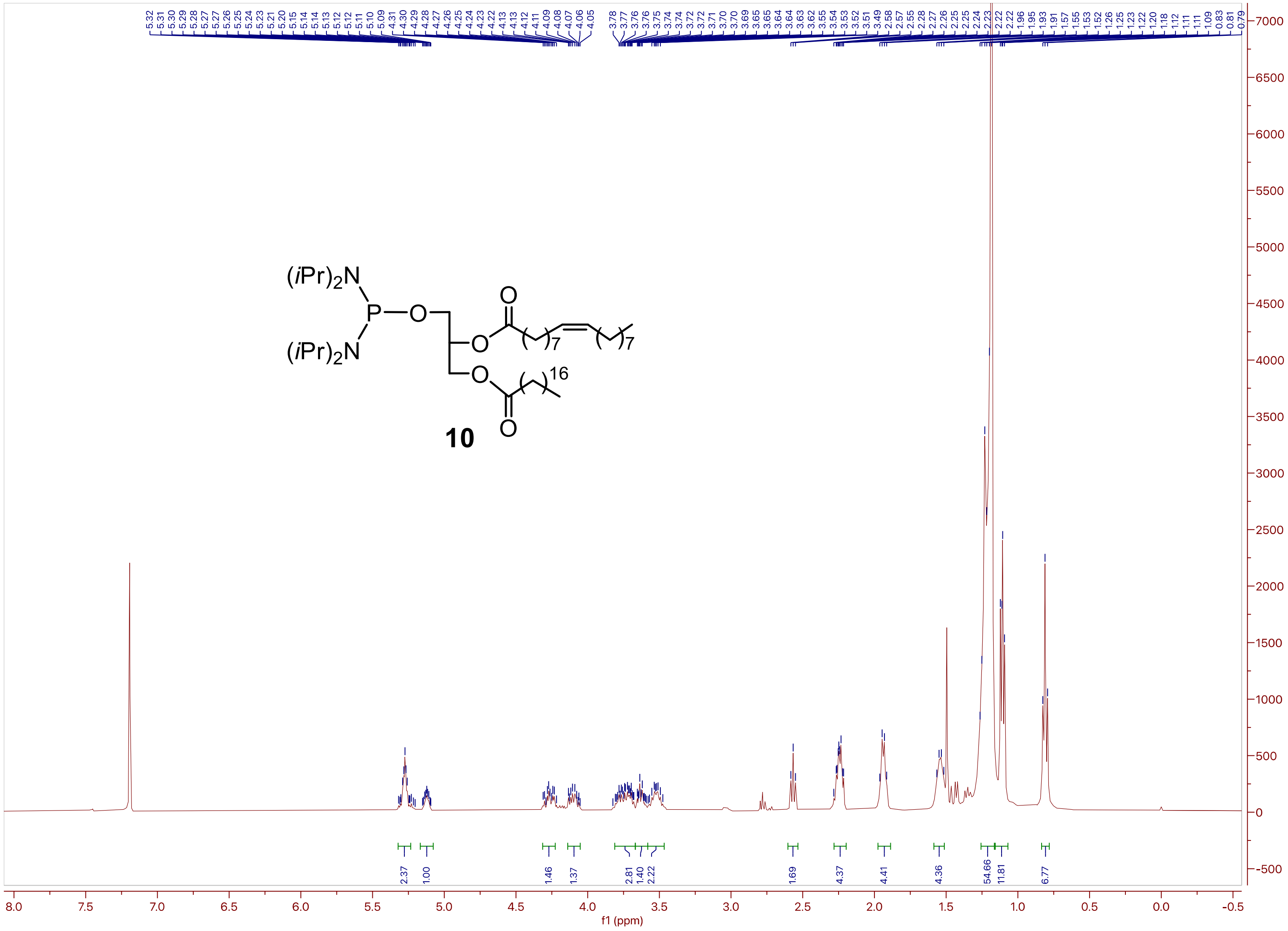

Figure S23. ^{1}H NMR spectrum of compound **10** (400 MHz, $CDCl_3$)

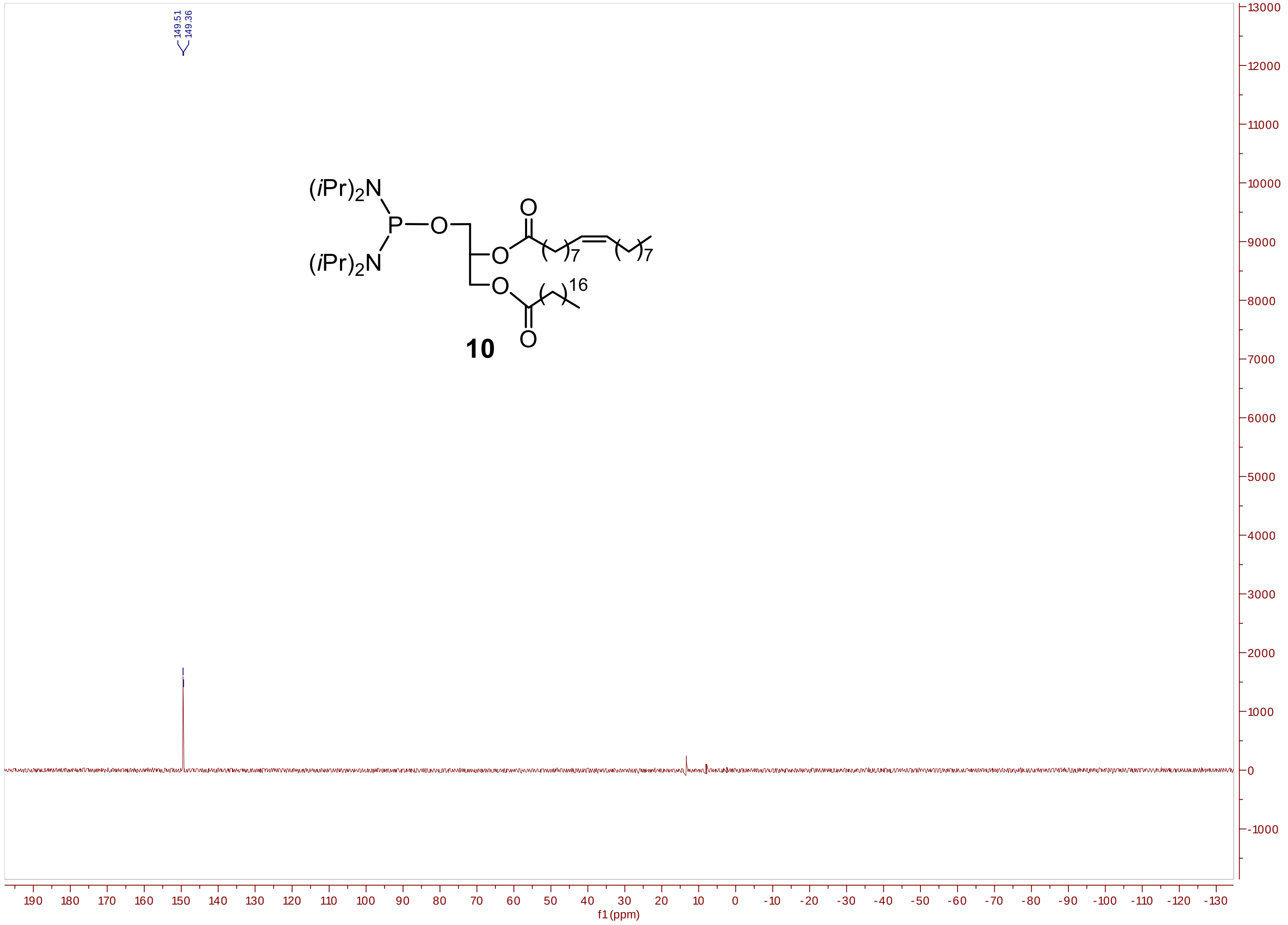

Figure S24. $^{31}P\{^{1}H\}$ NMR spectrum of compound **10** (162 MHz, $CDCl_3$)

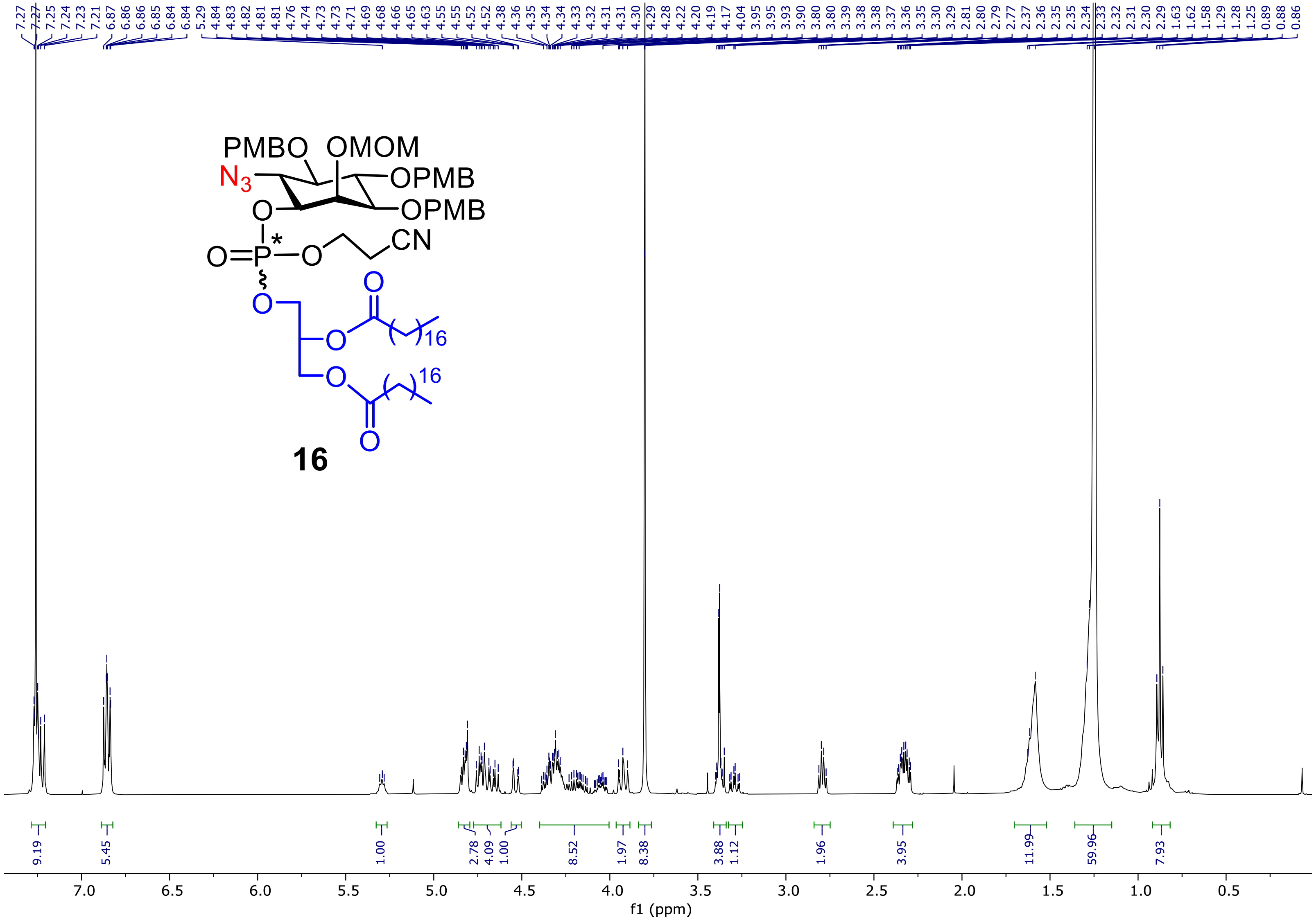

Figure S25: ^{1}H NMR spectrum of compound **16** (400 MHz, $CDCl_3$)

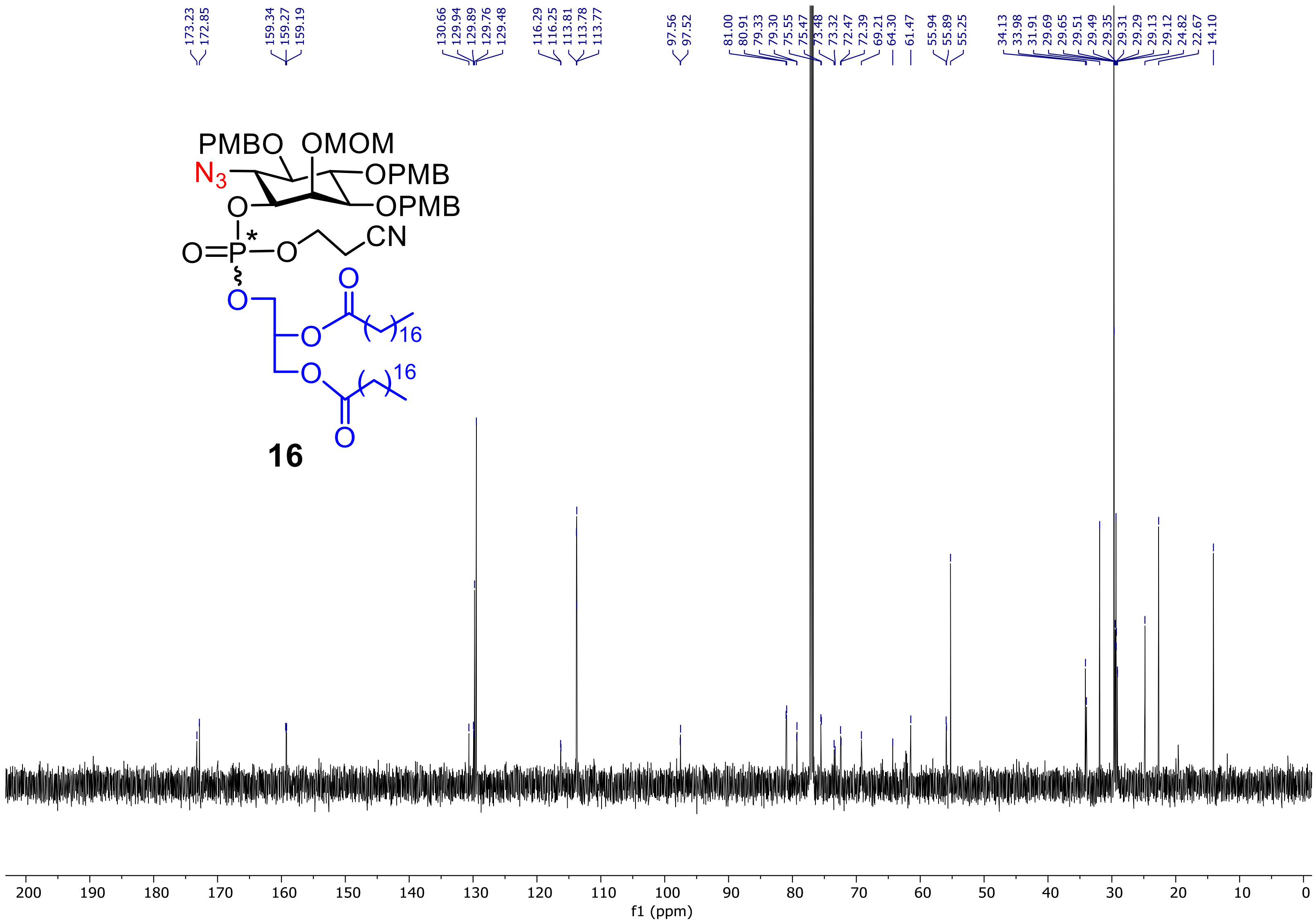

Figure S26: $^{13}C\{^1H\}$ NMR spectrum of compound **16** (100 MHz, $CDCl_3$)

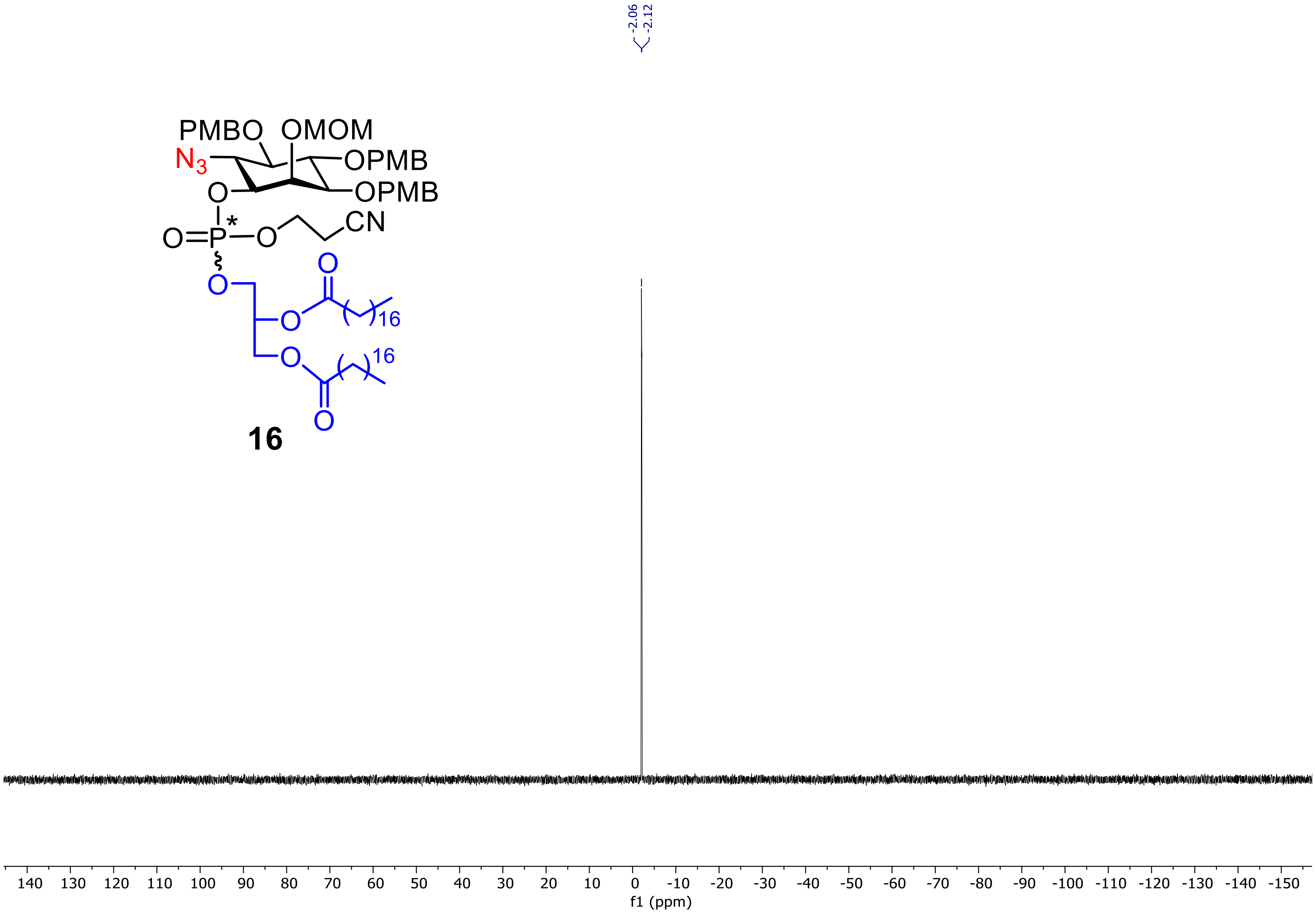

Figure S27: $^{31}P\{^{1}H\}$ NMR spectrum of compound **16** (162 MHz, $CDCl_3$)

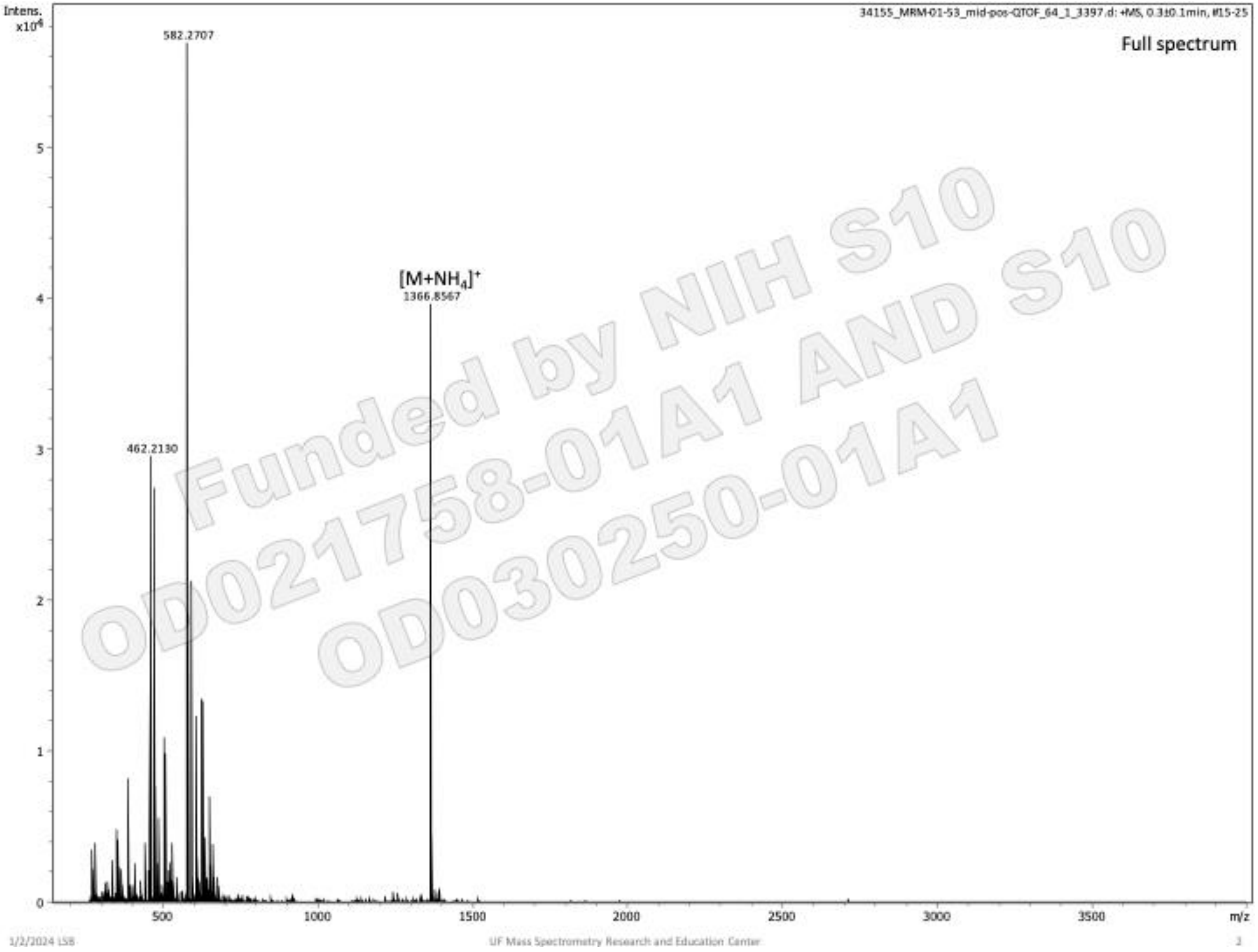

Figure S28: HRMS (ESI- TOF) spectrum of compound **16**

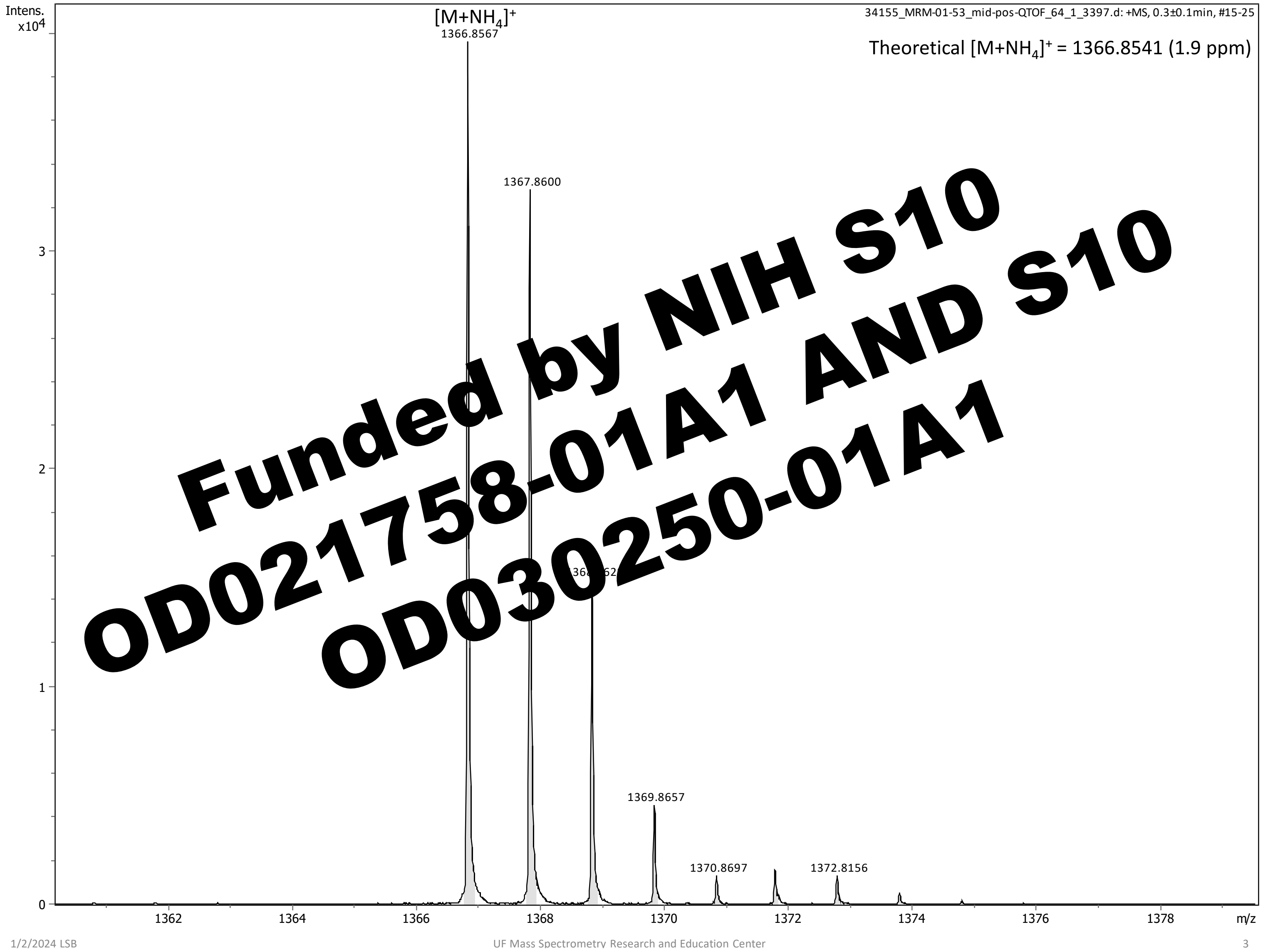

Funded by NIH S10 OD021758-01A1 AND S10 OD030250-01A1

Figure S29: Expanded HRMS (ESI- TOF) spectrum of compound **16**

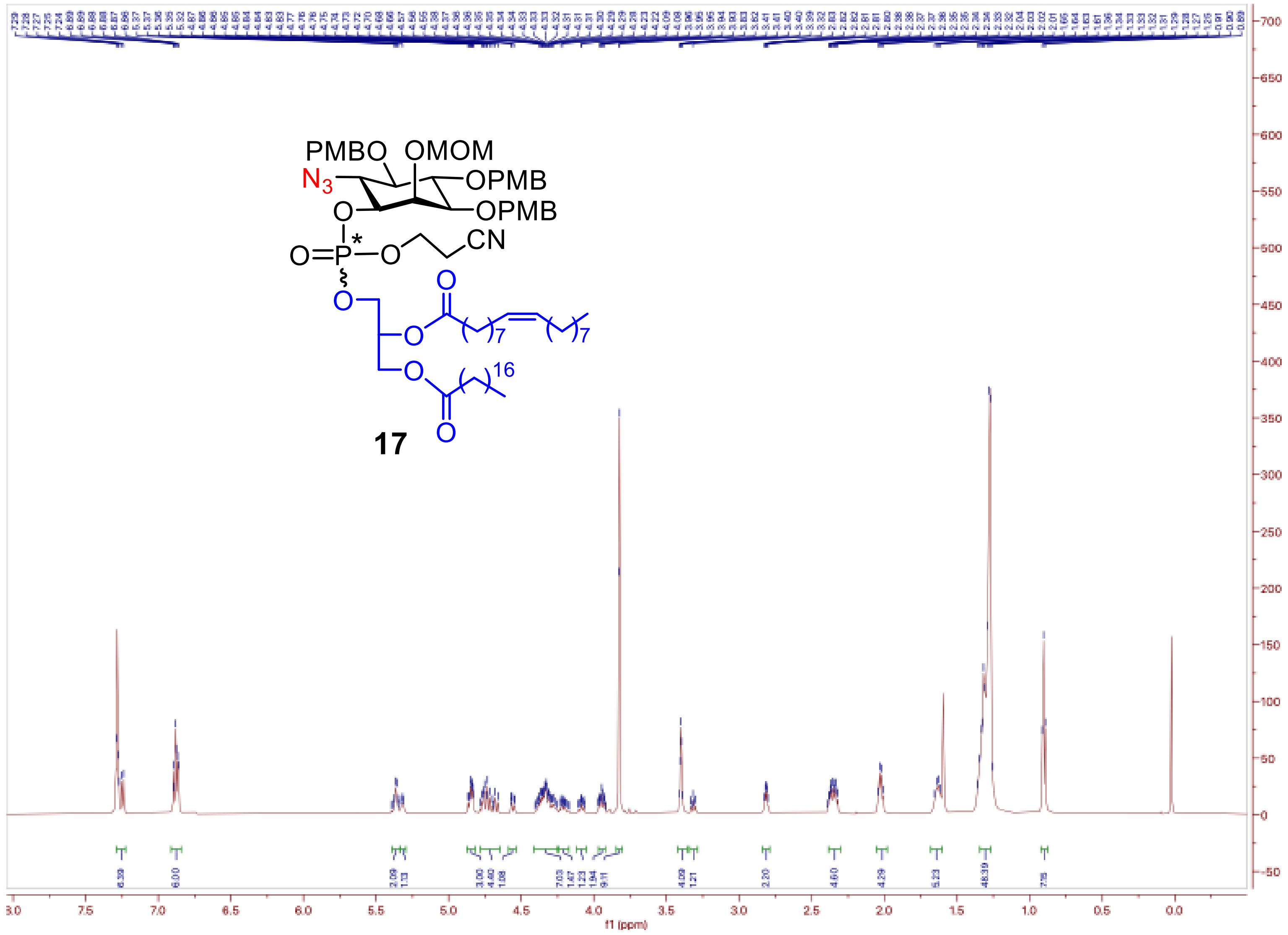

Figure S30. ^{1}H NMR spectrum of compound **17** (600 MHz, $CDCl_3$)

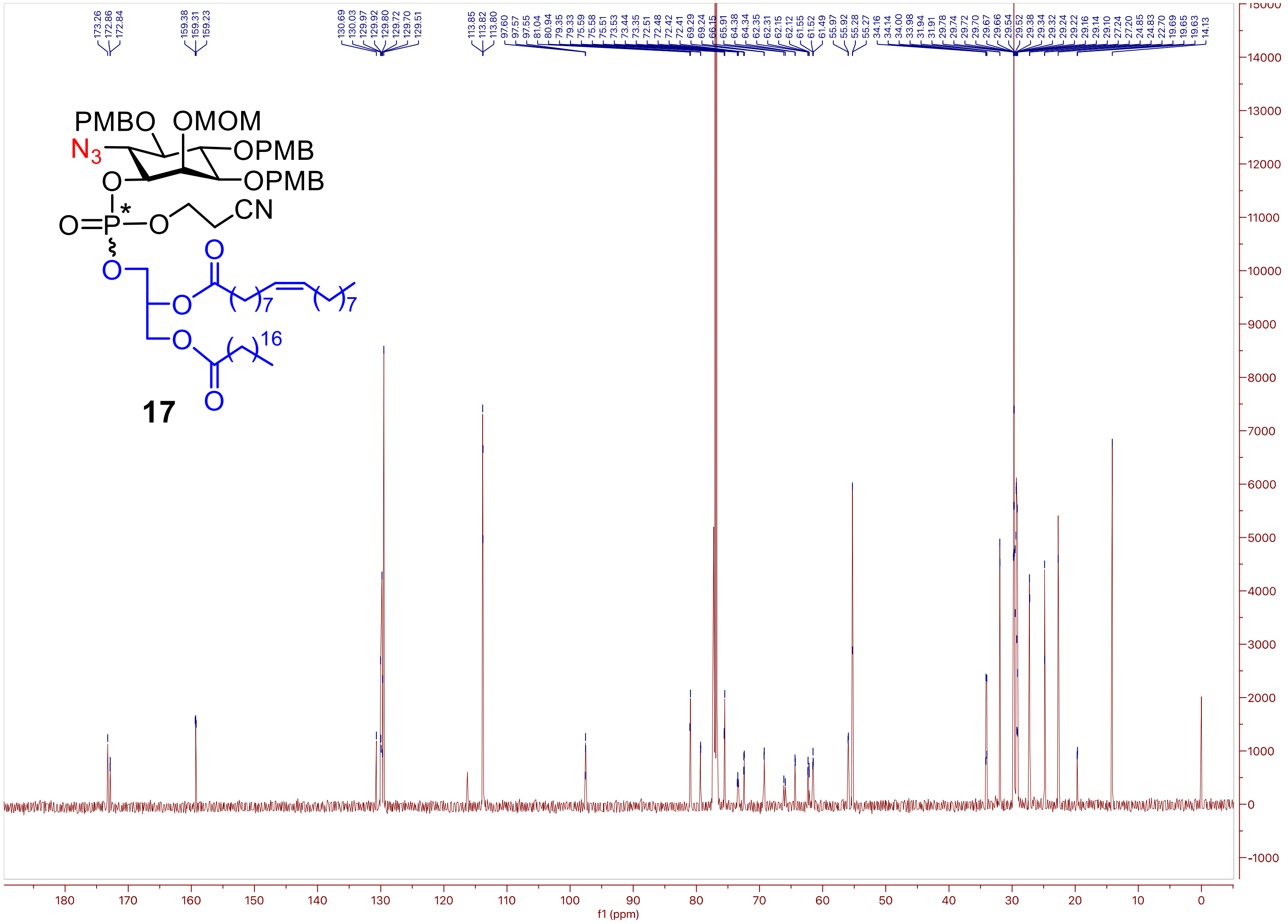

Figure S31. $^{13}C\{^{1}H\}$ NMR spectrum of compound **17** (151 MHz, $CDCl_3$)

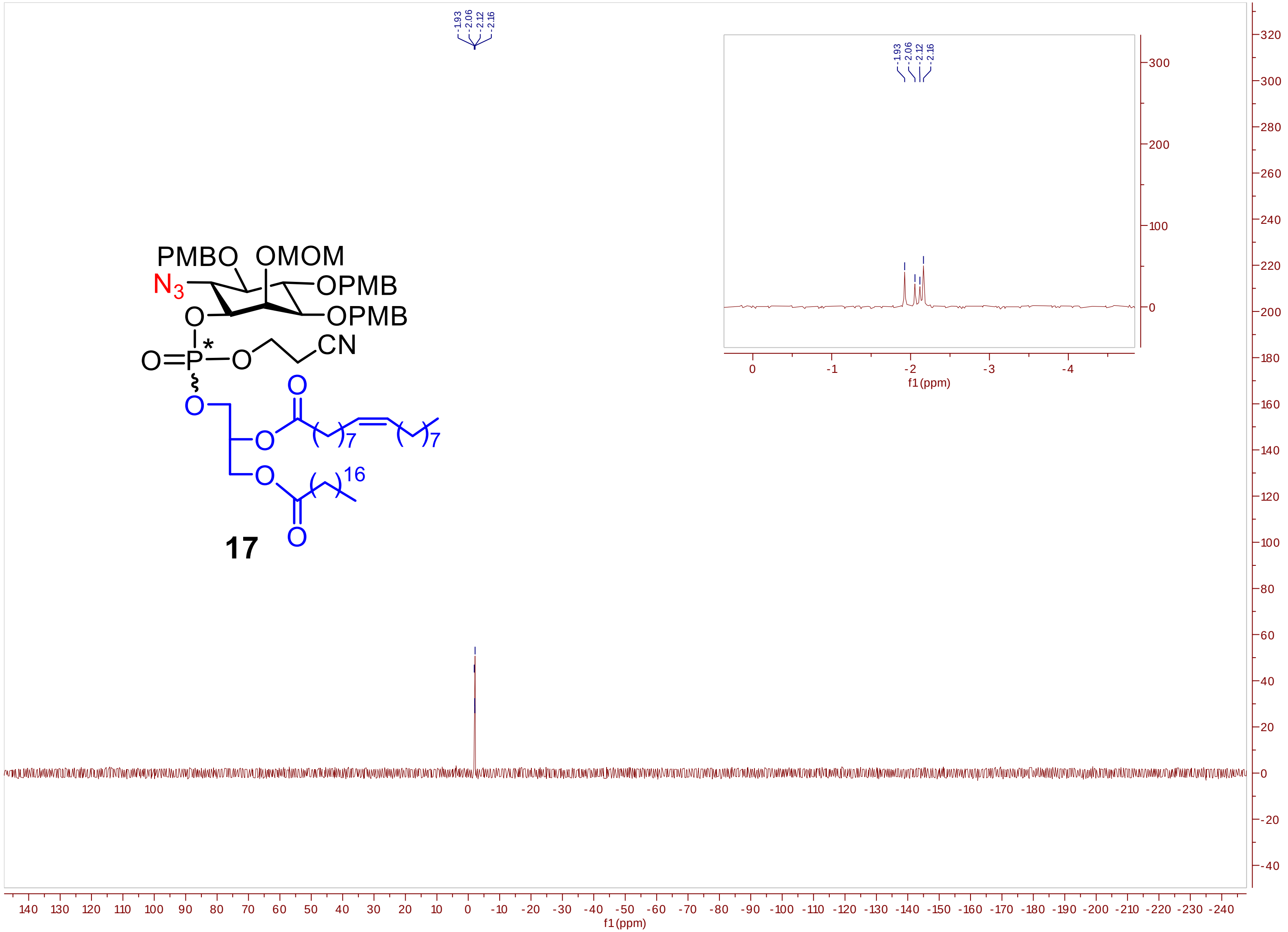

Figure S32. $^{31}P\{^{1}H\}$ NMR spectrum of compound **17** (243 MHz, $CDCl_3$)

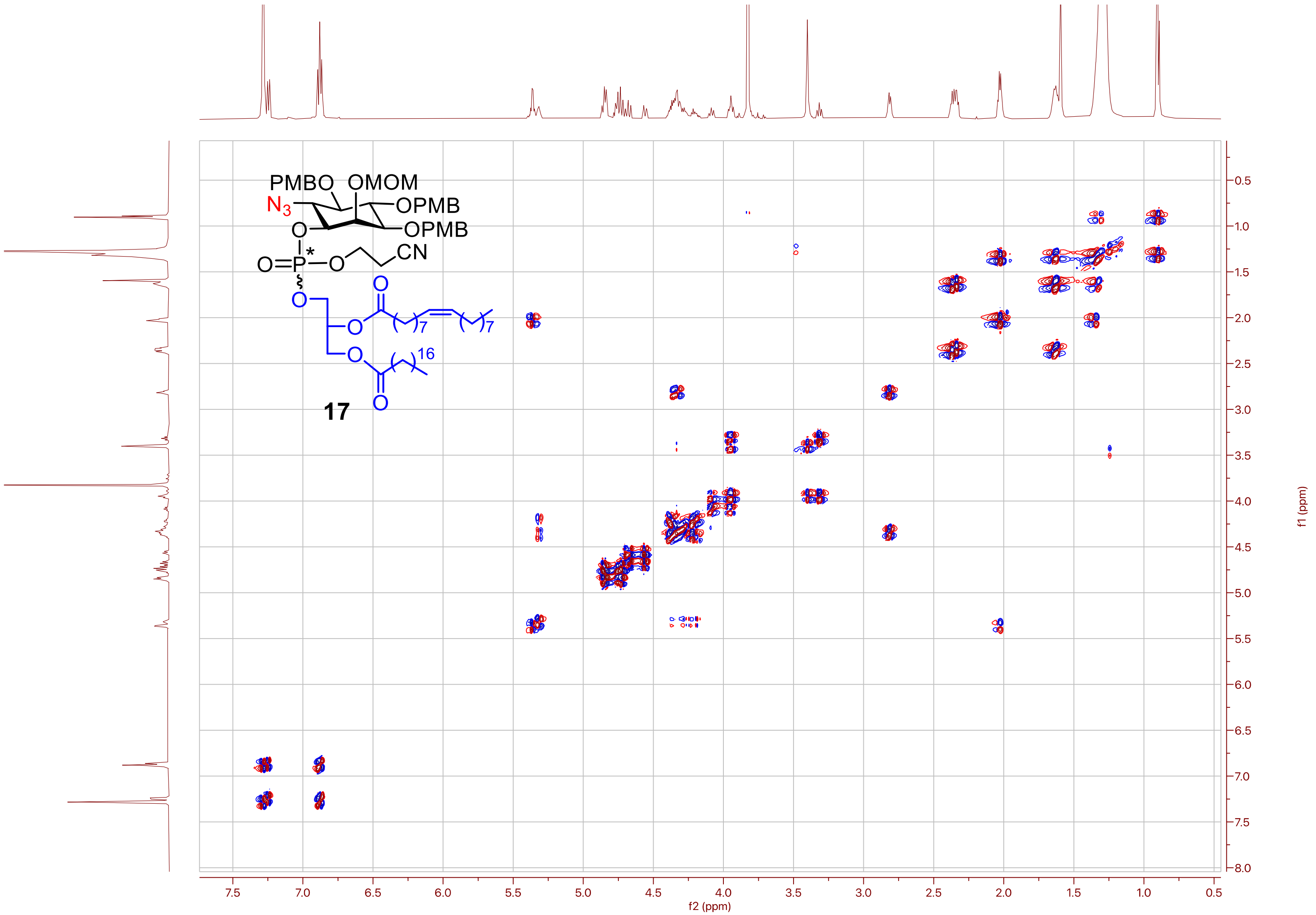

Figure S33. ^{1}H-^{1}H COSY spectrum of compound **17** (600 MHz, $CDCl_3$)

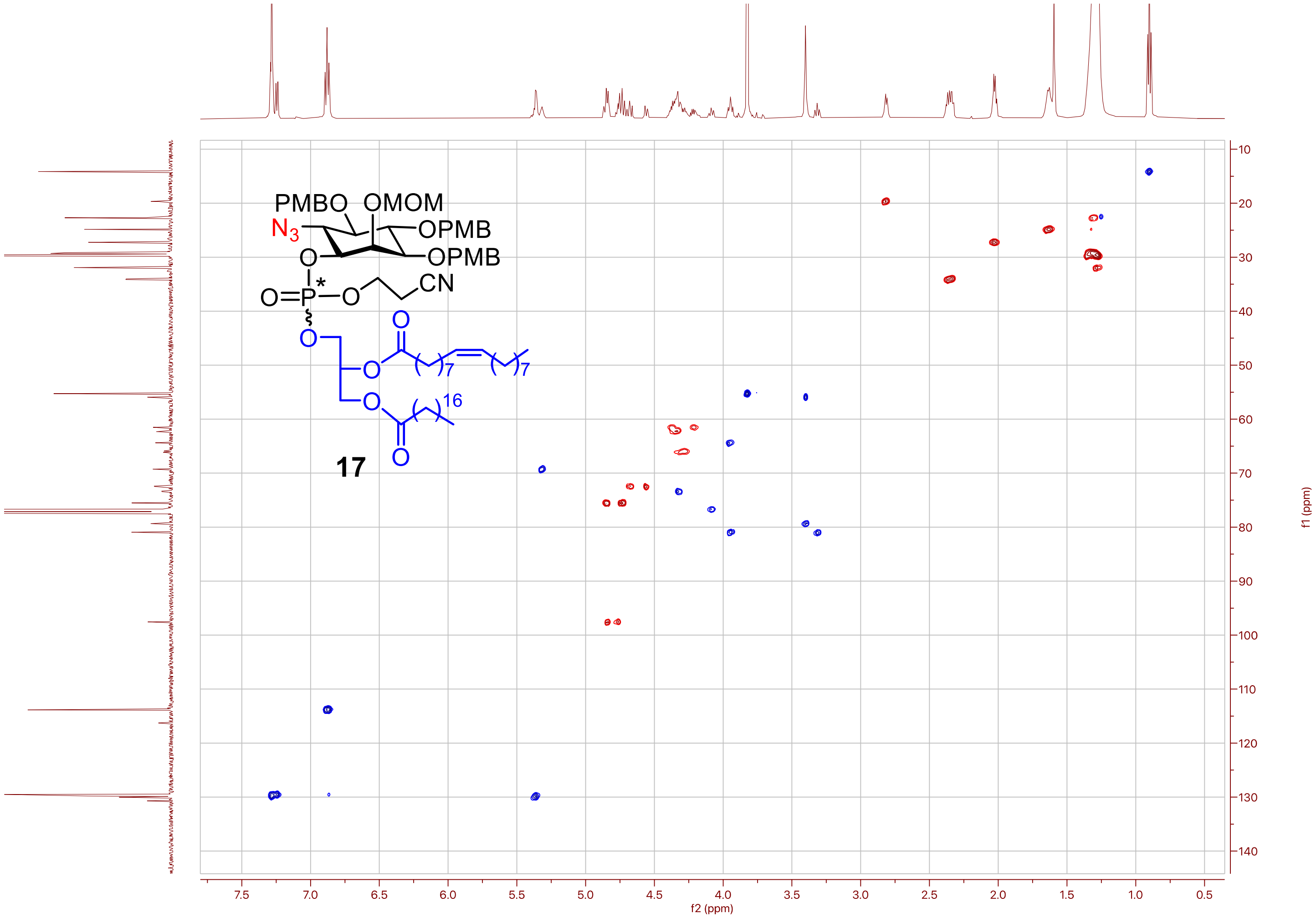

Figure S34. ^{1}H-^{13}C HSQC spectrum of compound **17** (600/151 MHz, $CDCl_3$)

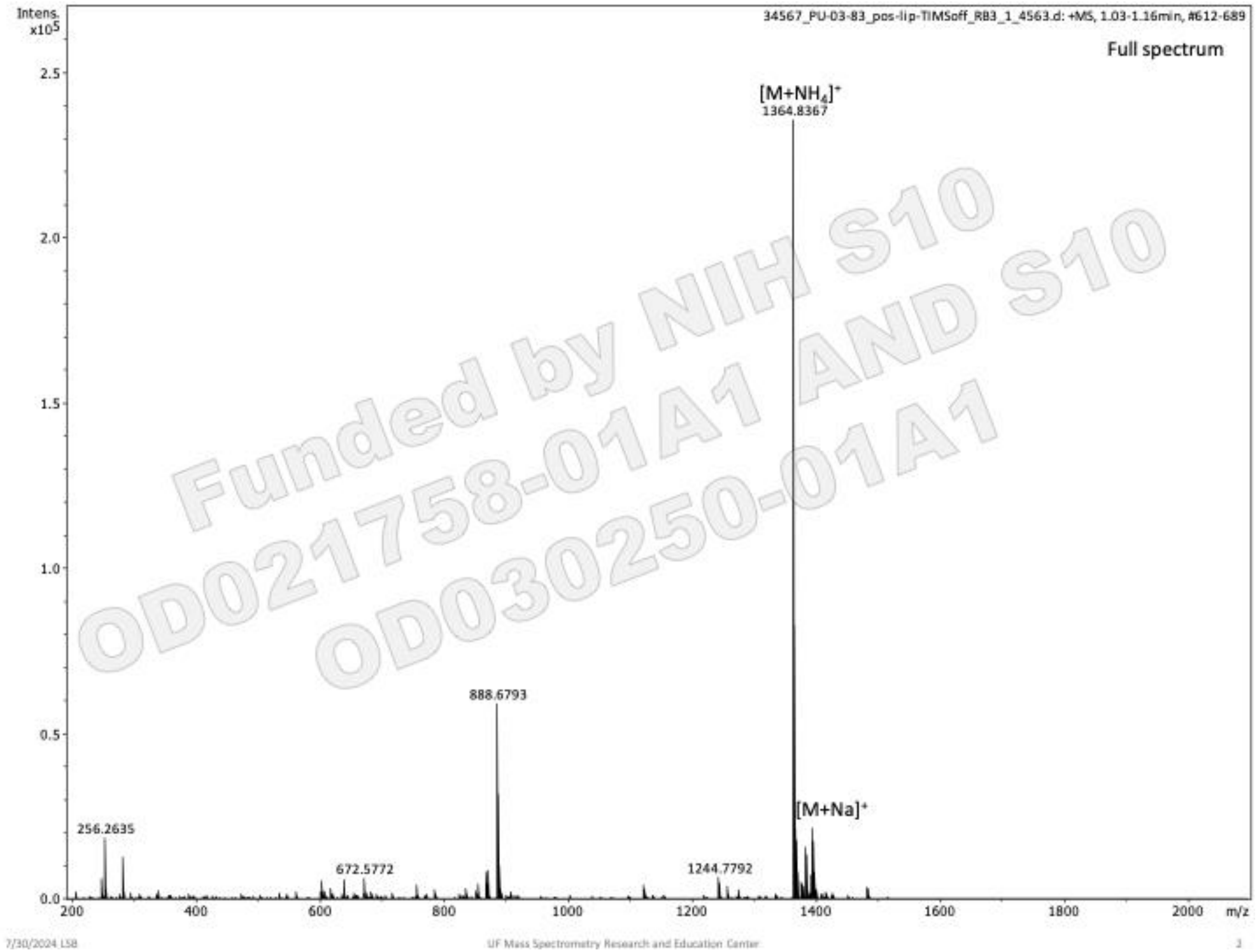

Figure S35: HRMS (ESI- TOF) spectrum of compound **17**

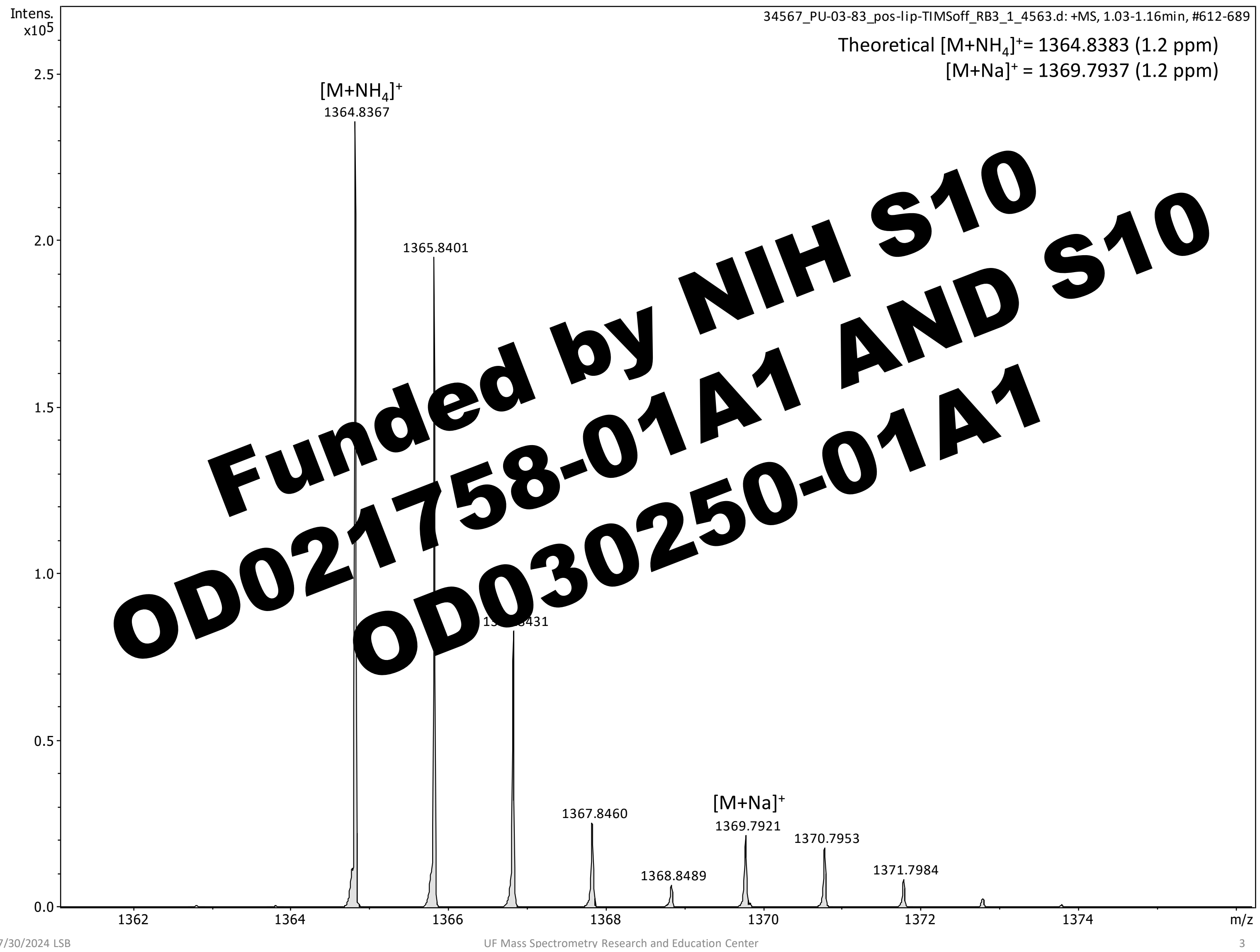

Funded by NIH S10 OD021758-01A1 AND S10 OD030250-01A1

Figure S36: Expanded HRMS (ESI- TOF) spectrum of compound **17**

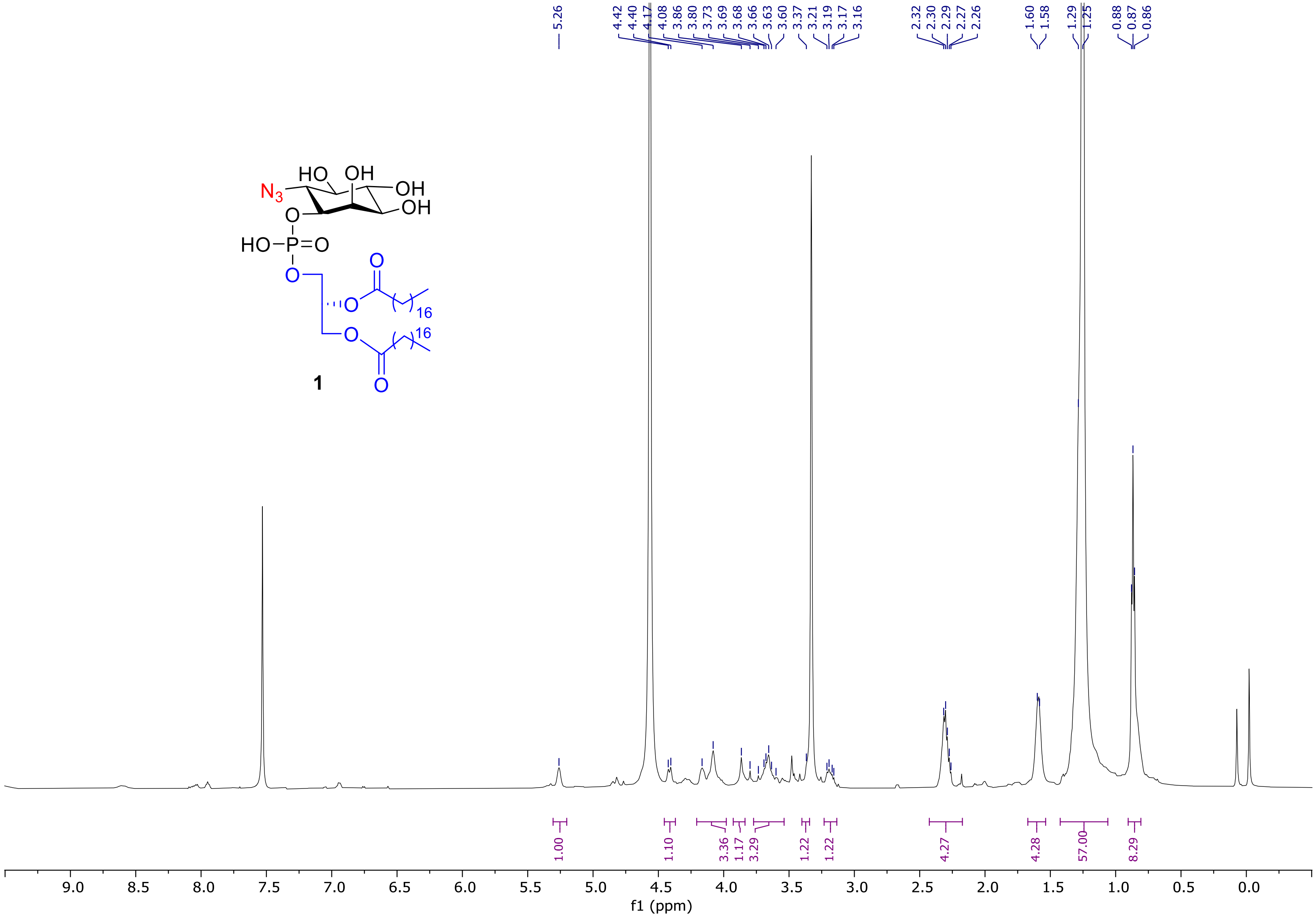

Figure S37: ^{1}H NMR spectrum of compound **1** (600 MHz, MeOD:$CDCl_3$ = 3:2)

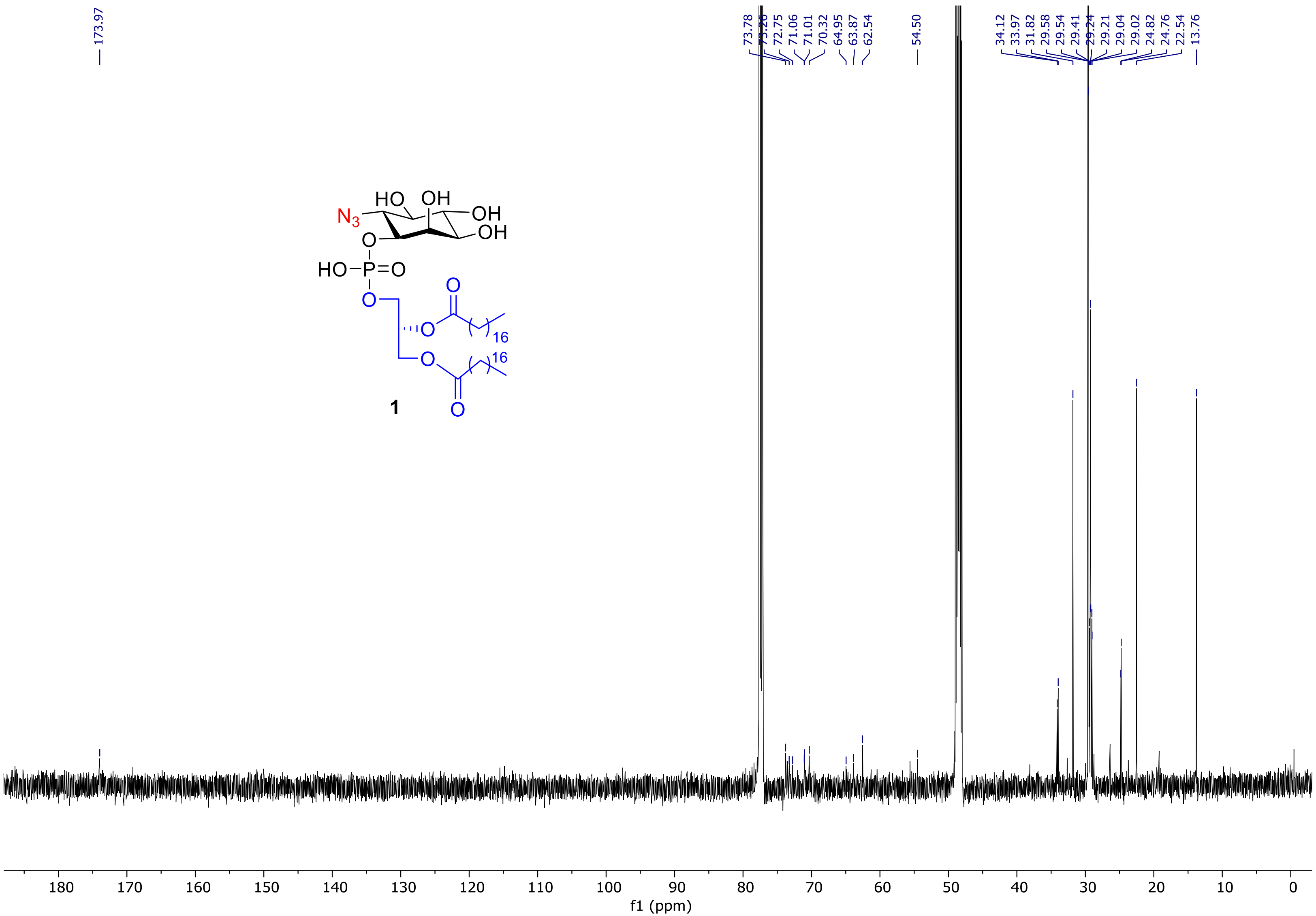

Figure S38: $^{13}C\{^{1}H\}$ NMR spectrum of compound **1** (151 MHz, MeOD:$CDCl_3$ = 3:2)

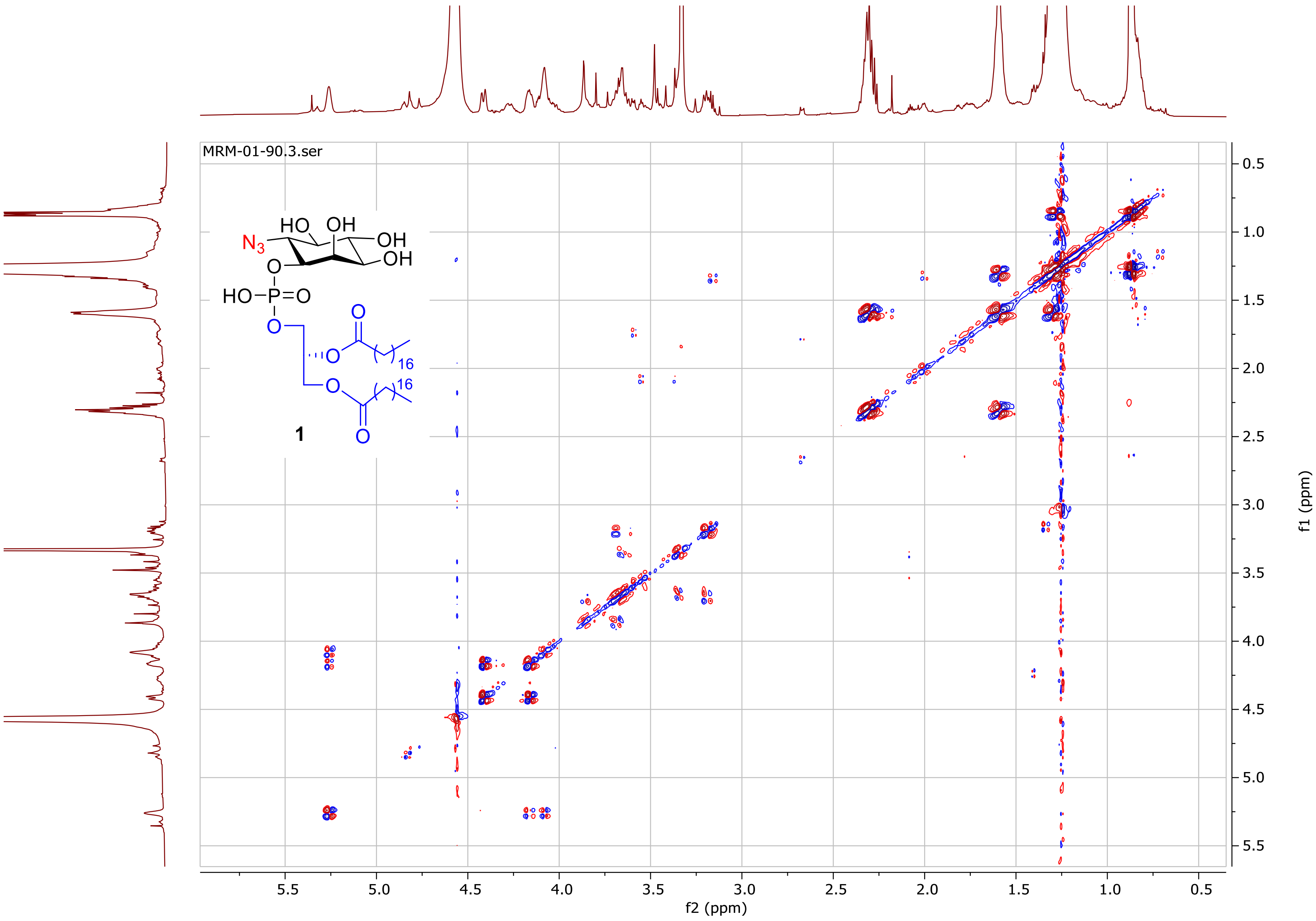

Figure S39: ^{1}H-^{1}H COSY spectrum of compound **1** (600 MHz, MeOD:$CDCl_3$ = 3:2)

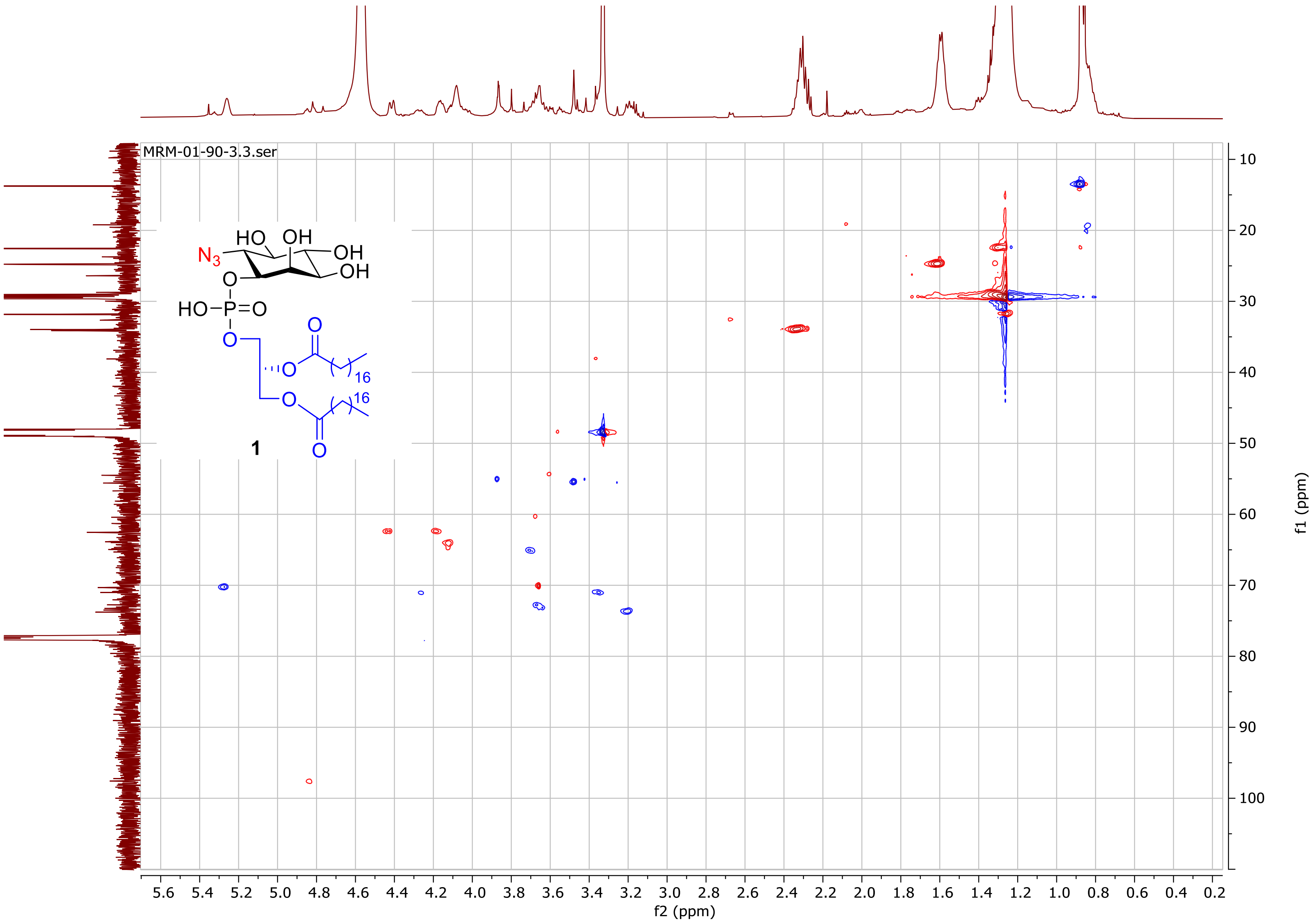

Figure S40: ^{1}H-^{13}C HSQC spectrum of compound **1** (600/151 MHz, MeOD:$CDCl_3$ = 3:2)

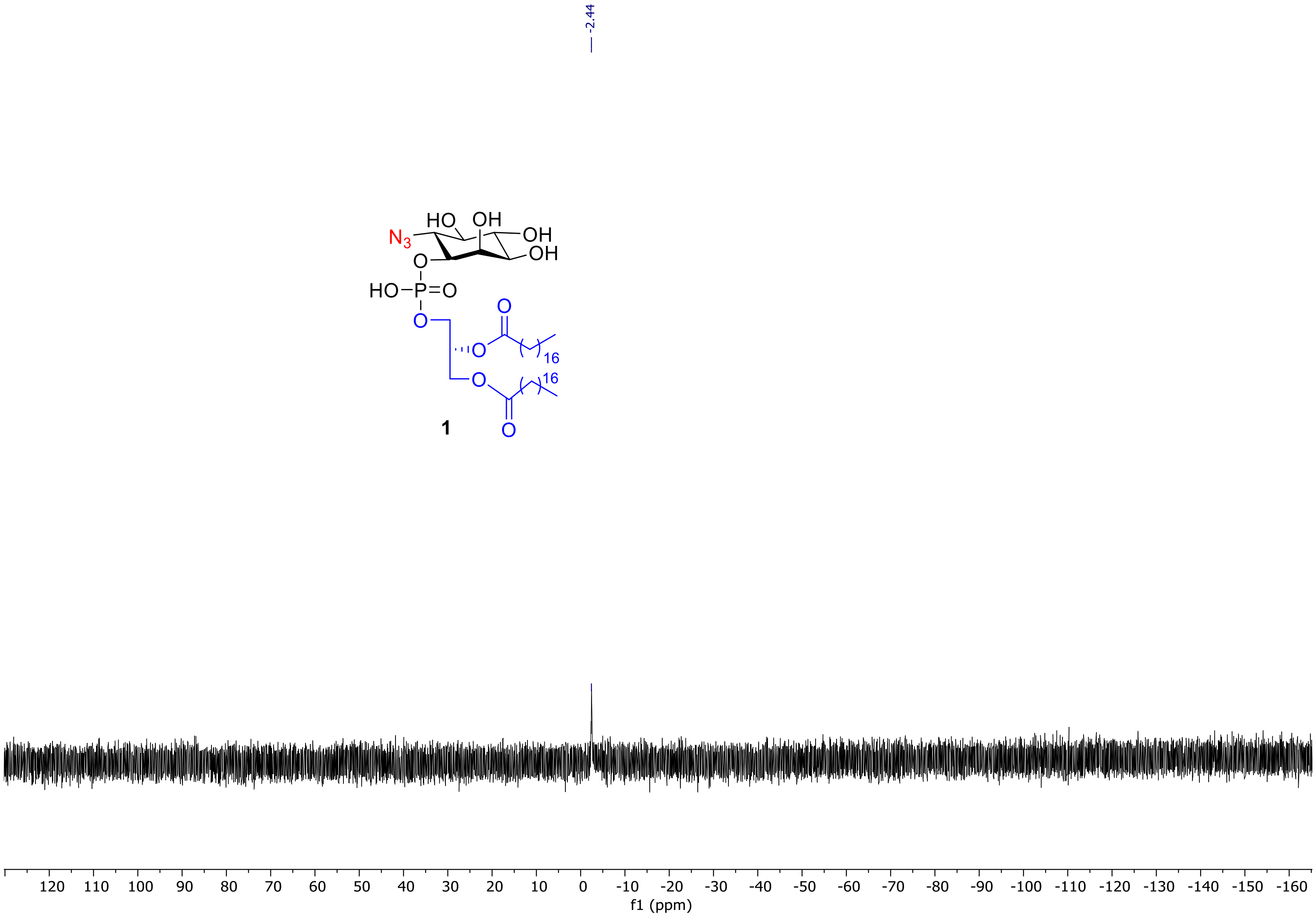

Figure S41: $^{31}P\{^1H\}$ NMR spectrum of compound **1** (162 MHz, MeOD:$CDCl_3$ = 3:2)

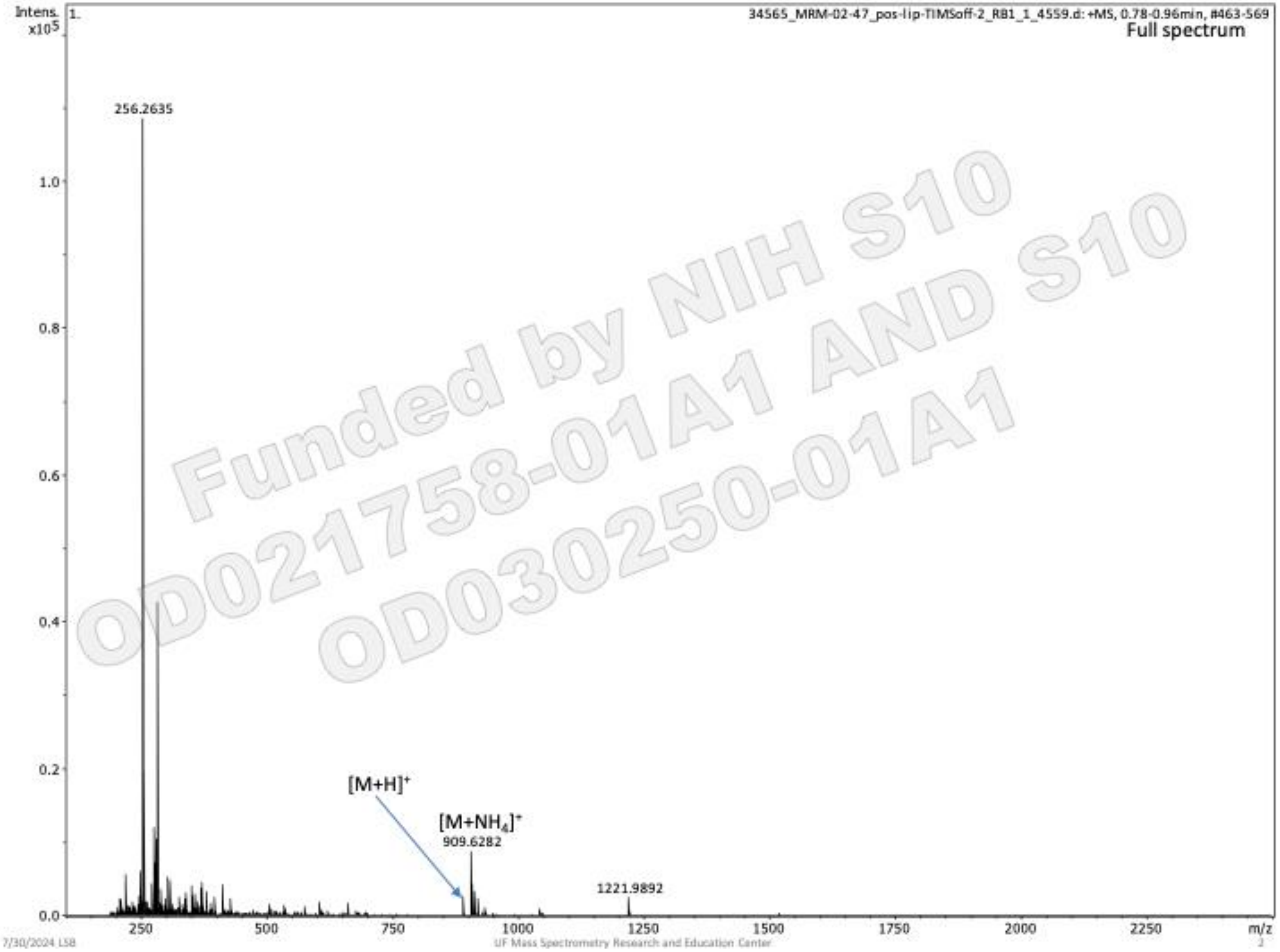

Figure S42: HRMS (ESI- TOF) spectrum of compound **1**

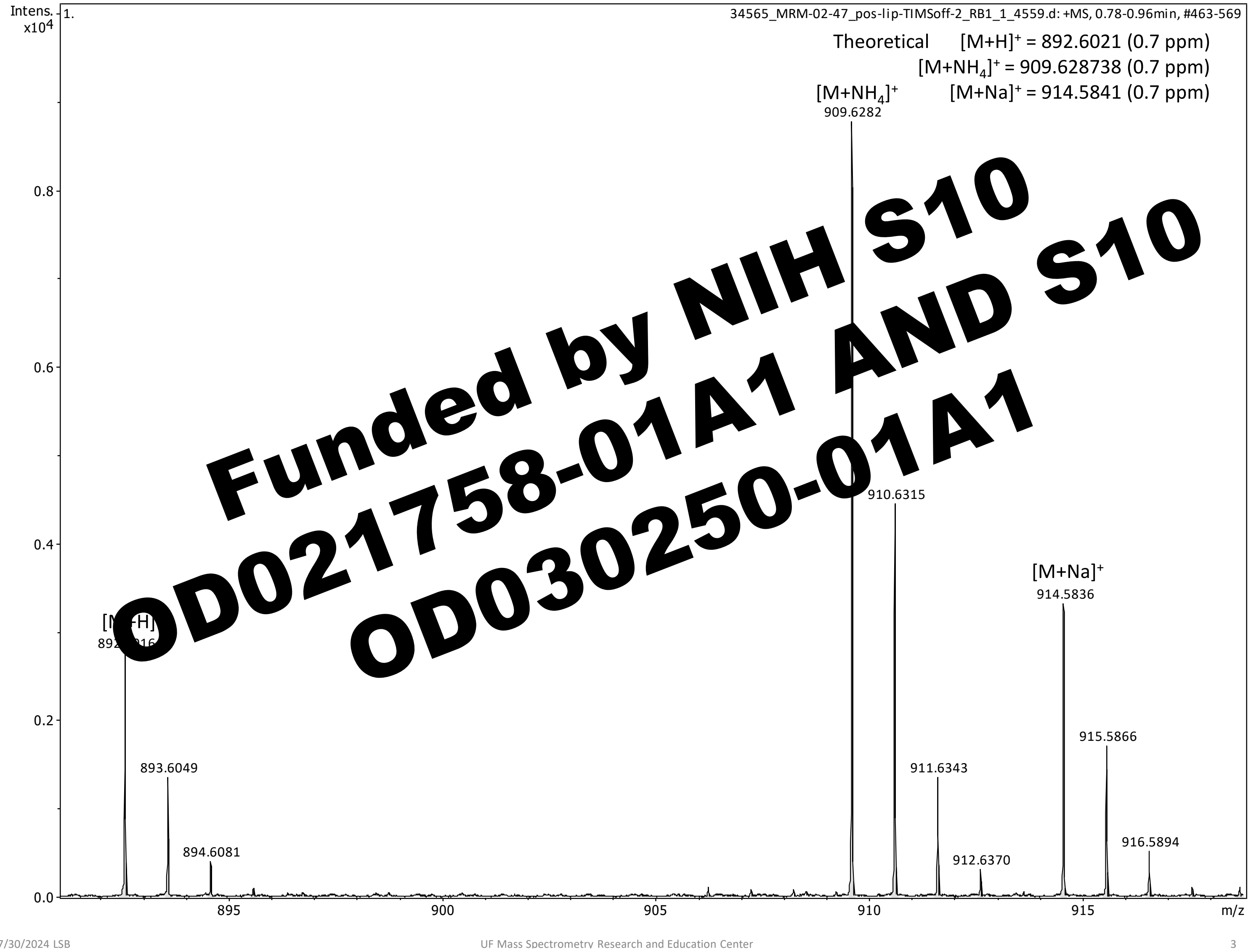

Funded by NIH S10 OD021758-01A1 AND S10 OD030250-01A1

Figure S43: Expanded HRMS (ESI- TOF) spectrum of compound **1**

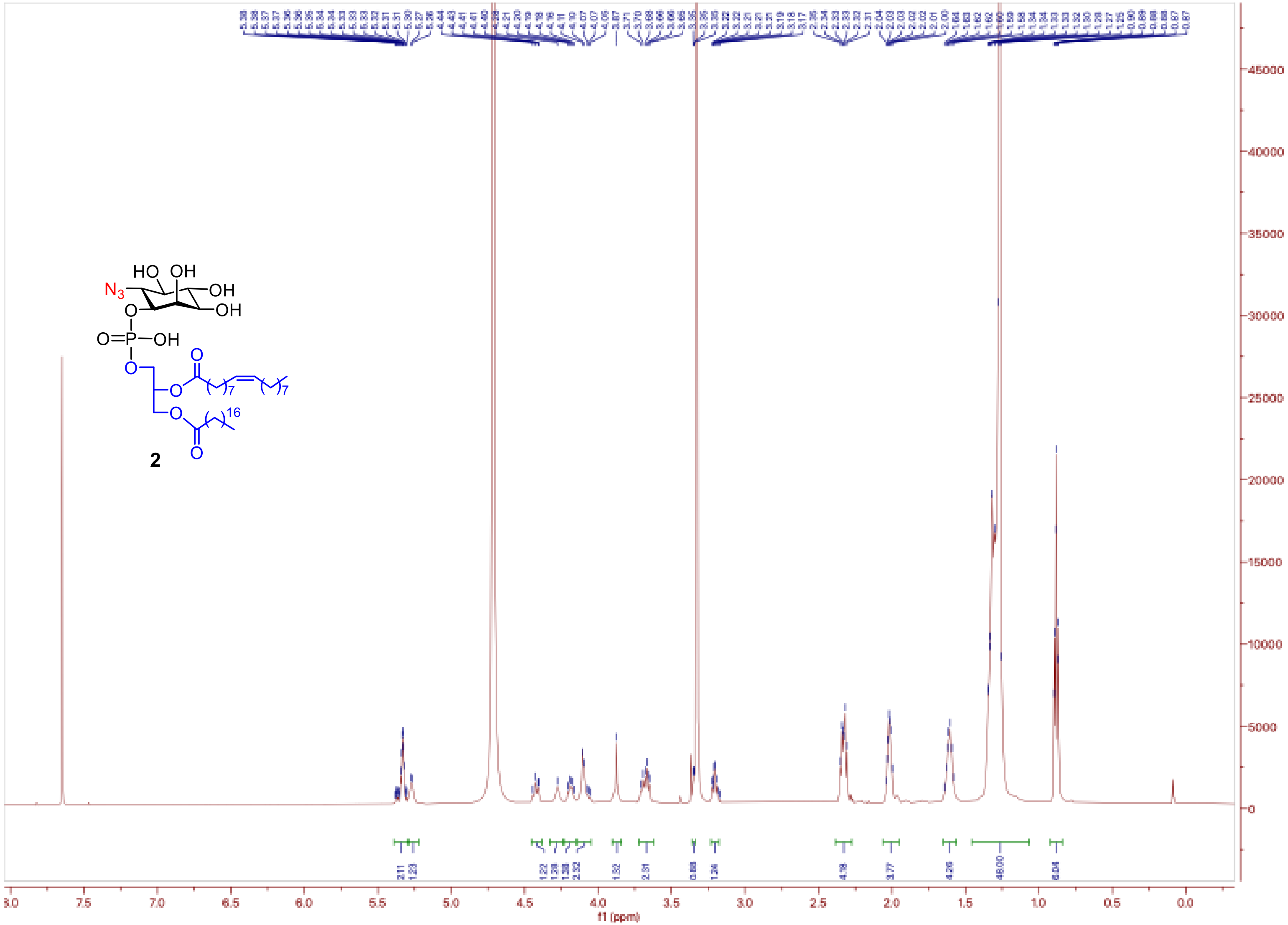

Figure S44. ^{1}H NMR spectrum of compound **2** (600 MHz, MeOD:$CDCl_3$ = 3:2)

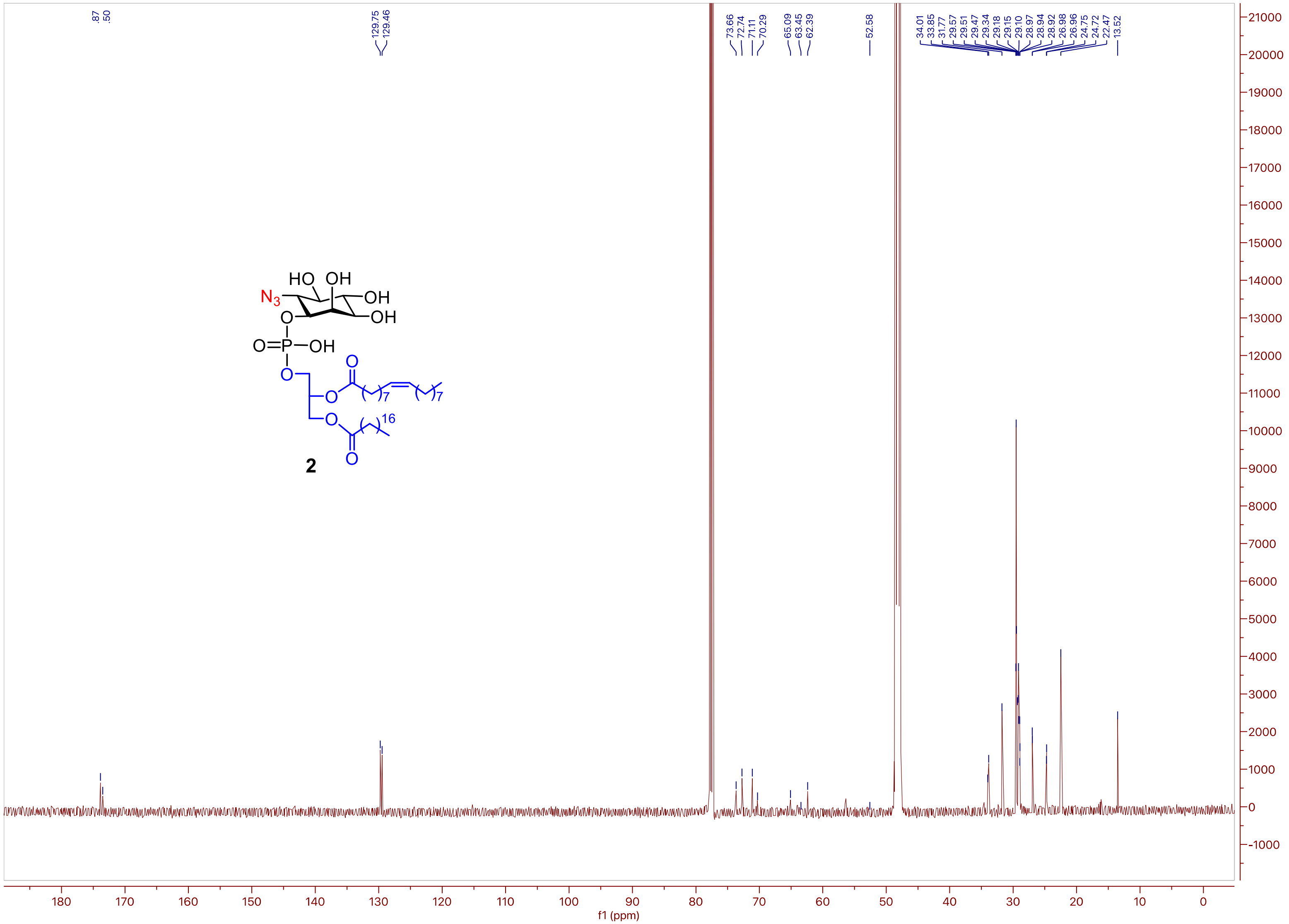

Figure S45. $^{13}C\{^{1}H\}$ NMR spectrum of compound **2** (151 MHz, MeOD:$CDCl_3$ = 3:2)

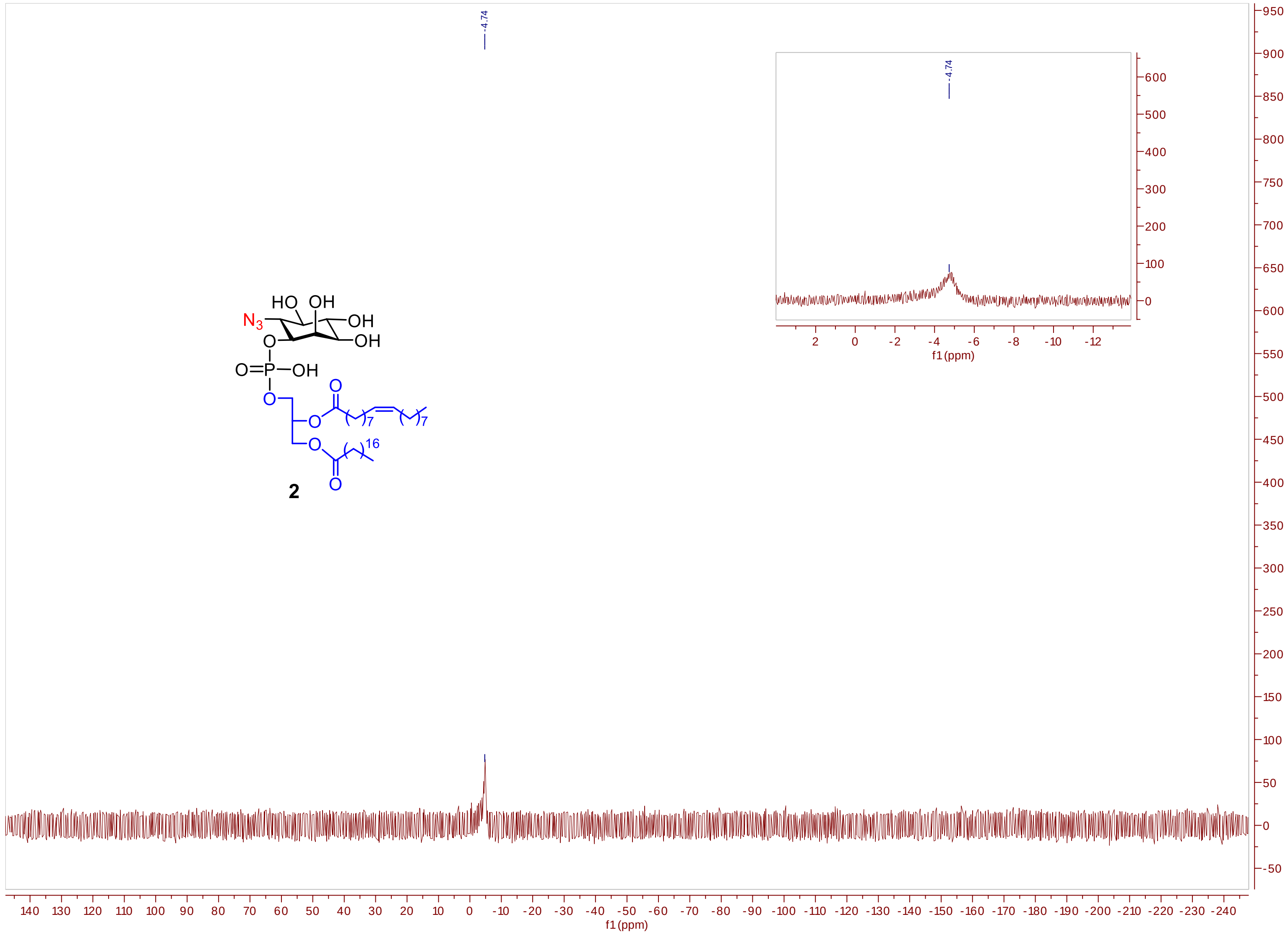

Figure S46. $^{31}P\{^{1}H\}$ NMR spectrum of compound **2** (243 MHz, MeOD:$CDCl_3$ = 3:2)

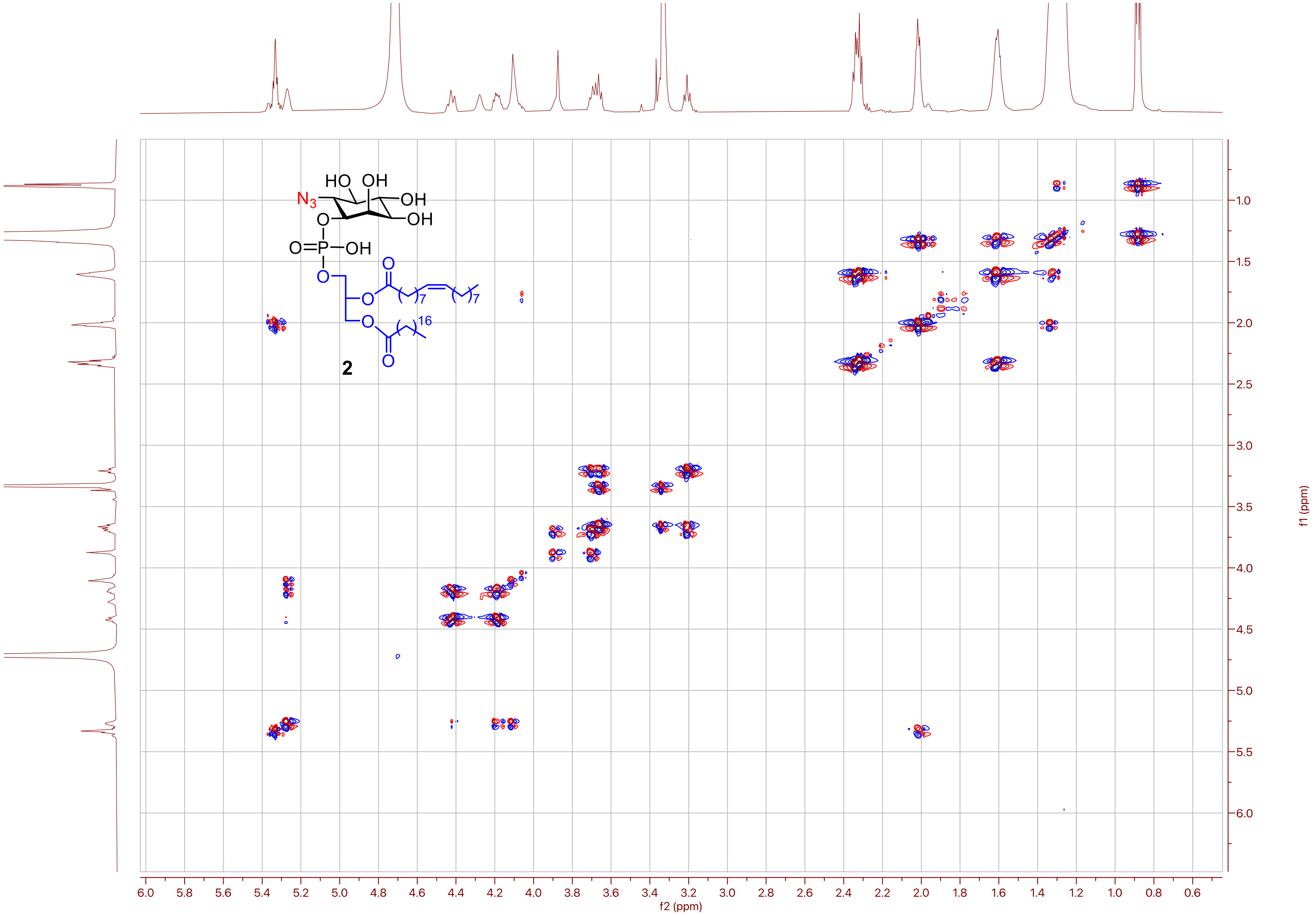

Figure S47. ^1H-^1H COSY spectrum of compound **2** (600 MHz, MeOD:$CDCl_3$ = 3:2)

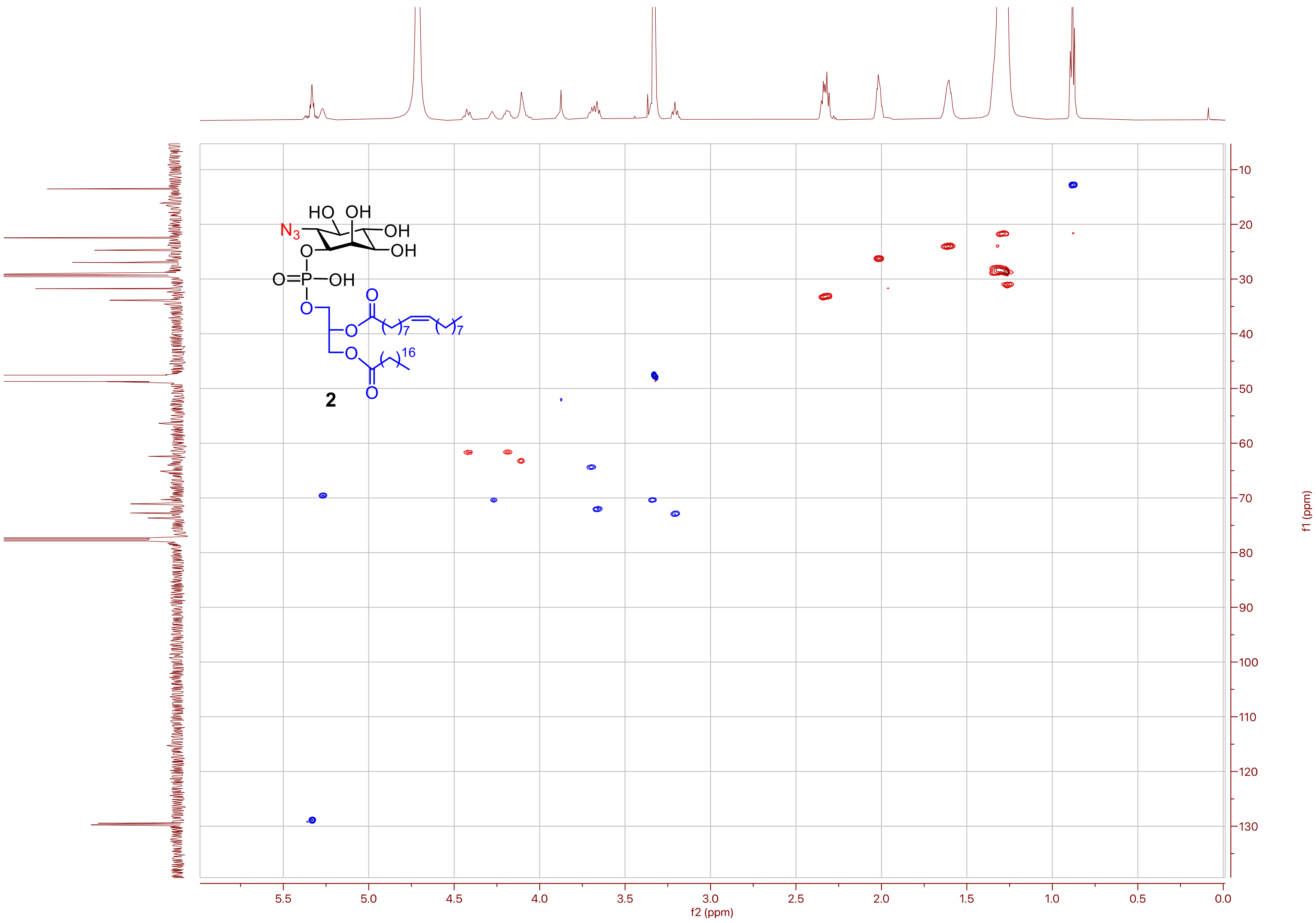

Figure S48. ^{1}H-^{13}C HSQC spectrum of compound **2** (600/151 MHz, MeOD:$CDCl_3$ = 3:2)

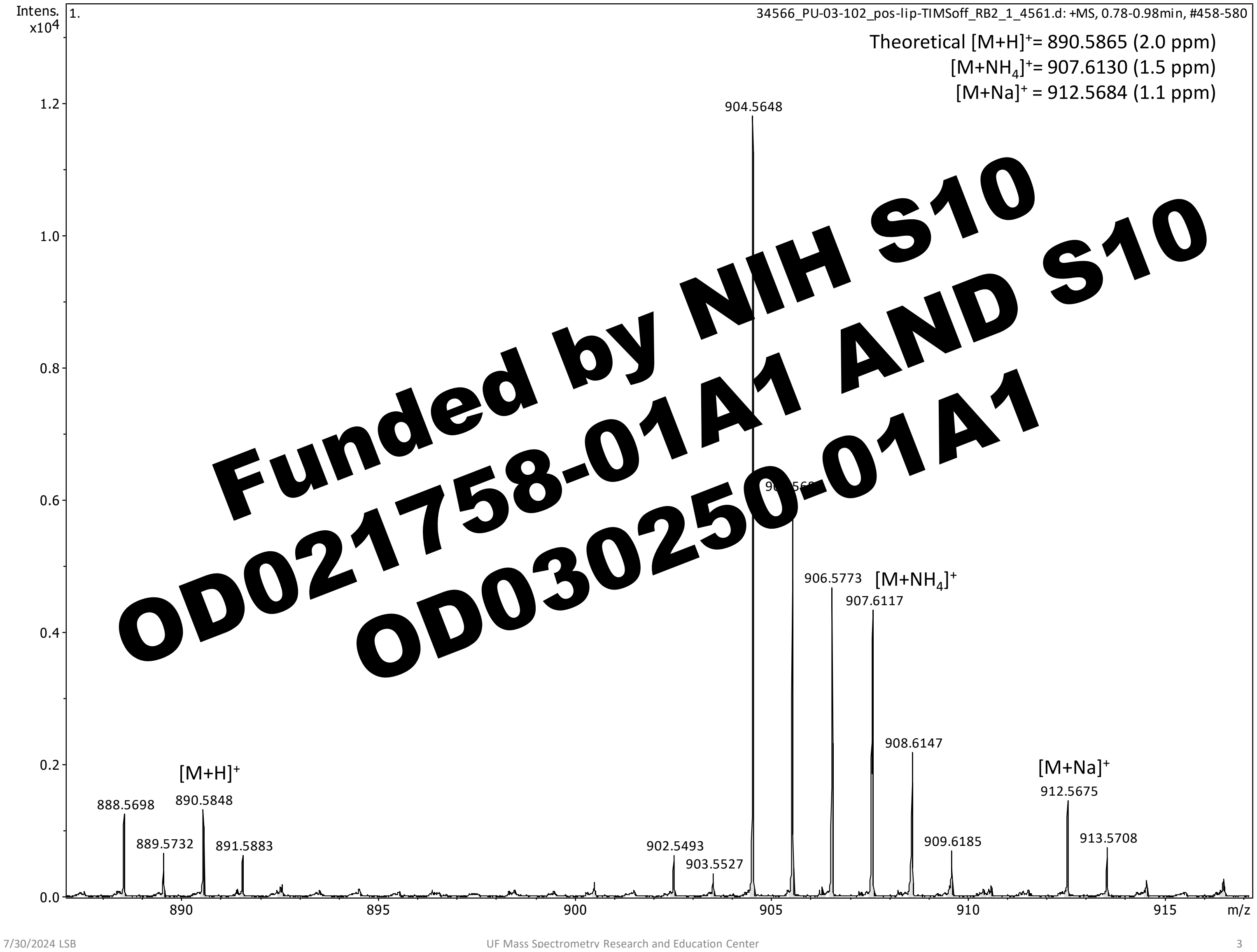

Funded by NIH S10 OD021758-01A1 AND S10 OD030250-01A1

Figure S49: HRMS (ESI- TOF) spectrum of compound **2**

A) B)

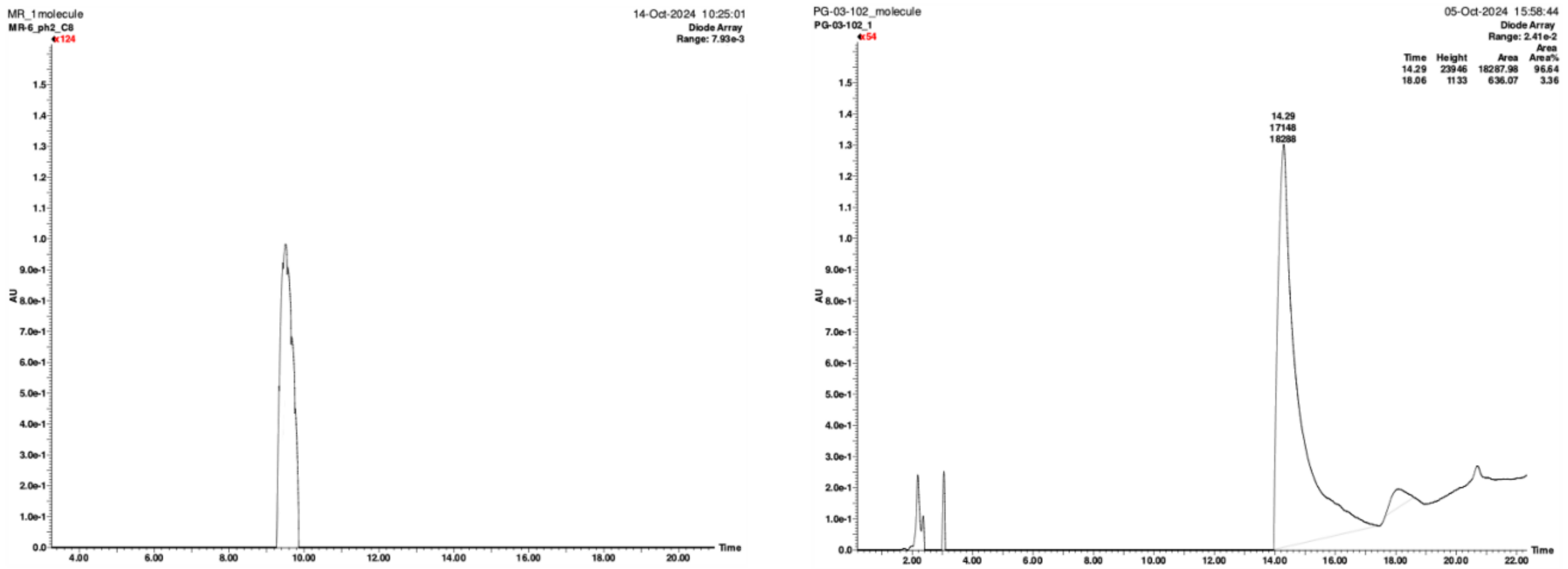

Figure S50. HPLC chromatograms of **1** (A) and **2** (B). HPLC conditions for **1**: a Waters e2695 Alliance and 2424 series HPLC instrument; ELSD detector with the masslynx 4.1 software and drift tube temperature of 25 °C and nitrogen gas pressure of 4 bar; Hypersil GOLDTM-C8 column (150 x 4.6 mm; 5 µ) at 30 °C; injection volume: 1 µL; mobile phase: methanol/*iso*-propanol (1/1), water, and 50 mM ammonium acetate adjusted (pH 3) in water (70:25:5, v/v/v) running in an isocratic mode with a flow rate of 1 mL/min for 30 min. HPLC conditions for **2**: a Waters e2695 Alliance and 2424 series HPLC instrument; ELSD detector with the masslynx 4.1 software and drift tube temperature of 25 °C and nitrogen gas pressure of 4 bar; Restek Ultra-C18 column (250 x 4.6 mm; 5 µ) at 30 °C; injection volume: 1 µL; mobile phase: water, 50 mM ammonium acetate (pH 5) in water, and methanol in gradient mode of 75:5:20 (v/v/v) for 10 min to 35:5:60 v/v for 20 min with a flow rate of 0.8 mL/min.
